# Evidence for Suzuki–Miyaura cross-couplings catalyzed by ligated Pd_3_-clusters: from cradle to grave†

**DOI:** 10.1039/d3sc06447f

**Published:** 2024-01-15

**Authors:** Neda Jeddi, Neil W. J. Scott, Theo Tanner, Simon K. Beaumont, Ian J. S. Fairlamb

**Affiliations:** a Department of Chemistry, University of York York YO20 5DD UK ian.fairlamb@york.ac.uk; b Department of Chemistry, Durham University South Road Durham DH1 3LE UK simon.beaumont@durham.ac.uk

## Abstract

Pd_*n*_ clusters offer unique selectivity and exploitable reactivity in catalysis. Understanding the behavior of Pd_*n*_ clusters is thus critical for catalysis, applied synthetic organic chemistry and greener outcomes for precious Pd. The Pd_3_ cluster, [Pd_3_(μ-Cl)(μ-PPh_2_)_2_(PPh_3_)_3_][Cl] (denoted as Pd_3_Cl_2_), which exhibits distinctive reactivity, was synthesized and immobilized on a phosphine-functionalized polystyrene resin (denoted as immob-Pd_3_Cl_2_). The resultant material served as a tool to study closely the role of Pd_3_ clusters in a prototypical Suzuki–Miyaura cross-coupling of 4-fluoro-1-bromobenzene and 4-methoxyphenyl boronic acid at varying low Pd ppm concentrations (24, 45, and 68 ppm). Advanced heterogeneity tests such as Hg poisoning and the three-phase test showed that leached mononuclear or nanoparticulate Pd are unlikely to be the major active catalyst species under the reaction conditions tested. EXAFS/XANES analysis from (pre)catalyst and filtered catalysts during and after catalysis has shown the intactness of the triangular structure of the Pd_3_X_2_ cluster, with exchange of chloride (X) by bromide during catalytic turnover of bromoarene substrate. This finding is further corroborated by treatment of immob-Pd_3_Cl_2_ after catalyzing the Suzuki–Miyaura reaction with excess PPh_3_, which releases the cluster from the polymer support and so permits direct observation of [Pd_3_(μ-Br)(μ-PPh_2_)_2_(PPh_3_)_3_]^+^ ions by ESI-MS. No evidence is seen for a proposed intermediate in which the bridging halogen on the Pd_3_ motif is replaced by an aryl group from the organoboronic acid, *i.e.* formed by a transmetallation-first process. Our findings taken together indicate that the ‘Pd_3_X_2_’ motif is an active catalyst species, which is stabilized by being immobilized, providing a more robust Pd_3_ cluster catalyst system. Non-immobilized Pd_3_Cl_2_ is less stable, as is followed by stepwise XAFS of the non-immobilized Pd_3_Cl_2_, which gradually changes to a species consistent with ‘Pd_*x*_(PPh_3_)_*y*_’ type material. Our findings have far-reaching future implications for Pd_3_ cluster involvement in catalysis, showing that immobilization of Pd_3_ cluster species offers advantages for rigorous mechanistic examination and applied chemistries.

## Abstract 2

Pd_*n*_ clusters offer unique selectivity and exploitable reactivity in catalysis. Tethering Pd_3_ clusters to a resin unlocks compelling new insight into the speciation of Pd during catalytic turnover for Suzuki–Miyaura cross-couplings.

## Graphical abstract

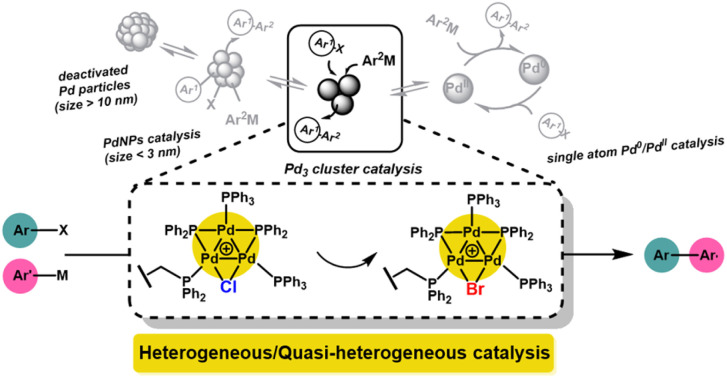

## Introduction

Suzuki–Miyaura cross-coupling (SMCC) reactions are ubiquitous in applied chemical synthesis laboratories around the world, forming a keystone in the synthesis of an eclectic array of high value organic products.^1^ Palladium is the metal catalyst of choice for many of these transformations, although to be sustainable this precious metal ought to be used responsibly, *i.e.* used at low catalyst loadings, and be efficiently recovered and recycled, particularly in large scale chemical processes.^2^ Advances in the design of well-defined ligand systems (*e.g.* the Buchwald-type ligands such as XPhos, SPhos and many others) have positively contributed towards applied SMCC reaction processes.^3^ On the other hand, multinuclear Pd species, from small Pd_*n*_ clusters to Pd nanoparticles, offer unique reactivity and activity in SMCC reactions, as well as other cross-coupling chemistries.^4^ Immobilized-Pd^5^ or the use of supported Pd nanoparticles allow for more effective^6^ catalyst recycling, particularly when used in conjunction with magnetic co-additives such as iron.^7^ Moving towards 2030, one can argue that simply improving the activity and recyclability of Pd catalysts is not enough. However, if one can alter reaction outcomes of cross-coupling reactions based on unique reactivity of a reproducible, well-defined multinuclear Pd species, then that could be a highly exploitable tool in synthetic chemistry.^8^ In this context, the identification of Pd_3_ clusters of the type [Pd_3_(μ-Cl)(μ-PPh_2_)_2_(PPh_3_)_3_]X (X can be a variety of coordinating or non-coordinating anions; structures originally discovered and characterized by Coulson and Dixon)^9^ as competent, highly active and selective catalysts for cross-coupling reactions has prompted several research groups to exploit their distinctive and unique behavior.^4*d*,10^ SMCC reactions with di- and tetra-nuclear Pd complexes have also been reported, although with less detailed mechanistic evaluation.^11^ A guiding example with Pd_3_ is the cross-coupling of 2,4-dibromopyridine, where typical mononuclear Pd species containing phosphine ligands exhibit C2 site-selectivity.^12^ On the other hand, [Pd_3_(μ-Cl)(μ-PPh_2_)_2_(PPh_3_)_3_]X species (X = Cl or OAc) exhibit C4 site-selectivity.^10*b*^ Indeed, higher order stabilized Pd nanoparticles exhibit similar behavior.^13^ Although the mechanism for this site-selectivity switch is not fully known, the ability to program either reaction outcome by the choice of catalyst provides synthetic chemists with a powerful tool for wider applications.

In 2017, Li *et al.* reported the reactivity and behavior of [Pd_3_(μ-Cl)(μ-PPh_2_)_2_(PPh_3_)_3_]X species in SMCC reactions, suggesting it to be a highly active catalyst for the cross-coupling of an array of coupling partners, including aryl chlorides.^4*d*^ The latter point is remarkable, as clearly the aggregated cyclic Pd_3_ cluster increases the reactivity of this Pd-phosphorus based catalyst system. Moreover, the authors suggested that there was an inversion of the steps in SMCC reactions catalyzed by [Pd_3_(μ-Cl)(μ-PPh_2_)_2_(PPh_3_)_3_]X species (X = SbF_6_). Despite the mechanistic complexity of typical SMCC reactions, *i.e.* the difficulty in elucidating Pd nuclearity and speciation (both Pd and B), the scientific community broadly agrees with a sequence of oxidative addition, transmetallation and reductive elimination steps. However, a transmetallation-first step was proposed for the action of [Pd_3_(μ-Cl)(μ-PPh_2_)_2_(PPh_3_)_3_]X species, which is followed by oxidative addition (σ-bond metathesis suggested) and reductive elimination at the intact Pd_3_ cluster center. Thus it would appear that [Pd_3_(μ-Cl)(μ-PPh_2_)_2_(PPh_3_)_3_]X operates in a mechanistically distinct manner to typical mononuclear Pd catalysts, particularly Pd_0_(PPh_3_)*n* catalyst systems. Indeed, Fairlamb *et al.* demonstrated the normally expected sequence is only observed for Pd^0^(PPh_3_)_*n*_ where *n* > 3 in reactions of 2-bromopyridine^10*a*^ or 2,4-dibromopyridine^10*b*^ with aryl boronic acids. When *n* < 3 higher order Pd_*n*_ clusters play a key role.

Despite the work conducted on [Pd_3_(μ-Cl)(μ-PPh_2_)_2_(PPh_3_)_3_]X catalyst species, there is limited information available on the pathways for catalyst activation, understanding productive catalyst turnover and potential pathways for catalyst deactivation, *i.e.* from cradle to grave. Indeed, apart from our preliminary kinetic studies,^10*a*^ there is very little reported on the speciation and kinetic behavior of [Pd_3_(μ-Cl)(μ-PPh_2_)_2_(PPh_3_)_3_]X species in catalytic cross-coupling reactions. Preliminary X-ray Absorption Spectroscopy (XAS) data on the behavior of [Pd_3_(μ-Cl)(μ-PPh_2_)_2_(PPh_3_)_3_]X species reported by Li *et al.* show changes,^4*d*^ however the EXAFS data were not fitted to structures proposed making speciation assignments difficult. This prior work, in addition to Pd_3_Cl_2_'s distinct selectivity and high activity in cross couplings, prompted us to conduct further XAS studies and mechanistic experiments on the Pd_3_ cluster system.

**Fig. 1:**
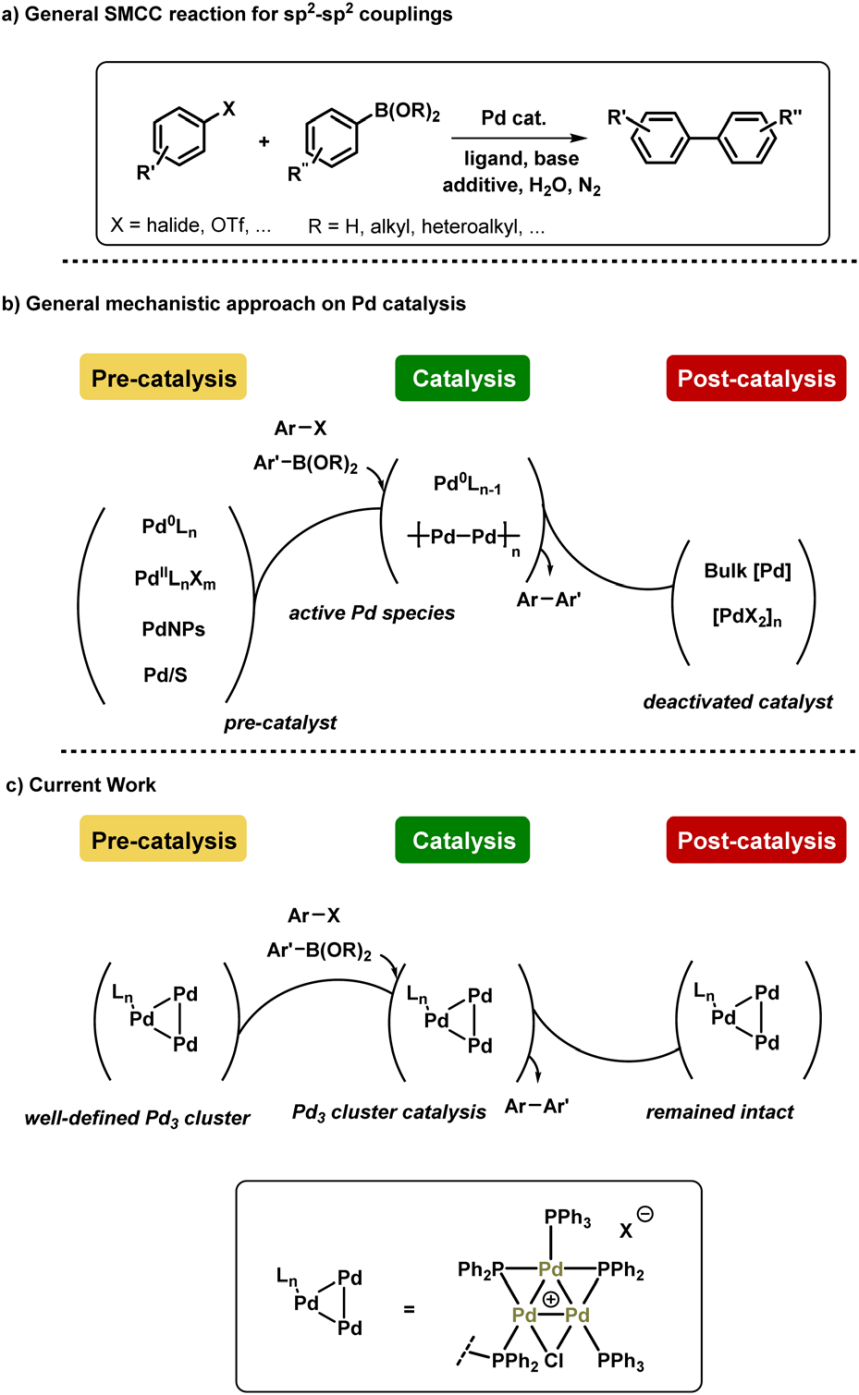
(a) General Pd-catalyzed SMCC reaction scheme, (b) the mechanistic approach on Pd catalyst speciation during and after catalysis, (c) and current work showing the higher order Pd_3_ catalyst robustness during and after catalysis.

Herein, we report our findings on the behavior of a phosphine-supported (immobilized) [Pd_3_(μ-Cl)(μ-PPh_2_)_2_(PPh_3_)_2_(PPh_2_)-Ar-supported]Cl species in SMCC reactions. Various immobilized systems have been applied to SMCC reactions and are reviewed elsewhere,^14^ however, we have focused on a supported catalyst system to facilitate our structural study of the intact Pd_3_ cluster species in a manner that [Pd_3_(μ-Cl)(μ-PPh_2_)_2_(PPh_3_)_3_]X cannot alone. We have further conducted studies using [Pd_3_(μ-Cl)(μ-PPh_2_)_2_(PPh_3_)_3_]Cl as the catalyst system, so that there is a degree of comparability enabling broader conclusions to be reached. In a wider context, the combination of catalyst immobilization strategies and in solution structural characterization (XAS) presented in this study provides a robust approach for discriminating reaction participants and evaluating Pd speciation in SMCC reactions. In doing so we signpost how this approach can be invaluable for furthering our understanding of this important class of reactions, while recognizing the complexity of the catalyst speciation (summarized in Fig. 1).

## Results and discussion

### Synthesis and characterization of immobilized-Pd_3_Cl_2_3

**Scheme 1:**
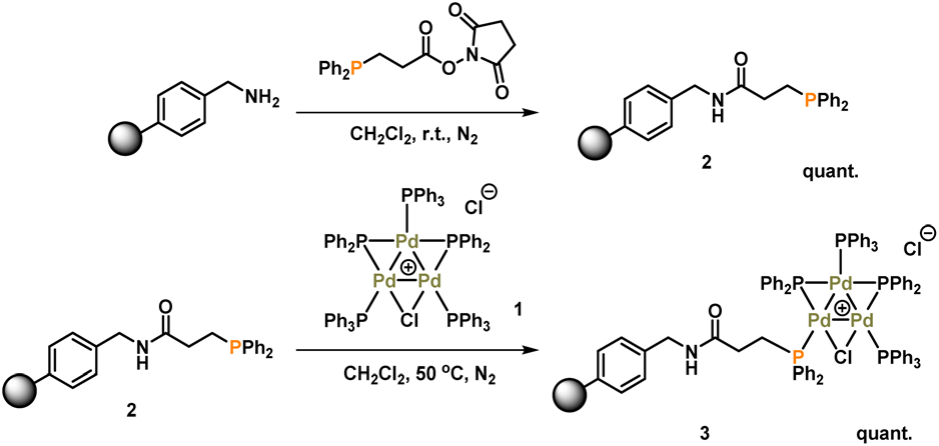
Synthesis of immobilized-Pd_3_Cl_2_ cluster 3.

The synthesis of [Pd_3_(μ-Cl)(μ-PPh_2_)_2_(PPh_3_)_3_]Cl 1 (referred to as Pd_3_Cl_2_1) can be accomplished by the reaction of *trans*-PdCl_2_(PPh_3_)_2_ with H_2_ in aniline at 90 °C.^10*a*^ The immobilization of 1 used of an (aminomethyl)polystyrene resin (∼1.5 mmol g^−1^ amine loading, 200–400 mesh), which was treated with a linkable *N*-succinimidyl 3-(diphenylphosphino)-propionate phosphine ligand to afford 2, which was then directly reacted with Pd_3_Cl_2_1, giving the immobilized-Pd_3_Cl_2_3 (referred to as immob-Pd_3_Cl_2_3) (Scheme 1). The non-polar polystyrene resin was hypothesized to provide a suitable environment in which to interact with the Pd_3_ cluster, since the PPh_3_/PPh_2_ groups give it a non-polar hydrophobic chemical environment, particularly above and below the plane of the triangular Pd_3_ structural motif. Furthermore, we anticipated that these interactions protect the Pd_3_ cluster from the harshness of the basic environment induced by the use of *n*-Bu_4_NOH.

**Fig. 2:**
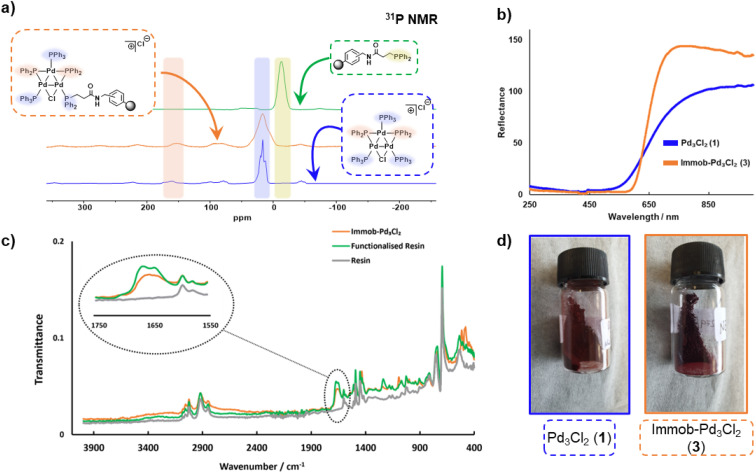
(a) Solid-state ^31^P NMR spectral data for Pd_3_Cl_2_1, the functionalized resin and the immob-Pd_3_Cl_2_3 (at 10 kHz); (b) reflectance UV-vis spectra of Pd_3_Cl_2_1 and immob-Pd_3_Cl_2_3; (c) overlay ATR-IR spectral of (aminomethyl) polystyrene resin (blue), phosphine-functionalized resin 2 (green) and immob-Pd_3_Cl_2_3 (orange); (d) material appearance of 1 (dark red) and 3 (dark purple).

The connectivity of the Pd_3_Cl_2_ cluster motif during the immobilization process was confirmed by a solid-state ^31^P NMR experiment (Fig. 2a). The ^31^P NMR spectra of Pd_3_Cl_2_1 and immob-Pd_3_Cl_2_3 confirm the presence of the triangular Pd_3_ motif anchored with functionalized (aminomethyl)polystyrene resin; similar ^31^P chemical shifts are seen for the phosphorus chemical environments. For Pd_3_Cl_2_1 we observed a broad peak with some fine structure at *ca. δ* −7 to 43 ppm, which possesses spinning side bands at *δ* −44 and 80 ppm. These signals are attributed to the distal and proximal phosphines ligated to Pd. A similar but broadened ^31^P NMR spectrum is seen for immob-Pd_3_Cl_2_3 with the same main feature around −7 to 43 ppm, which given the sensitivity of ^31^P NMR points to the presence of the same phosphorous environments. The phosphine functionalized resin prior to addition of the Pd cluster is quite different with a singlet centered at −13 ppm with two side bands (Fig. 2a). None of this signal at −13 ppm remains in immob-Pd_3_Cl_2_, showing that all the resin bound phosphine is interacting with the Pd cluster. As expected, free PPh_3_ and excess Pd_3_Cl_2_ are observed in the solution phase by ^1^H and ^31^P NMR after the immobilization step (Fig. S3 and S4†).

A similar, but broader peak is seen for immob-Pd_3_Cl_2_3, along with spinning side bands. The ^31^P NMR chemical shifts of the coordinating terminal PPh_3_ ligands share similarities with those seen in solution for Pd_3_Cl_2_1. The signals for the two bridging PPh_2_ environments in Pd_3_Cl_2_1 are distinctive, centered at *δ* 162, with spinning side bands at *δ* 220 and 100 (note: there is further evidence of extended anisotropy at wider chemical shifts within this spectral data). Importantly, these chemical shifts closely match the signals observed for immob-Pd_3_Cl_2_3, noting the broader signals, which we associate with the subtly different pore environments of the resin support, and the substituted possibility of phosphine being distal or proximal to chloride. However, there is no evidence to show which phosphine (*i.e.* distal or proximal position to chloride) undergoes substitution or how many substitutions occur within the cluster. Importantly, the reflectance UV-vis spectrum of immob-Pd_3_Cl_2_3 (red-shifted) shares similarity with Pd_3_Cl_2_1 (Fig. 2b), confirming the structure of the triangular Pd_3_ motif following immobilization. There is a physical difference in the color of these materials – immob-Pd_3_Cl_2_3 is dark purple whereas Pd_3_Cl_2_1 is of a dark red appearance (Fig. 2d). Attenuated total reflectance infrared spectroscopic analysis of immob-Pd_3_Cl_2_3 revealed the presence of the vibrational peaks associated with the amide carbonyl group, that overlay the amide group in the spectrum of the phosphine-functionalized resin 2 between 1640–1680 cm^−1^ (Fig. 2c). This implies the preservation of the amide linkage in immob-Pd_3_Cl_2_3 and that the phosphine remained tethered to the polystyrene during the Pd_3_ immobilization reaction. These are not present in the stating resin material (no amide carbonyl). The retention of the amide linkage is important as it confirms the tethered phosphine was not released from the polystyrene anchor such that it was able to act as a new solution phase ligand and that any exchange really was the result of interaction of Pd_3_Cl_2_1 with the resin bound phosphine 2.

Summarizing the initial characterization of the immob-Pd_3_Cl_2_3, ^31^P SSNMR provides evidence of the Pd_3_ motif remaining, and no significant other P containing species being present, UV-vis data show a lack of change to the metal–ligand interactions and ATR-IR confirms, *via* presence of the carbonyl, that the linker unit is intact.

### Suzuki–Miyaura cross-coupling (SMCC) catalysis

**Fig. 3:**
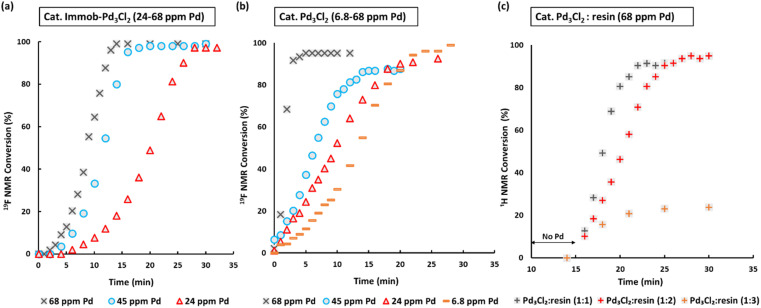
SMCC reaction kinetic profiles using immob-Pd_3_Cl_2_3 and Pd_3_Cl_2_1 using different catalyst loadings (monitored by ^1^H and ^19^F NMR; 1,3,5-trimethoxybenzene and 4,4′-difluorobenzophenone as internal standards): (a) immob-Pd_3_Cl_2_3; (b) Pd_3_Cl_2_1; (c) Pd_3_Cl_2_1 with exogenous phosphine resin support 2 (in a ratio of 1 : 1, to 1 : 2 and 1 : 3), with the reaction triggered by addition of Pd_3_Cl_2_ at *t* = 15 minutes. Note: the Pd concentration of 68 ppm (moles) equates to a 0.34 mol% catalyst loading with respect to 4 [the ArBr]. Details concerning sampling, NMR analysis and reproducibility are included in the ESI.†

The reactivity of immob-Pd_3_Cl_2_3 was examined in a benchmark SMCC reaction of 4-fluoro-bromobenzene 4 and 4-methoxyphenyl boronic acid 5 under optimized reaction conditions (Table 1 and Fig. 3). The conditions were identified from our earlier study on the regioselective SMCC reaction of 2,4-dibromopyridine with aryl boronic acids, which revealed the unusual C4 site-selectivity for Pd_3_Cl_2_1 and Pd(OAc)_2_/≤2PPh_3_ precatalyst systems.^10*b*^ As an aside, the same atypical C4 site selectivity is observed with immob-Pd_3_Cl_2_3 as catalyst, demonstrating the generalizability of immob-Pd_3_Cl_2_3 as a direct analogue for non-tethered Pd_3_Cl_2_1 in SMCC catalysis (Table S4†). In the present mechanistic study, however, we chose to focus on the simpler reaction of 4 with 5 to avoid possible regioselectivity complications. The base, *n*-Bu_4_NOH, is employed in a THF/H_2_O solvent mixture (1 : 1, v/v). While it is a highly basic, mixed solvent system, there is one single phase, where the organoboron species in solution is [ArB(OH)_3_]^−^[*n*-Bu_4_N]^+^. Where there is a question about Pd speciation, it is imperative to limit speciation in respect to boron, which makes the conditions advantageous for studying Pd catalyst speciation.

**Table 1:** Varying content of the palladium catalyst system in a standard SMCC reaction

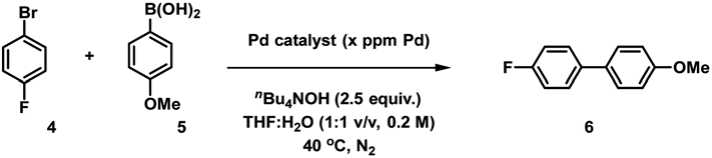					
Entry	Catalyst	Pd content (ppm)^a^	*T* (°C)	Time (min)	Conv.^b^ (%)
1	Supported Pd_3_Cl_2_	68	40	15	99
2	Supported Pd_3_Cl_2_	45	40	18	97
3	Supported Pd_3_Cl_2_	24	40	28	97
4	Pd_3_Cl_2_	68	23	5	95^c^
5	Pd_3_(OAc)_6_/6PPh_3_	68	40	4	82

a Pd ppm (moles) is three times catalyst ppm (moles) for the Pd_3_ cluster; the Pd concentration of 68 ppm (moles) equates to a 0.34 mol% catalyst loading with respect to 4 the [ArBr].

b Product 6 conversion (^19^F NMR; note product conversions mirrored in ^1^H NMR spectral data) of crude reaction sample with internal standards, 1,3,5-trimethoxybenzene and 4,4′-difluorobenzophenone.

c Conversion to 6 obtained at room temperature.

We selected to employ 68 ppm (by moles) of Pd (note: total ppm Pd is three times catalyst ppm; this Pd concentration equates to a 0.34 mol% catalyst loading with respect to 4). The SMCC reaction catalyzed by immob-Pd_3_Cl_2_3 reaches completion within 15 minutes to give cross-coupled product 6 (entry 1, Table 1). Lowering the Pd catalyst loadings to 0.22 mol% (immob-Pd_3_Cl_2_3, 45 ppm by moles Pd) and 0.12 mol% (immob-Pd_3_Cl_2_3, 24 ppm by moles Pd) led to extended reaction times (18 and 28 minutes, respectively) (entries 2 and 3, Table 1).

Remarkably, under comparable conditions using Pd_3_Cl_2_1, the reaction proceeded to completion within 5 minutes (entry 4, Table 1). We anticipated a higher rate of catalysis for Pd_3_Cl_2_1 since the accessibility to the “Pd_3_Cl_2_” core structure in immob-Pd_3_Cl_2_3 would be subject to greater substrate diffusion limitations. Nevertheless, the high rate of this reaction is remarkable, which supports the claims made about high reactivity using aryl cross-coupling partners (including aryl chlorides) made by Li and co-workers in their independent study.^4*d*^ By way of comparison, the “Pd(OAc)_2_/2PPh_3_” pre-catalyst system, known to invoke Pd_3_ cluster catalysis in SMCC and Kumada cross-coupling reactions,^10*b*^ also effectively catalyzed the reaction (entry 5, Table 1).

The SMCC reaction progress was monitored using ^1^H/^19^F NMR spectroscopic analysis, enabling kinetic profiles to be obtained. We examined both immob-Pd_3_Cl_2_3 and Pd_3_Cl_2_1, at different catalyst loadings. Interestingly, sigmoidal kinetic profiles were seen for reactions catalyzed by both immob-Pd_3_Cl_2_3 and Pd_3_Cl_2_1, suggesting that a pre-catalyst activation step is necessary. The induction period extends to slightly longer times on reducing the catalyst loading, which is longer for the immob-Pd_3_Cl_2_3 system than for Pd_3_Cl_2_1, presumably because of diffusion issues for the former catalyst. For the Pd_3_Cl_2_1 catalyst, one needs to go down to very low catalyst loadings (6.8 ppm Pd, 0.034 mol%) to reveal the full extent of the induction period.

**Fig. 4:**
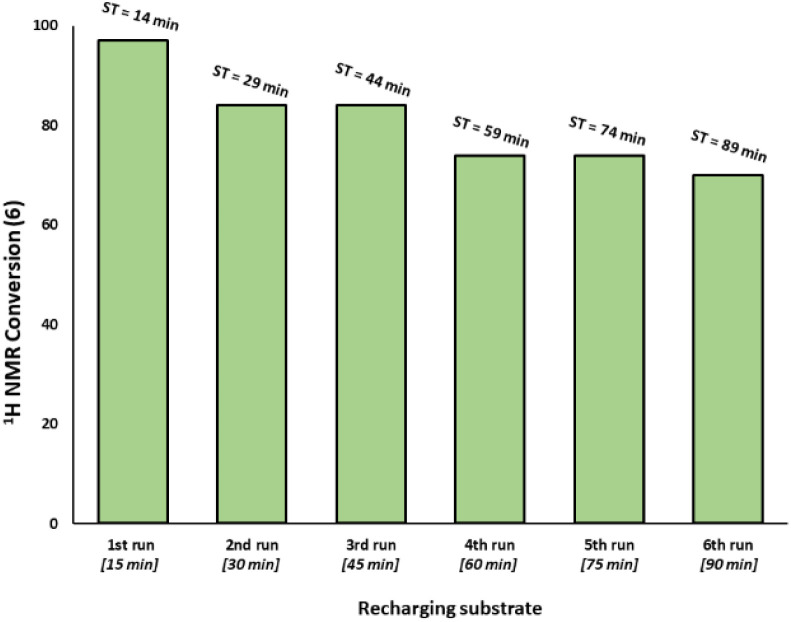
Recharging SMCC reaction promoted by immob-Pd_3_Cl_2_3 (1 mol%, 68 ppm Pd) with fresh batch of substrates and base at the end of catalysis (taken to be at 15 minutes intervals based on earlier reaction profiles in Fig. 3) and monitoring the reaction progress up to six runs (ST = sampling time during the overall experiment). The reaction was analyzed by ^1^H NMR using 1,3,5-trimethoxybenzene as internal standard.

Given the high catalytic activity associated with Pd_3_Cl_2_1 we hypothesized that the influence of the exogenous phosphine resin support 2 also merited assessment. The kinetic profiles in Fig. 3c for the reaction catalyzed by Pd_3_Cl_2_1: exogenous phosphine resin 2 in a 1 : 1, 1 : 2 and 1 : 3 ratio revealed that the activity is reduced *versus* Pd_3_Cl_2_1 alone and further reduced with increasing equivalents of resin (Fig. 3c). The model SMCC reaction was also recharged with both reactants and additives at *t* = 15 min (based on when the reaction ends as judged from the kinetic curve shown in Fig. 3) to assess catalyst reactivity and catalyst deactivation. ^1^H NMR analysis of samples taken at the end of each run revealed the temporal reactivity of 3 (six runs) with enduring good conversions to cross-coupled product (see Fig. 4). The result of this experiment suggests that the active catalyst can re-enter into the catalytic cycle in the presence of fresh reactants.

### Probing the heterogeneous behavior in the SMCC reactions

To assess whether the SMCC reaction occurs heterogeneously, or through a Pd leaching mechanism from the polymer resin, experiments were conducted to gauge the heterogeneity/homogeneity of the processes involving the immob-Pd_3_Cl_2_3 catalyst system. The mercury-drop test has been one of the common tools for examining heterogeneous catalyst behavior, where a positive test can result in poisoning of any aggregated Pd^0^ present (inferred as being the active catalyst species). The alternative outcome is that mercury has no effect, indicating a role for soluble molecular catalyst species (likely of low metal nuclearity).^15^ However, the applicability of the test for reactions involving different molecular complexes of Pd,^16^ Pt^17^ and Rh^18^ has been recently questioned, due to direct reactions of mercury with homogenous metal species such as palladacycles,^19^ or a high dependence of the test outcomes to the operational conditions (*e.g.* stirring rate/size of the reaction vessel).^20^ The validity of the mercury poisoning test for M^0^/NHC or M^II^/NHC (M = Pd, Pt) complexes was brought into question by Ananikov *et al.*^20,21^ Certain mononuclear Pd^II^L_*n*_ complexes react with metallic Hg through an oxidative-reductive transmetalation process to form Hg^II^L_*n*_ and Hg_*x*_Pd_*y*_ species, whereas Pd^0^L_*n*_ complexes decompose under exposure to metallic mercury.^20^ Thus, the issue is that a poisoned catalytic reaction system could have (potentially) involved mononuclear PdL_*n*_ complexes, bringing about an incorrect interpretation when poisoning does occur. Conversely, if a catalyst system is unperturbed by mercury addition, where the operational conditions are carefully selected to ensure the test can poison reactivity (see ESI†), the attribution of activity to aggregated Pd(0) may still be reasonably ruled out. That is, if the test is conducted correctly to ensure mercury contacts the entire system in adequate excess, an outcome of unperturbed reactivity in the presence of Hg is sufficient to exclude Pd(0) particles, which would suffer from Hg poisoning (while the converse test outcome is inconclusive for the reasons above).

**Fig. 5:**
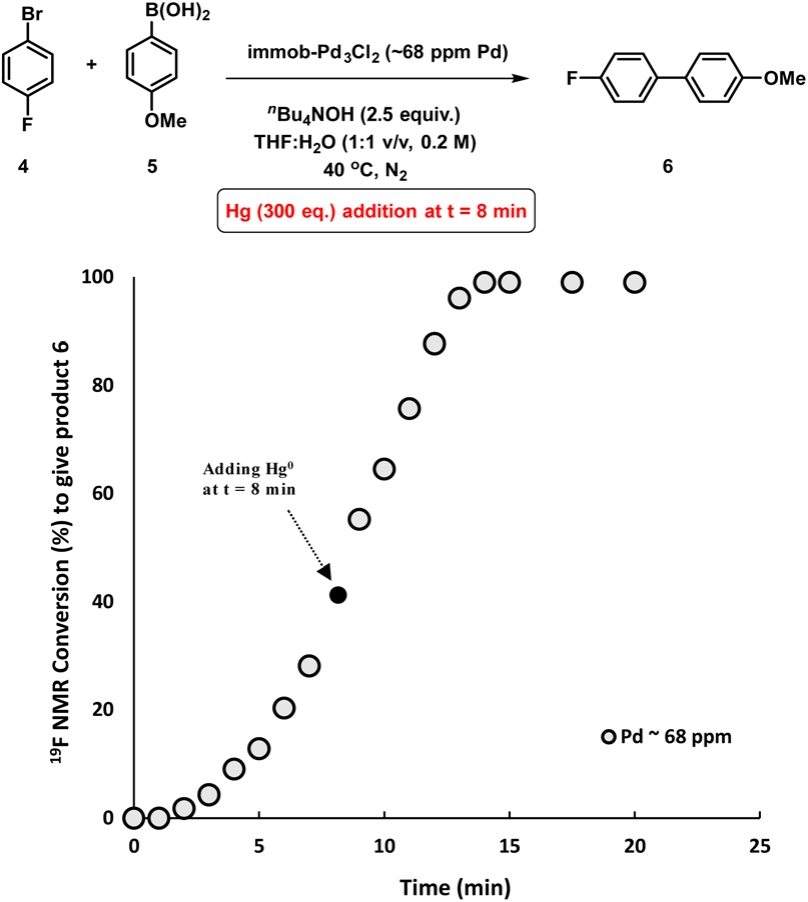
Kinetic profile of the model SMCC reaction obtained when excess metallic Hg (300 eq.) added at *t* = 8 min and reaction progress monitored by ^19^F NMR analysis. The Pd concentration of 68 ppm (moles) equates to a 0.34 mol% catalyst loading with respect to 4 [the ArBr].

With all these studies in mind, we conducted a mercury poisoning test for the model SMCC reaction catalyzed by immob-Pd_3_Cl_2_3 (68 ppm, 0.34 mol%), which reached 50% product conversion after 8 minutes (see Fig. 2a). Addition of metallic mercury (300 equiv. with fast agitation on a standard magnetic stirrer hot plate – see Section 2.8 in the ESI†) at *t* = 8 minutes had no effect on the reaction rate or product conversion, reaching completion within 15 minutes (Fig. 5). Interestingly, in a control reaction we determined that Pd_3_Cl_2_1 does react with metallic mercury, but only on longer timescales (see Fig. S21†), leading to decomposition. Our results, taken together show that: (a) Pd nanoparticles (Pd^0^ species) are not operative in SMCC reactions catalyzed by immob-Pd_3_Cl_2_3; (b) the Pd_3_Cl_2_ cluster motif is likely protected from metallic Hg in immob-Pd_3_Cl_2_3, under the reaction conditions tested.

In another experiment, the immob-Pd_3_Cl_2_3 catalyst was separated from the model SMCC reaction after 4 minutes to monitor the reaction progress. The analysis showed that the reaction proceeds although at a significantly slower rate (see Fig. S22†). While the result could suggest the leaching of soluble Pd^0^ species into the solution (note: not supported by the Hg drop test), some of the supported Pd catalyst particles evaded removal by filtration, even using syringe filters (with 0.22-micron pore size). Moreover, the presence of small particles were observed in the reaction flask post-filtration. Nevertheless, the removal of the majority of immob-Pd_3_Cl_2_3 showed reaction retardation. Such retardation (taken in isolation) on filtration of the solid species can't explicitly exclude the possibility of leached Pd nanoparticles or leached molecular species becoming bound to the filter, but the much-decreased rate on filtration (given the difficulty of excluding all the solid material) is consistent with the attribution of reactivity drop to the removed solid immob-Pd_3_Cl_2_3.

The three-phase test, devised by Rebek and Gavina,^22^ was used to test for potentially reactive soluble catalytic Pd species that detach from a heterogenous (pre)catalyst and leach into the solution (being the active catalyst species). This is typically done by immobilizing one of the reactants and probing its reaction with another soluble coupling partner, in the presence of the immobilized catalyst.^23^ The diffusion of two solids or solid-bound reaction components should be inhibited *versus* diffusion of soluble component(s). For this experiment, we prepared a polystyrene-bound aryl iodide 7. SMCC reactions of 7 with 5 were examined using either immob-Pd_3_Cl_2_3 or soluble Pd_3_Cl_2_1, under otherwise identical conditions (Table 2).

**Table 2:** SMCC on an immobilized aryl iodide substrate 7

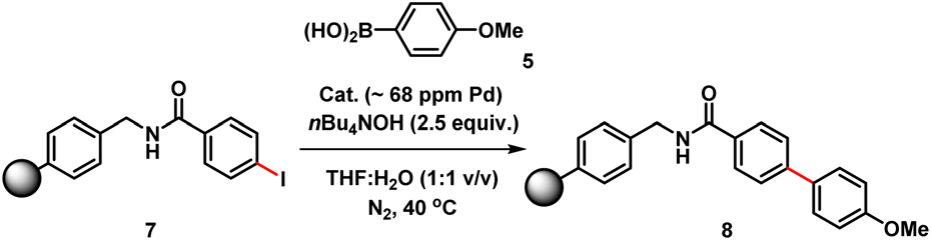			
Catalyst	Conversion^a^^,^^b^^,^^c^ to 8 (%)		
	1 hour	5 hours	24 hours
Pd_3_Cl_2_	1	8	28
Immob-Pd_3_Cl_2_	1	1	1

a Conversion monitored by ^1^H NMR (consumption of 5).

b <3% cross-coupled byproduct formed as result of amide hydrolysis.

c The Pd concentration of 68 ppm (moles) equates to a 0.34 mol% catalyst loading with respect to 7 [the ArI].

The immob-Pd_3_Cl_2_3 catalyst was effectively inactive in the three-phase test (less than 1% conversion to cross-coupled product 8 after 24 h). Immobilization of the aryl halide restricts the accessibility of the immob-Pd_3_Cl_2_3 catalyst; thus, any catalytic reaction cannot proceed under the employed reaction conditions. The outcome confirms that leached soluble Pd species are not playing a significant role under the reaction conditions tested. The equivalent reaction using Pd_3_Cl_2_1 as the catalyst was sluggish, as might be expected given the introduction of a diffusion constraint on one species involved, and the possibility the amide linker in immobilized derivative 7 might be considered inhibitory to the aryl halide (28% product conversion noted after 24 h).^24^ It should be noted that in an effort to keep the reactions more similar in timescale we have used the more reactive aryl iodide for these experiments (weak C–I bond, facile oxidative addition), which, while not perfect, removes the risk of speciation differences over longer timescales of reaction.

**Scheme 2:**
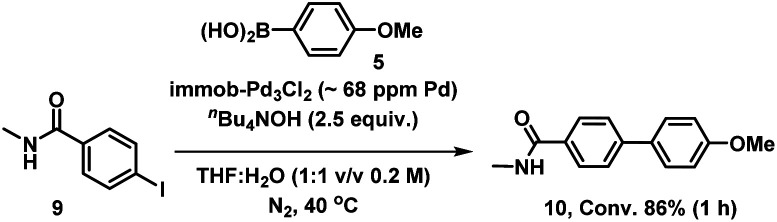
Control SMCC of 4-iodo-*N*-methylbenzeneamide 9 with 4-methoxyphenyl boronic acid 5, catalyzed by immob-Pd_3_Cl_2_3.

We therefore also conducted a control to determine that immob-Pd_3_Cl_2_3 was able to effectively cross-couple 4-iodo-*N*-methylbenzenamide 9 with *p*-methoxy-phenyl boronic acid 5 (gave 86% conversion to 10 after 1 h) (Scheme 2). It was important to confirm this, as it shows similar reactivity to that of our normal test reaction between 4 and 5, but with a similar inhibitory group/aryl iodide combination to that in immobilized derivative 7. Table 2 three-phase test experiment for the SMCC reaction of an immobilized aryl iodide 7 with 4-methoxyphenyl boronic acid 5 to give immobilized product 8.

**Fig. 6:**
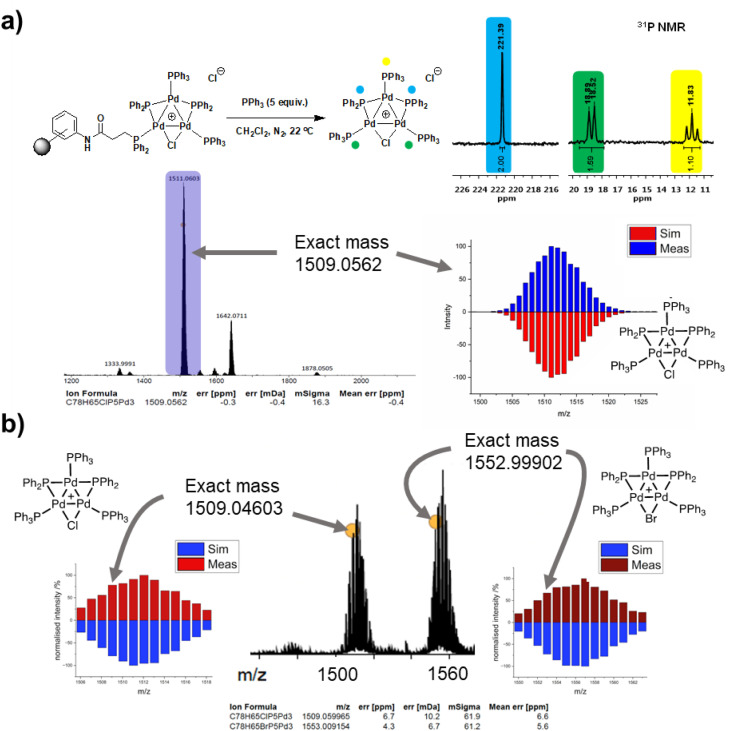
(a) Reaction of immob-Pd_3_Cl_2_3 with 5 equiv. PPh_3_ in CH_2_Cl_2_ at 22 °C; ^31^P NMR spectrum was recorded upon dissolution (within 10 min). ESI-MS (+ve mode) confirms pseudo-molecular ion at *m*/*z* 1509, with the correct isotopic distribution pattern centered at *m*/*z* 1511 (bottom right); (b) ESI-MS (+ve mode) analysis of the solution after reaction of excess PPh_3_ with post-reaction immob-Pd_3_Cl_2_3 cluster which was filtered off 10 minutes into Suzuki–Miyaura catalytic turnover and thoroughly washed, showing both [Pd_3_Cl]^+^ and [Pd_3_Br]^+^ ions shown, with their correct isotopic distribution patterns.

The three-phase test results cannot rule out the potential release of a “Pd_3_Cl_2_” species (or derivative) cluster from the support into the solution following a reaction with substrate(s), which could be subsequently redeposited on to the support. Supported Pd catalysts are known to exhibit release-redeposition (catch) mechanisms in catalysis.^25^ There is no evidence for loss of the “Pd_3_Cl_2_” species from immob-Pd_3_Cl_2_3. Indeed, the only way to trigger release is by the reaction of exogenous (excess) PPh_3_ with immob-Pd_3_Cl_2_3 in CH_2_Cl_2_, which occurs instantaneously at room temperature to deliver Pd_3_Cl_2_1 into solution; confirmed by ^31^P NMR and ESI-MS (+ve mode) (Fig. 6a).

This ability to trigger release of the Pd_3_ cluster from the resin support affords an additional test as to whether the Pd_3_ cluster remains intact on the resin during reaction. An SMCC reaction of 4-fluoro-bromobenzene 4 and 4-methoxyphenyl boronic acid 5 was again performed under the normal reaction conditions with 68 ppm Pd, but stopped after 10 minutes by filtration (where Fig. 3 shows ∼60% conversion, but catalytically relevant species are still present). The filtered resin was then washed thoroughly and reacted with excess PPh_3_ to trigger release of the cluster into clean solvent (details given in the ESI†). ESI-MS of the released Pd_3_ shows not only the initial [Pd_3_Cl]^+^ ion, but also [Pd_3_Br]^+^ (Fig. 6b), pointing to the resin bound species having been involved in a process that swaps out the bridging halide.

The results taken together suggest that the induction period observed from the cross-coupling catalysis is not a consequence of leached mononuclear or nanoparticulate Pd catalyst formation. At 10 minutes where >50% conversion has occurred ICP showed only leaching of 0.58% of the initial Pd into the liquid phase for a sample filtered to remove the solid (and some of the polymer bound material undoubtedly evades the filter, so this is a worst-case scenario). This is further corroborated by the quantification of the XAS step-edge height for the pre-reaction immob-Pd_3_Cl_2_3 and ‘during reaction’ samples—proportional to concentration—implying the solid contained the same Pd amount, within error, in both cases. In addition to these tests performed to exclude catalysis by leached molecular or nanoparticle species, the overall rates of reaction imply a minor component would have to exhibit far greater performance than those seen for SMCC reactions at these temperatures ∼40 °C (see Table S11;† it should be noted that typical rates of “ppb Pd” SMCCs or catalysis by “impurity Pd” are generally identified only at significantly higher temperatures, *e.g.* 110 °C),^26*a*^ in keeping with relating Pd-catalyzed cross-coupling reactions where ppm-Pd descriptors have been rigorously examined.^26*b*^

A possible explanation for the induction period is the interplay of planar aromatic [Pd_3_X]^+^ species with structurally distorted non-aromatic [Pd_3_X/X'] species, both of which are plausible species characterized by single crystal XRD methods.^10*a*^ The possible importance of d-orbital aromatic stability metal aromaticity^27^ in rendering, for example the [Pd_3_(μ-SR)_3_(PR_3_)_3_]^+^Y^−^ cluster complexes as being oxygen and moisture-stable,^28^ has been previously highlighted,^29^ and its potential relevance to Pd_3_ cross-coupling discussed.^30^ Here the postulated change from more stable aromatic Pd_3_Cl_2_ to the structural-distorted bromide bridged cluster being accompanied by an induction and rate increase is consistent with this being an important effect in these systems. The primary point, however, is that the immob-Pd_3_Cl_2_3 is a viable tool to study the component mediating Pd_*n*_ cluster catalysis.

### Studies by X-ray absorption spectroscopy (XAS: EXAFS/XANES)

XAS has been used widely to study the physical form of Pd catalysts,^31^ particularly immobilized Pd catalyst systems^25*b*,32^ and well-defined and/or evolving Pd nanoparticle species.^33^ The pros of the approach are: (1) X-ray absorption is unambiguously specific to a given element, in this case Pd; (2) the penetrating nature of X-rays makes it possible to obtain measurements in complex matrices without perturbing the system, here we do not remove from the solvent for analysis; (3) the spectral information obtained is rich, with information about oxidation state, co-ordination environment and geometries being possible to obtain with appropriate modelling; and (4) it is inherently quantitative with normalized spectra of different species allowing their relative contributions to a mixture to be ascribed. The cons of the XAS approach are: (1) it is an averaging method so small components can be overlooked (but this is true of many techniques); and (2) high intensity beam damage to the Pd samples is possible (reassuringly in the present case excellent agreement is seen for the as prepared immob-Pd_3_Cl_2_3; fitting models are based on expected structure). The reference standards selected in our study include combinations of bromide, phosphines and *n*Bu_4_N^+^ species, the latter from the base used in reaction, and these have largely not been reported previously. PdBr_2_ is consistent with spectra reported in the literature.^34^

**Fig. 7:**
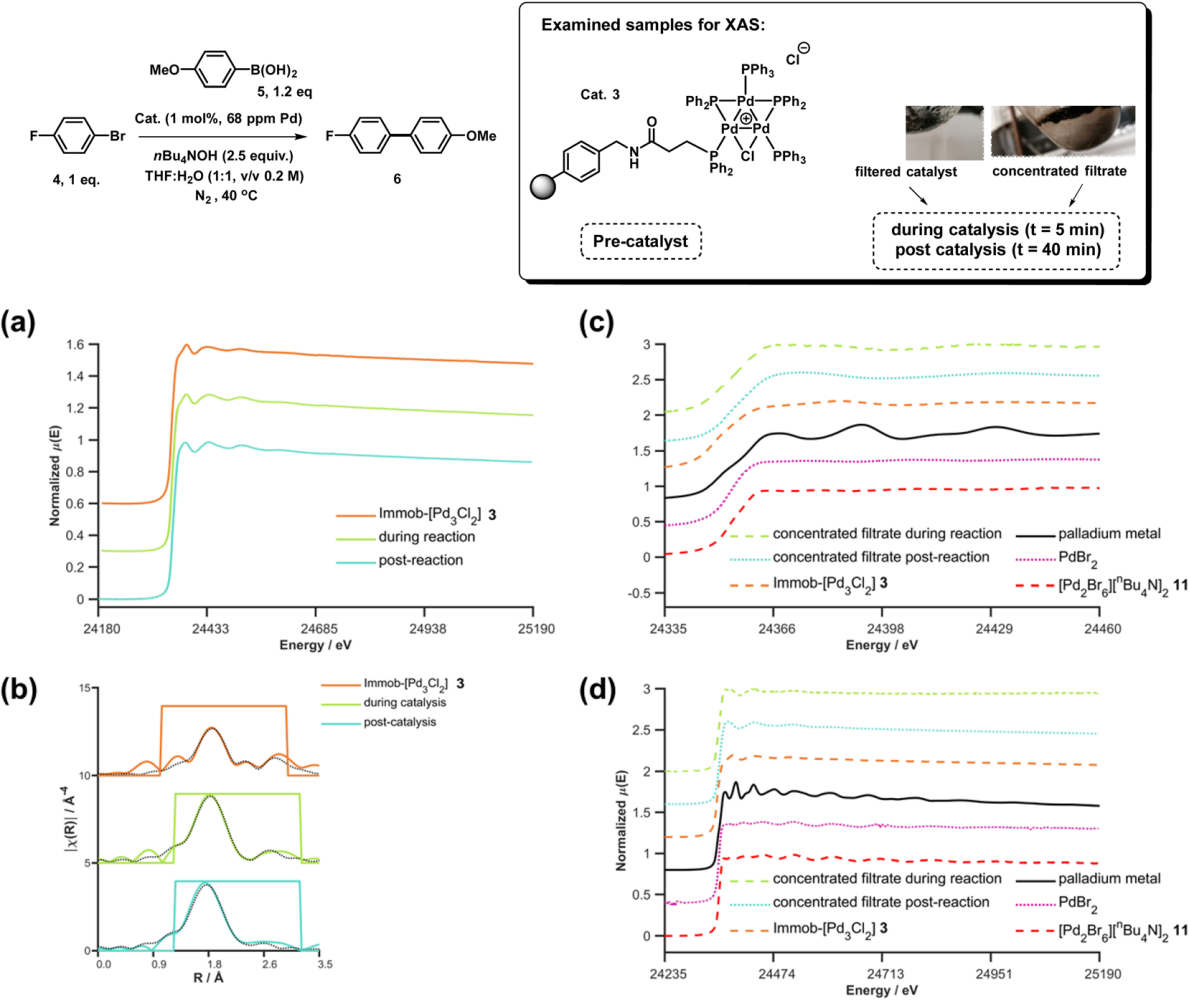
Pd K-edge XANES spectra of the immobilized-[Pd_3_Cl_2_] cluster 3 as reference (orange), during reaction (green, reaction stopped at 5 min) and post-reaction (cyan) (a); Pd K-edge *R*-space EXAFS data and fits of the immobilized-[Pd_3_Cl_2_] cluster 3 before reaction, during reaction and post-reaction, solid colored lines denote the data, dotted black lines the fits, with the fitting window indicated in grey (full details given in the ESI†) (b); Pd K-edge XAS spectra (XANES (c) and extended (d)) of the concentrated filtrate during reaction and post-reaction, compared to immobilized-[Pd_3_Cl_2_] 3, and PdBr_2_ and [Pd_2_Br_6_][^*n*^Bu_4_N]_2_ as references compounds. Spectra offset for clarity, the lowest spectrum correctly placed on the *y*-axis in each case, with XANES spectra all normalized between 0 and 1 and *R*-space EXAFS data offset by 5 units per spectrum.

To explore the identity of the catalytic Pd species during the SMCC reactions, and to identify the fate of the catalyst after reaction completion, samples of reactions during and after catalysis were analyzed using Pd K-edge extended X-ray absorption fine structure (EXAFS) and X-ray absorption near edge structure (XANES) techniques. For preparation of the samples, the catalyst particles were separated from the reaction mixture by cannula filtration (paper filter), after 5 minutes (during productive catalyst turnover, *i.e.* catalyst activated) and also after reaction completed. We further examined the concentrated filtrate of the reaction solution after separation of the supported catalyst system, which was evaporated to dryness both after 5 minutes and after reaction completion (Fig. 7). Comparison of these samples allows us to understand whether Pd_3_Cl_2_ clusters remained intact and potentially give information about any leached Pd species present.

The Pd K-edge XAS spectra of the filtered catalyst, both during and after reaction in both the XANES region (Fig. 7a) and based on fits of the EXAFS data to the Pd_3_ motif (Fig. 7b), are in excellent agreement with the data obtained for immob-Pd_3_Cl_2_3 before reaction (fitting parameters and details are given in the ESI†). The invariant XANES suggest no marked change in either oxidation state or local co-ordination environment, and the fact that the Pd_3_ motif (with Pd, P and halogen nearest neighbors) is preserved well in the fitting of the EXAFS data strongly points to the fact that the structure of solid-supported Pd_3_ clusters remains similar during the catalysis and even after catalysis. As was highlighted in Fig. 1, this situation afforded by our immobilization strategy is an unusual and valuable occurrence in mechanistic studies of Pd cross-coupling catalysts, where deactivated Pd species are typically all that remains upon post-mortem analysis. The structure of immob-Pd_3_Cl_2_3 therefore remains a realistic model for the species bound to the polymer throughout the reaction. Note: XAS and the use of nearest neighbor fits cannot strictly distinguish species 1 and 3, but the difference in reactivity between them, three-phase test and fact that this species is extracted for analysis by filtration of the polymer all imply this is likely to be 3 rather than 1.

**Scheme 3:**
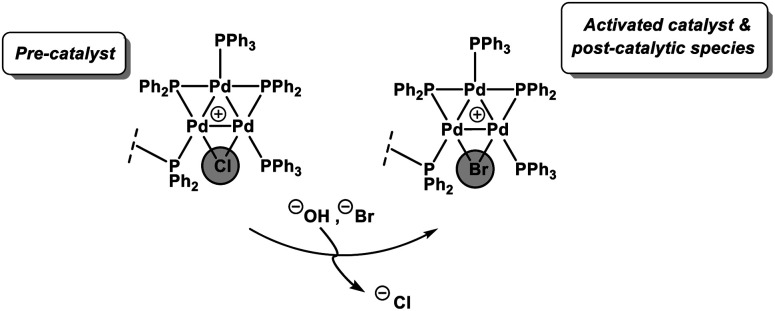
The halide exchange of bridging chloride by bromide sourcing from the substrate (in immob-Pd_3_Cl_2_3). The best fit of the EXAFS spectra being obtained for Br-bridged Pd_3_ cluster indicates that the exchange probably occurred during catalyst activation and remained intact during catalyst deactivation in the model SMCC reaction. Note that the EXAFS analysis cannot preclude formation of non-aromatic Pd_3_Br_2_ or Pd_3_BrOH species.

Another significant finding from EXAFS analysis was revealed by comparing the goodness of fit for bridging –Br, –Cl and –Ar (a carbon atom in the 1st co-ordination shell, which scatters less than Cl) for each sample. As expected in the pre-reaction catalyst, Cl provides a suitable fit. For the post-reaction samples placing Br in the bridging position between two palladiums (average co-ordination number of Pd by Br = 0.67) provides a distinctly better fit, and in neither the during reaction or post-reaction sample will placing a carbon in the bridging position, representing a bridging aryl, provide an acceptable fit (see ESI†). The better fit for the bromide-bridged Pd_3_ cluster suggested a substitution of chloride by bromide in the structure of the Pd_3_ cluster. This is supported by the reaction clusters released from the resin post-filtration after 10 minutes of reaction in Fig. 6b being a mixture of Cl and Br bridged Pd_3_ clusters (also seen by Li *et al.* using ESI-MS with both Br and I containing aryl halide reactants).^4*d*^ The precise steps by which the bromide-containing Pd_3_ cluster forms likely involve reactions with substrates (depicted in Scheme 3). Repetition of the reaction kinetic profile for immob-Pd_3_Cl_2_3, but in the presence of a large excess of Br^−^ (1 equiv. ^*n*^Bu_4_NBr with respect to aryl halide) showed no significant change in kinetics, suggesting this incorporation must occur reactively (see ESI†).

Plots of the filtrate samples (where polymer resin containing immob-Pd_3_Cl_2_3 was filtered out and the solvent removed to dryness) obtained during and after catalysis against a variety of reference samples in both XANES (Fig. 7c) and EXAFS (Fig. 7d) regions show no match to the spectral features for palladium metal, Pd_3_ clusters, nor other reference samples, including PdBr_2_ and [Pd_2_Br_6_][*n*-Bu_4_N]_2_11. It should be noted at this point that only very subtle differences are seen experimentally (Pd_7nm_–Pd_foil_)^35^ or predicted theoretically as a function of metallic palladium nanoparticle size (Pd_13-atom_–Pd_foil_),^36^ and so the Pd foil standard is a suitable reference for metallic Pd particles of any size. Attempts to use mass spectrometry to identify the Pd species in the filtrate were unsuccessful, either due to other species present swamping the signal or there being a poorly defined mixture of end product species. The nature of this species is discussed later, but at this point we can confidently rule out this leached Pd being present as Pd_3_ clusters, which would readily be seen by HRMS, metallic palladium particles (readily seen in XAS) or the other reference samples considered.

**Fig. 8:**
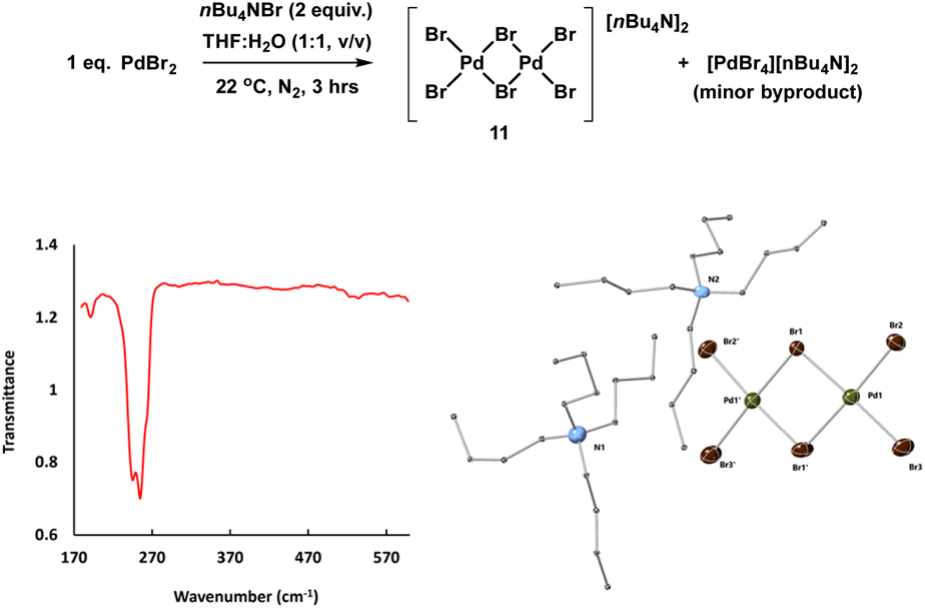
Synthesis and key characterization for 11: far IR spectrum (left) showing characteristic bridged Pd–Br stretch bands at 244.9 and 254.6 cm^−1^ and X-ray single crystal structure of [Pd_2_Br_6_][*n*Bu_4_N]_2_11 (right), thermal ellipsoids set at 30% probability and hydrogen atoms removed from both structures for clarity. Selected bond distances for 11 (Å): Pd_1_–Br_1_ = 2.445(2), Pd_1_–Pd_2_ = 2.407(2), selected bond angles (°): Br_1_–Pd_1_–Br_2_: 91.114(8), 
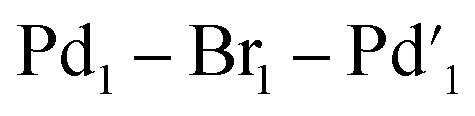
: 93.547(7), Br_2_–Pd_1_–Br_3_: 91.893(9).

The reference sample [Pd_2_Br_6_][*n*-Bu_4_N]_2_11 is reported to be catalytically active in cross-coupling reactions and we were therefore careful to consider its possible formation from the Pd_3_X_2_ catalyst system.^25*b*,37^ Here, complex 11 was prepared through treatment of *n*-Bu_4_NBr and PdBr_2_ in THF and H_2_O (1 : 1) under inert atmosphere at room temperature and was characterized by far infrared, elemental analysis and X-ray single crystal diffraction (Fig. 8).

The pre-catalyst 11 showed very high reactivity in forming cross-coupled product 6. The result of this experiment supports the XAS outcome that leached Pd species (which were relatively inactive) do not share any similarity with 11. It is also notable that even if such species were present at low concentration (below those that we could detect), they would not be capable of promoting the cross-coupling reaction at this temperature at the rate seen by the present highly reactive immob-Pd_3_Cl_2_3 catalytic system.

**Scheme 4:**
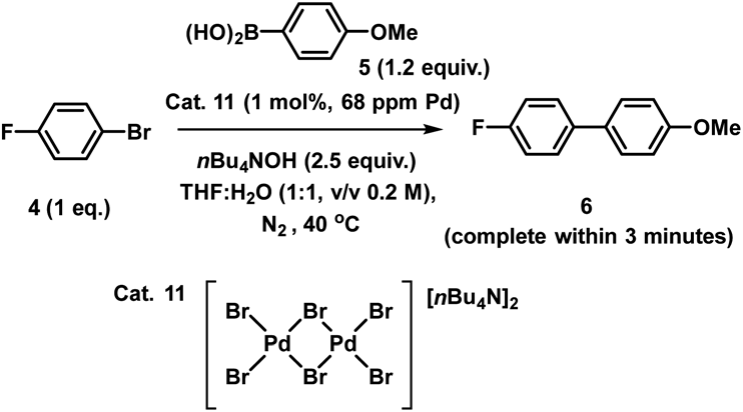
Model SMCC reaction of 4 and aryl 5 under the optimized reaction conditions, using [Pd_2_Br_6_][*n*Bu_4_N]_2_11 (0.1 mol%, 68 ppm Pd) as catalyst, giving complete conversion to 6 within 3 minutes. The crude reaction mixture was analyzed by both ^1^H and ^19^F NMR analysis using 1,3,5-trimethoxybenzene and 4,4′-difluorobenzophenone as internal standards.

The catalytic activity of 11 was examined in the model SMCC reaction of 4 and 5 (using 0.1 mol%, 68 ppm Pd), under our working reaction conditions, showing quantitative conversion to 6 within 3 minutes (Scheme 4), *cf.* 15 minutes for immob-Pd_3_Cl_2_3 (Table 1).

#### XAS analysis of Pd_3_Cl_2_ under SMCC reaction conditions

**Fig. 9:**
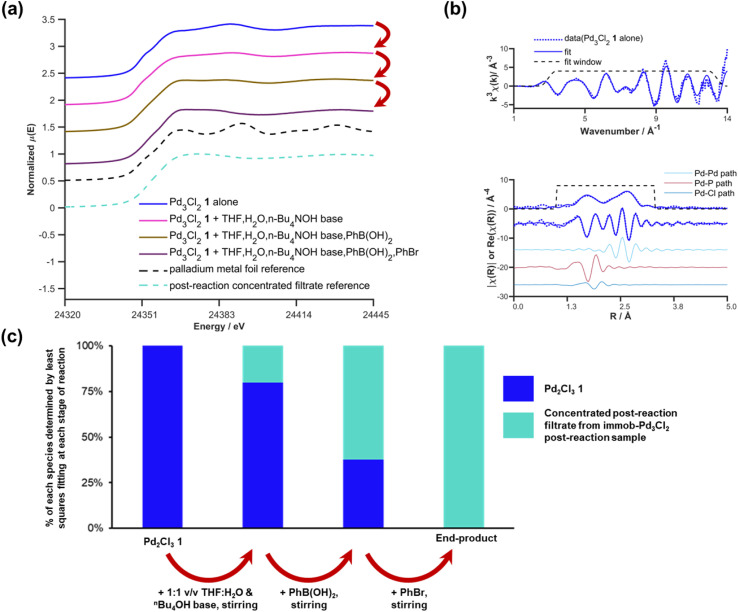
Pd K-edge XANES spectra of the Pd_3_Cl_2_ cluster 1 alone (blue), and after stepwise addition of H_2_O/THF and *n*Bu_4_NOH base (pink), phenylboronic acid (brown), and phenyl bromide (platinate purple) (a); Pd K-edge *k*-space and *R*-space EXAFS data and fits of the initial Pd_3_Cl_2_ cluster 1, dashed blue lines denote the data, solid blue lines show the fit, with the fitting window shown above and the contributing paths from Pd–Pd, Pd–P and Pd–Cl shown below (b); plot of the contributions determined by least squares fitting the spectra shown in (a) for the stepwise reaction of the initial Pd_3_Cl_2_ cluster 1 and the concentrated post reaction filtrate from the immob-Pd_3_Cl_2_ experiments as explained in the text (c). Spectra offset for clarity, the lowest XANES spectrum is correctly placed on the *y*-axis with all XANES spectra normalized between 0 and 1 and EXAFS oscillations centered around *y* = 0 prior to any offset.

To provide further insight into the changes to the catalyst occurring, an analogous XAS study of the homogeneous Pd_3_Cl_2_1 undergoing the SMCC reaction under the same conditions was conducted. Unlike immob-Pd_3_Cl_2_3, the homogeneous Pd_3_Cl_2_1 cannot be separated from any leached or reaction products by simple filtration (*i.e.* without risking significant perturbation of the catalyst species). Instead, at each stage of the reaction (Pd_3_Cl_2_1 alone; Pd_3_Cl_2_1 in THF/H_2_O 1 : 1 v/v solvent after *n*-Bu_4_NOH base added; after aryl boronic acid added; after aryl bromide added) an aliquot of the reaction mixture containing all components was rapidly quenched to LN_2_ temperatures in a Nalgene cryovial for XAS analysis. The resulting sequence of XANES spectra are shown in Fig. 9a. The initial spectrum of Pd_3_Cl_2_1 is an excellent fit to the expected first shell co-ordination of Pd in the cluster (Fig. 9b).

However, it is immediately clear from the XANES that even combining with THF/H_2_O/base begins to alter the Pd speciation. Further time and/or reagent addition caused further changes in the spectra (it is difficult to discriminate which, as both time and addition of reagents took place). The changes in the spectra mirror the visual observation that there was an immediate change to a black color upon adding the aqueous *n*-Bu_4_NOH base to a THF solution of Pd_3_Cl_2_1. While our first thoughts turned to precipitation of Pd black, no distinct large Pd particles were visible by eye from this point throughout the subsequent reagent addition steps performed (during which the appearance of the reaction solution remained unchanged). The impression of there being a single liquid phase can be misleading if the Pd is nanoparticulate in nature and does not precipitate. However, as will be seen shortly the conclusion that no Pd metal is being formed (nanoparticulate or otherwise) is supported by further analysis of the XAS data.

Qualitatively the XANES spectra through the reaction steps appear to become progressively more similar to the filtrate during reaction from the immob-Pd_3_Cl_2_3, while bearing little similarity to Pd metal (spectra both shown for reference in Fig. 9a). It is important to note that the above work on immob-Pd_3_Cl_2_3 showed that the leached species seen in the concentrated post-reaction filtrate were inactive for the test SMCC reaction *versus* either Pd_3_Cl_2_1 or immob-Pd_3_Cl_2_3.

A more quantitative analysis of the XANES data in Fig. 9a and *k*-space EXAFS data can be achieved with principal component analysis (PCA) and least squares fitting. PCA of the XANES series in Fig. 9a shows significance above the noise in the first two components (Fig. S34,† the edge region is slightly present in the third component, but noise at the edge where a large change is present leads to artificial variation because of slight energy misalignments). A scree plot (Fig. S34b†) also has an elbow after the first two points, pointing to only two principal components that contribute significantly. Similar analysis using an approach used elsewhere^34^ on the *k*-space EXAFS spectrum region between *k* = 0 and *k* = 11.5 is again largely comprised of only two components that are significantly above the noise level of the data (Fig. S36†). It should be noted that the sample after adding base, but no other reagents, is slightly less well fitted in the EXAFS region by only two components, which may hint at transitional species being present, but there is insufficient data to infer more than this being a possibility.

Given the visual similarity between the post-reaction filtrate sample (leached from immob-Pd_3_Cl_2_3) to the later stages of the reaction of Pd_3_Cl_2_1, a two-component linear combination fit was undertaken for each spectrum in the sequence in Fig. 9a. This produced a good fit in the XANES region of the data (Fig. S40†), with the corresponding quantities of Pd_3_Cl_2_1 and the filtrate-like species being shown in Fig. 9c. This pleasingly shows a similarity between the chemical fate of the homogenous and albeit more stable immobilized Pd_3_Cl_2_ catalysts, supporting the idea that the immobilized system can facilitate broader studies of Pd_3_Cl_2_1. Given the above inactivity of the leached species in the filtrate that appears over time and the fast kinetics of the reaction, we have no evidence for other species besides Pd_3_Cl_2_1 being involved in the reaction process.

While the precise chemical identity of the species from the filtrate is somewhat unimportant if the species is ascribed as inactive, understanding the fate of Pd_3_Cl_2_1 is still of interest to understand the reactions that bring about its formation. As noted earlier the XANES spectra of the species in the filtrate did not fit visually with spectra of any common standards, such as PdBr_2_, Pd metal, or [Pd_2_Br_6_]^2−^. While a visual similarity in the spectra is a good first check, in the ESI† we consider a more quantitative approach using principal component analysis, which has been employed in XANES studies for some time (Section S2.16†).^38^ This speculatively points to some form of Pd_*x*_(PPh_3_)_*y*_-type species being the end fate of Pd in this system.

Finally, the stepwise approach used in this experiment allows us to re-examine the mechanistically surprising claim of Li and co-workers that an aryl-bridge Pd_3_ cluster forms as a stable transmetallation intermediate that can be observed by XAS.^4*d*^ The details of the experiments conducted previously are unclear, but the concentrations used in transmission XAS appear to have been around 0.01 wt% Pd from the details given in their publication, lower than is typically suitable for transmission experiments and likely produce noisy data. The claim concerning transmetallation is on the basis of reduced amplitude at low values of *k* (<5 Å), but this is extremely susceptible to the background functions and the energy spectra from which the *k*-space data were obtained were not given. The *R*-space data presented by Li and coworkers seem distinctly different from those we have consistently obtained for the cluster (homogenous or immobilized) with weaker than expected amplitudes for the Pd–Pd scatter (which dominates the region 2.5–3 Å), and an absence of the Pd–Cl scatter contribution (easier to identify when fitting the real part of the data than by inspection of the magnitude *R*-space plots). Repeating the stepwise experiment with the 1 : 1 v/v 2-propanol/H_2_O solvent mixture they report and using K_2_CO_3_ base as they did (which is only moderately soluble, although can form methoxide with alcohols^39^) we were able to see the Pd_3_Cl_2_1 cluster intact at each step of the process, with only a halogen exchange on adding bromobenzene (Fig. S36–S40†). Crucially in the sample after all other reagents were added except bromobenzene the spectrum is a significantly better fit to chloride than carbon (aryl) in the bridging position. In short, obtaining higher quality data at the low concentrations of Pd typically used in SMCC reactions by taking advantage of modern fluorescence detection capabilities (B18 Diamond Light Source) and fitting this using a summation of theoretically calculated scattering paths (ARTEMIS^40^) we saw no evidence with either the immobilized cluster or attempts to reproduce their experiment using Pd_3_Cl_2_1 for the proposed [Pd_3_Ar]^+^ intermediate. The other evidence they presented for this was based on ESI-MS of CH_2_Cl_2_ solutions of the reagents.^4*d*^ In our hands we found that the base (K_2_CO_3_) is insoluble in this solvent/it is not the medium in which the reactions were conducted (note: limited experimental details given in the original publication). It also appears the signal associated with [Pd_3_Ar]^+^ is still <20% of the Pd_3_Cl_2_1 cluster signal, and so even if present in the same amount, XAS being an average technique the Cl bridge would still yield a better fit (and have dominated their data also). It is also noteworthy that using some routes to prepare the Pd_3_Cl_2_1 cluster, we have sometimes seen the aryl bridged Pd_3_ cluster as a synthetic biproduct (rather than reactive intermediate – Fig. S27†), which is a possible alternative origin for such species. We have repeated the synthetic route to 1 described by Li and co-workers (yield = 2%) and have not been able to obtain evidence showing the intermediacy of [Pd_3_Ar]^+^. One final point is a further potential for confusion in their study arises from the fact that the phenyl group in the PhB(OH)_2_ substrate used is the same as that found in the PPh_3_/PPh_2_ ligands in Pd_3_Cl_2_1, avoided in the present study through the use of the methoxy-functionalized boronic acid 5. In short, the current study does not provide any evidence to support the existence of the aryl-bridged intermediate suggested by Li and co-workers.^4*d*^ However, additional work is required to fully investigate this hypothesis and establish if such a species is kinetically relevant, using a series of organoboron compounds to probe the transmetalation step first hypothesis.

## Conclusions

In conclusion, we studied ‘Pd_3_Cl_2_’ catalytic behavior in a typical SMCC reaction by tethering it to a resin support to afford a valuable tool for mechanistic studies. Experimental heterogeneity tests, such as Hg drop test and three-phase test, showed no active Pd leached from the immobilized Pd catalyst within the reaction's time scale. Analysis of XAS data from the filtered catalyst during and after reaction showed the triangular Pd_3_ cluster motif intact, with only exchange of chloride for bromide in the bridging position during the substrate turnover. XAS of the filtrate after catalyst removal showed non-active Pd species, with no match to a series of reference samples such as PdBr_2_, [Pd_2_Br_6_][*n*-Bu_4_N]_2_, and Pd foil. XAS analysis of non-tethered (non-mobilized) Pd_3_Cl_2_1 catalyst in the same SMCC reaction, conducted stepwise, indicates the Pd speciation at the later stages of the reaction is like that in the reaction filtrate when immob-Pd_3_Cl_2_3 was used as catalyst. Using principal component analysis and target transformation from a range of potential standards along with the available EXAFS data, the identity of the inactive Pd species in the filtrate sample from reaction of immob-Pd_3_Cl_2_ or later stage reaction of Pd_3_Cl_2_ was suggested to be Pd_*x*_(PPh_3_)_*y*_, exhibiting a blend of Pd–Pd and Pd–P interactions. This study provides the first experimentally compelling insight^30^ into the speciation of Pd involving the catalytic behavior of Pd_3_ type clusters stabilized by phosphorus containing ligands within cross-coupling reactions. Finally, during the proof stage for our manuscript we became aware of a study reported by Scott *et al.* involving the temperature-inducated activation of the Coulson-cluster on a carbon support. The results are complementary to those described here, and provide insight into how changes in the ‘Pd_3_Cl_2_’ cluster can occur at significantly higher temperatures (between 150 and 250 °C).^41^

## Abbreviations

EXAFS: Extended X-ray absorption fine structure
LN_2_: Liquid N_2_
PCA: Principal component analysis
SMCC: Suzuki–Miyaura cross-coupling
THF: Tetrahydrofuran
XANES: X-ray absorption near edge structure
XAS: X-ray absorption spectroscopy

## Data availability

A dataset containing raw experimental data has been deposited to Research Data York, which can be accessed through the following link (https://pure.york.ac.uk/portal/en/datasets/evidence-for-suzukimiyaura-cross-couplings-catalyzed-by-ligated-p).

## Author contributions

I. J. S. F. conceived the project and plans, with input from N. J., concerning the design, synthesis and application of the immobilization Pd_3_ cluster species. I. J. S. F. secured the research funding to support N. J. and N. W. J. S., S. K. B. led the synchrotron research work; S. K. B. and I. J. S. F. proposed experiments for and secured access to diamond facilities. S. K. B. assisted with the interpretation of catalytic data and design of appropriate control experiments, with input from N. J., N. W. J. S. and I. J. S. F., T. T. carried out the single crystal X-ray diffraction analysis. N. J., N. W. J. S., S. K. B. and I. J. S. F. executed the majority of synchrotron experiments at diamond (supported by diamond/UK Catalysis Hub teams – see acknowledgements). N. J. and S. K. B. conducted the XANES/EXAFS analysis, the interpretation of which was supported by discussion with I. J. S. F. N. J. wrote an initial draft manuscript, with input from all authors. The final version of the paper was written jointly between I. J. S. F. and S. K. B., with contributions and confirmation provided by all co-authors. Much of the synthetic and catalytic work was conducted by N. J. which forms an integral part of her PhD thesis.

## Conflicts of interest

There are no conflicts to declare.

## Supplementary Material

SC-015-D3SC06447F-s001

SC-015-D3SC06447F-s002
