# Supplementary material for: Evidence for Suzuki–Miyaura cross-couplings catalyzed by ligated Pd3-clusters: from cradle to grave
Source: Chem Sci. 2024 Jan 15;15(8):2763–77. doi: 10.1039/d3sc06447f (PMC10882490; doi:10.1039/d3sc06447f)
Supplement: SC-015-D3SC06447F-s001 [file SC-015-D3SC06447F-s001.pdf]

Electronic Supplementary Material (ESI) for Chemical Science.
This journal is © The Royal Society of Chemistry 2024

Supporting Information

Evidence for Suzuki-Miyaura Cross-Couplings catalyzed by ligated Pd_3–Clusters: from cradle to grave

Neda Jeddi,[a] Neil W. J. Scott,[a] Theo Tanner,[a] Simon K. Beaumont[b,] and Ian J. S. Fairlamb[a,]**

[a] Department of Chemistry, University of York, York, YO10 5DD, UK. [b] Department of Chemistry, Durham University, South Road, Durham, DH1 3LE, UK.

Contents

1 General Information 4

1.1 General Considerations: 4

1.2 Laboratorial analysis 5

1.3 Instrumentation 5

2 Experimental Details 7

2.1 Synthesis Procedure for $[Pd_3(\mu\text{-}Cl)(\mu\text{-}PPh_2)_2(PPh_3)_3][Cl]$ "Pd_3Cl_2" Cluster[1] 7

2.1.1 Counterion Exchange of $[Pd_3(\mu\text{-}Cl)(\mu\text{-}PPh_2)_2(PPh_3)_3][Cl]$ and Formation of $[Pd_3(\mu\text{-}Cl)(\mu\text{-}PPh_2)_2(PPh_3)_3][SbF_6]$ (reference compound) 8

2.2 Synthesis procedure for functionalized polystyrene resin 8

2.3 Synthesis procedure for immobilization of $[Pd_3(\mu\text{-}Cl)(\mu\text{-}PPh_2)_2(PPh_3)_3][Cl]$ 10

2.4 General procedure for immobilized $[Pd_3(\mu\text{-}Cl)(\mu\text{-}PPh_2)_2(PPh_3)_3][Cl]$-catalyzed Suzuki-Miyaura cross-coupling reaction of 4-bromo-1-fluorobenzene and 4-methoxy-phenylboronic acid 13

2.5 Calculation of palladium wt.% within immobilized Pd_3Cl_2 cluster using elemental

analysis along with subsequent ppm and mol% calculation of palladium in Suzuki-Miyaura Cross-coupling Reaction 14

2.6 Kinetic assay analysing the effect of different quantities of Pd catalysts used in the standard Suzuki-Miyaura cross-coupling reaction 15

2.6.1 Kinetic analysis of $Pd_3(OAc)_6/2PPh_3$ and $Pd_3(OAc)_6/3PPh_3$ as catalyst in standard SMCC reaction (68 ppm Pd) 16

2.6.2 Kinetic analysis of individual immobilized Pd_3Cl_2 as catalyst (24-68 ppm Pd) used in standard SMCC reaction 17

2.6.3 Kinetic analysis of individual Pd_3Cl_2 as catalyst (6.8-68 ppm Pd) used in the standard SMCC reaction 19

2.6.4 Kinetic analysis of individual Pd_3Cl_2:resin as catalyst (68 ppm Pd) in different ratios used in the standard SMCC reaction 22

2.6.5 Kinetic analysis of Pd_3Cl_2 as catalyst (24 ppm Pd) with *n*Bu_4NBr (1 equiv.) ... 23

2.7 Procedure for recharging substrates and reagents in the standard SMCC reaction .. 24

2.8 Hg poisoning test kinetic assay for immobilized Pd_3Cl_2-catalyzed standard Suzuki-Miyaura cross-coupling reaction 25

2.8.1 Procedure for reaction of Hg(0) with Pd_3Cl_2 26

2.9 Procedure for filtration test of immobilized Pd_3Cl_2-catalyzed in a standard Suzuki-Miyaura cross-coupling reaction 28

2.9.1 Quantification of palladium leached in solution, obtained from ICP analysis of the filtrate from a reaction catalyzed by immob-Pd_3Cl_2 3, stopped by filtration at 10 minutes (> 50 % conversion) reaction time. 30

2.10 Three-phase test procedure for standard Suzuki-Miyaura cross-coupling reaction

catalyzed by both immob-Pd_3Cl_2 and Pd_3Cl_2 cluster 30

2.10.1 Synthesis procedure for *N*-succinimidyl 4-iodo-benzoate 31

2.10.2 Synthesis procedure for immobilized aminomethyl-polystyrene bound 4-iodo-benzoate 32

2.10.3 General procedure for three-phase test experiment: SMCC reaction of immobilized aryl iodide and 4-anisyl boronic acid 33

2.10.4 Validation test for three-phase test experiment 35

2.11 Procedure for testing the detachment of the Pd_3Cl_2 cluster from the resin support in the presence of excess (exogenous) triphenylphosphine ligand 38

2.12 Procedure for immobilized $[Pd_3(\mu\text{-}Cl)(\mu\text{-}PPh_2)_2(PPh_3)_3][Cl]$-catalyzed Suzuki-Miyaura cross-coupling reaction of 2,4-dibromopyridine and 4-fluoro-phenylboronic acid 39

2.13 Synthesis replication of Pd_3Cl_2 cluster synthesis using procedure reported in Li *et al.* paper 40

2.14 Detection of $[Pd_3(\mu\text{-}Ph)(\mu\text{-}PPh_2)(PPh_3)]^+$ in ESI-MS by analysing the post-reaction mixture of $Pd_3(OAc)_6$ and 6 equivalents of PPh_3 in THF 41

2.15 Investigating Halide Ligation at the Immobilised-Pd_3Cl_2 Cluster Suzuki–Miyaura Catalysis 42

2.16 Experimental and analysis procedures for XAS data. 44

2.16.1 General procedure for preparation of reaction samples of immob-Pd_3Cl_2 for EXAFS/XANES analysis 45

2.16.2 Procedure for preparation of standard reference samples for EXAFS/XANES study of immob-Pd_3Cl_2 experiments 46

2.16.3 Procedure for preparation of reaction samples of Pd_3Cl_2 for EXAFS/XANES analysis 47

2.16.4 Procedure for preparation of standard reference samples for EXAFS/XANES study for Pd_3Cl_2 experiments 49

2.17 XAS data analysis and discussion. 50

3 NMR Spectral Data 64
4 X-ray Crystallography 75
5 Literature Comparisons 78
References……………………………………………………………………..………..79

1 General Information

1.1 General Considerations:

All reactions were carried out under inert atmosphere using either standard Schlenk technique (high vacuum, liquid nitrogen trap on a standard in-house built dual line) or an argon-filled MBraun glovebox equipped with a freezer set at –30 °C for storing sensitive reagents. Room temperature is referred to reactions with no thermostatic control and with temperature varying between 19-24 °C. Reagents were purchased from Alfa Aesar, Merck, Acros Organics, Thermo Scientific, TCI, Fluorochem, Strem and Apollo Scientific and used as received unless otherwise stated. Benzene was dried over thinly-cut slices of sodium metal under flowing nitrogen over-night, distilled using a solvent still and deoxygenated by sparging with nitrogen. Tetrahydrofuran (THF) was dried over sodium wire/9-fluorenone under flow of nitrogen for > 12 hours, distilled using a solvent still and deoxygenated by sparging with nitrogen. Dichloromethane (CH_2Cl_2) was dried over calcium hydride under flowing nitrogen for > 12 hours, distilled using a solvent still, deoxygenated by sparging with nitrogen and stored over activated 4 Å molecular sieves. Dichloromethane-d_2 (CD_2Cl_2) was dried over calcium hydride for 24 hours, deoxygenated by freeze-pump-thaw cycling and distilled into an ampoule. Aniline was dried over calcium hydride overnight, vacuum-distilled, deoxygenated by sparging with nitrogen and stored in an ampoule covered with aluminium foil. Chloroform-*d* ($CDCl_3$) was purchased from sigma Aldrich and used as received without any further purification. Solvents such as hexane, pentane and diethyl ether were obtained from a Pure Solv MD-7 solvent system and deoxygenated by sparging with nitrogen. Tetra-n-butylammonium hydroxide ($n$$Bu_4NOH$)

was purchased from Sigma Aldrich (1.5 M aqueous solution), diluted to 1.0 M using deionised water and deoxygenated by sparging with nitrogen; the solution was sonicated for 15 minutes before use to avoid crystallisation. Metallic Hg was used from an in-house recycled Hg supply (caution: Hg is fatal if inhaled and can cause damage to organs and the environment; precautions for handling Hg include using a high airflow fumehood and all residues, glassware and consumables contacted with Hg being decontaminated according to local regulations). All experiments with metallic Hg were carried out under a nitrogen atmosphere using standard Schlenk techniques (high vacuum, liquid nitrogen trap on a standard in-house built dual line). $[Pd_3(\mu\text{-}Cl)(\mu\text{-}PPh_2)_2(PPh_3)_3]Cl$ was synthesised according to an adapted literature procedure.[1],[21,2] $Pd(OAc)_2$ (indicated as >99% purity based on Pd) was purchased from Precious Metals Online (PMO) and used as received. 4-Fluoro-1-bromobenzene was purchased from Alfa Aesar and used as received. 4-Anisyl boronic acid was purchased from Apollo Scientific and used as received. Trimethoxybenzene (Merck) and 4,4'-difluoro-benzophenone were used as internal standards and the absence of their reactivity with the reaction reagents and products was individually confirmed by ^1H and ^{19}F NMR. A Thermo Scientific-purchased dry-bath thermo-shaker with mixing of 500 rpm, heating at 50 °C was used for preparation of immobilized Pd_3Cl_2 cluster.

1.2 Laboratorial analysis

Thin layer chromatography (TLC) was carried out using aluminium–backed Merck 5554 silica gel 60 F254. TLC plates were visualised by exposure to UV light (254 nm) and staining with potassium permanganate if necessary. Retention factor (R_f) values reported were measured in a developing chamber containing the solvent system described. Flash column chromatography was performed using Fluorochem silica gel P60 (35−75 μm particle size, 220-440 mesh), unless specified 'silica' refers to P60 grade silica gel.

1.3 Instrumentation

Solution NMR spectra were recorded using a 24-position SampleCase-automated Bruker Avance Neo 700 instrument (^1H 700 MHz, ^{31}C 176 MHz, ^{19}F 658 MHz) for kinetic analyses, Bruker AVIIIHD 600 wide-bore (^1H 600 MHz, ^{31}C 150 MHz, ^{31}P 242 MHz, ^{19}F 564 MHz) and Bruker AVIIHD 500 spectrometer (^1H 500 MHz, ^{31}C 125 MHz, ^{31}P 202 MHz, ^{19}F 470 MHz) for reaction outcome analyses. Solid-state NMR spectra were recorded using a Bruker AVIII400HD spectrometer (^1H 400 MHZ, ^{13}C 101 MHz, ^{31}P 162 MHz). ^1H NMR chemical shift δ are reported in ppm (parts per million), relative to the resonance shift of residual protio-

forms of deuterated solvents (C**H**Cl_3, δ 7.26 ppm and C**H**DCl_2 δ 4.32 ppm). $^{13}C\{^{1}H\}$ NMR chemical shift δ are given in ppm, relative to the resonance shift of the solvents used (**C**DCl_3, δ 77.1 ppm and **C**D_2Cl_2 δ 54.0 ppm), and reported to one decimal point. ^{19}F and $^{19}F\{^{1}H\}$ NMR chemical shift δ are reported in ppm, relative to the resonance shift of an external solvent C**F**Cl_3, at δ 0.0 ppm. $^{31}P\{^{1}H\}$ NMR chemical shifts reported in ppm, relative to the resonance shift of an external solvent H_3PO_4 at δ 0.0 ppm. Inversion-Recovery experiments were conducted for ^{1}H and ^{19}F nuclei of the compounds and internal standards involved in the model SMCC conditions (*vide infra*) in each spectrometer to measure spin-lattice relaxation time and hence the required D1 setting; this was defined as 30 s and 25 s for ^{1}H and ^{19}F experiments respectively. The magic angle spinning (MAS) probe, 4 mm probe MASDVT400W1 BL4 X/Y/F-H with pulse program CP-MAS, was used for solid-state ^{31}P NMR experiment for characterisation of immobilized Pd_3Cl_2 cluster, with spinning speed 10 KHz and D1 8 s set for the experiment. NMR data are represented as follows: chemical shift (δ), multiplicity (s = singlet, d = doublet, t = triplet, q = quartet, p = pentet, hept = heptet, m = multiplet, br = broad), coupling constant (*J*) in Hertz (Hz). All NMR spectra taken at 298 K (25 °C) unless otherwise stated.

ESI mass spectra were collected using a Bruker compact TOF (with no chromatography happening), fitted with an electrospray source. Liquid injection field desorption ionisation (LIFDI-MS) was measured using an JEOL AccuTOF GCx-plus, TOF fitted with a LIFDI probe manufactured by Linden CMS. Samples for ESI and LIFDI mass spectra were reported with <5 ppm error and mass to charge ratios (*m/z*) reported in Daltons. The measurements were conducted in positive ion mode (+ve) with scan ranges of m/z 0-500 and m/z 500-2000.

Fourier-transform attenuated total reflection (FT-ATR) spectra were recorded on a Bruker ALPHA-Platinum FTIR spectrometer using a platinum-diamond ATR sampling module, reported in terms of frequency of absorption (cm^{-1}).

Melting points were measured using a Stuart digital SMP3 apparatus.

Elemental analysis, for quantitative determining of carbon, hydrogen and nitrogen elements was carried out on an Exeter Analytical CE-440 analyser, used with a Sartorius SE2 analytical balance. Samples are run as triplicates to obtain a mean value for the result and to confirm the reproducibility of the results. Having the mass percentage of C, H, N in a sample and knowing the ratio of the other elements, the mass percentage of Pd in the sample was calculated for quantitative use of the immobilized Pd_3Cl_2 cluster in the SMCC reaction, see section 2.5.

For XAS details, see Section 2.17.

2 Experimental Details

2.1 Synthesis Procedure for [Pd3(μ-Cl)(μ-PPh2)2(PPh3)3][Cl] "Pd3Cl2" Cluster[1]

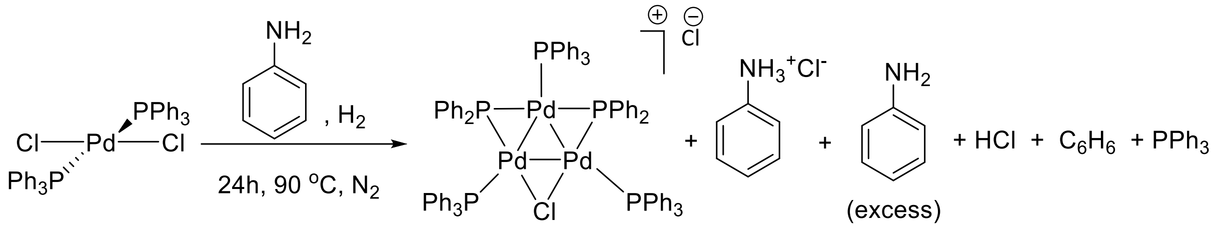

To an oven-dried Schlenk flask equipped with a stirrer bar, *trans*-$PdCl_2(PPh_3)_2$ (2.5 g, 3.57 mmol, 1 eq.) was added and the flask was evacuated and backfilled with dry N_2 three times. Aniline (50 mL) that had been separately dried over CaH_2 overnight, distilled under vacuum and degassed by N_2 bubbling, was carefully added to the reaction flask (appropriate gloves used for handling aniline). The reaction mixture was heated up to 90 °C under stirring, forming a homogenous orange solution. At this point, to fill the flask with H_2, the headspace of the flask was carefully evacuated twice and H_2 was added to the sealed flask using a balloon and needle to backfill the headspace. The change of the color to dark red was observed after one hour. The reaction was continued for 24 hours; completion of the reaction was confirmed by ^{31}P NMR analysis. After completion, the hydrogen balloon was removed and the flask was allowed to cool down to room temperature. The reaction mixture was transferred to a vacuum distillation apparatus to remove aniline; this needs to be swift to avoid reversal of the reaction or product degradation. The dried red solid was washed with Et_2O (3 × 20 mL) and hexane (2 × 20 mL) and extracted thoroughly with dried and degassed benzene (3 × 20 mL); extra care is required when handling benzene. The benzene extract was concentrated in vacuo and the dark red solid was crystallised by layering from CH_2Cl_2 and hexane (1/3, v/v). The obtained crystallised product mass was 912 mg (yield of recrystallisation 49%).

The ABB'XX' spin system approximated as AB_2X_2, where A = phosphine distal to Cl, B = phosphine proximal to Cl, and X = μ-phosphide.

^{1}H NMR (500 MHz, CD_2Cl_2) δ 7.39-7.34 (tt, *J* = 7.4, 0.95 Hz, 6H, *para*-B), 7.33-7.26 (t, J = 7.4 Hz, 4H, *para*-X), 7.19 – 7.11 (tt, *J* = 7.4 Hz, 12H, *ortho*-B), 7.11-6.95 (m, 23H, *12H-meta*-B, *8H-ortho*-X, and *3H-para*-A), 6.78-6.73 (m, 6H, *ortho*-A), 6.73-6.66 (q, *J* = 12.3, 5.8 Hz, 8H, *meta*-X) 6.58-6.52 (m, 6H, *meta*-A). ^{31}P NMR (203 MHz, CD_2Cl_2): δ 221.7-221.2 (td, *J* = 16.6, 10.0 Hz, $2PPh_2$, 2X), 19.1-18.2 (dt, *J* = 89.3, 16.6 Hz, $2PPh_3$, B), 12.4-11.2 (tt, *J* = 89.3, 10.0 Hz, $1PPh_3$, A). ^{13}C NMR (126 MHz, CD_2Cl_2) δ 135.3 (t, *J* = 12.8 Hz, *ipso*-C), 134.4 (t, *J* = 6.3 Hz, *meta*-B), 133.4 (s, *meta*-A), 133.3 (t, *J* = 8.8 Hz, *meta*-X), 132.6-132.2 (m, *J* = 21.4 Hz, *ipso*-C), 132.0 (d, J= 40.6 Hz, *ipso*-C), 130.9 (s, *para*-B, overlapped with *para*-X), 130.9

(s, *para*-X, overlapped with *para*-B), 130.2 (d, J = 2.2 Hz, *para*-A), 128.68 (t, J = 5.2 Hz, *ortho*-X), 128.66 (s, *ortho*-B), 128.2 (d, J = 10.4 Hz, *ortho*-A).

ESI-MS Data (+ve mode) [M^+]: Found 1511.0597 Calc. (for $C_{78}H_{65}ClP_5Pd_3$) 1511.0576. IR {ν, ATR, solid state, cm^{-1}}: 3048 (w), 1711 (m), 1572 (w), 1478 (m), 1433 (s), 1093 (s), 1027 (w), 995 (w), 738 (s), 687 (s), 522 (s), 487 (s), 452 (w). Melting point (°C): 146.4-150.2 (at 150.2 °C it degraded to a black material).

2.1.1 Counterion Exchange of [Pd_3(μ-Cl)(μ-PPh_2)$_2$(PPh_3)$_3$][Cl] and Formation of [Pd_3(μ-Cl)(μ-PPh_2)$_2$(PPh_3)$_3$][SbF_6] (reference compound)

To an oven-dried Schlenk flask equipped with a stirrer bar, [Pd_3(μ-Cl)(μ-PPh_2)$_2$(PPh_3)$_3$][Cl] (402 g, 0.26 mmol, 1 eq.) and $NaSbF_6$ (134.5 mg, 0.52 mmol, 2 eq.) were added and the flask was evacuated and backfilled with dry N_2 three times. Degassed EtOH (10 mL, 26 mM), which was dried over 4 Å molecular sieves, was added to the reaction flask via syringe, forming a red-coloured solution. The reaction was stirred at room temperature for 1 h and the precipitated NaCl was removed from reaction mixture via canula filtration. The red homogenous reaction mixture was concentrated to dryness under vacuum and washed with Et_2O (3 × 10 mL) and hexane (3 × 10 mL). The resulting red solid was dissolved in CH_2Cl_2 (3 mL) and set for crystallisation by layering with hexane (15 mL, 1:5 v/v) for further purification. The product $Pd_3Cl.SbF_6$ was obtained in 350 mg (77% yield) as a bright red solid.

^1H and ^{31}P NMR spectrum of $Pd_3Cl.SbF_6$ were similar to those of Pd_3Cl_2, with no considerable change in chemical shift and splitting pattern of signals, confirming no counterion interaction with the cationic Pd_3 cluster motif.

^{19}F NMR (470 MHz, 64 scans, CD_2Cl_2): δ -134.7 – -113.8 (sep, 6F, SbF_6). Elemental analysis for $C_{78}H_{65}ClF_6SbP_5Pd_3$: Found C, 52.73 H, 3.79; Calc. C, 53.60 H, 3.75.

2.2 Synthesis procedure for functionalized polystyrene resin

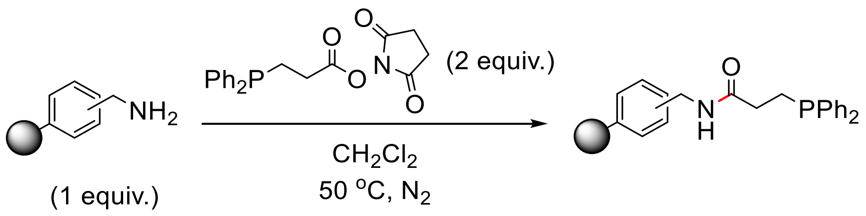

To an oven-dried septum-screw-cap vial, aminomethyl polystyrene resin (200-400 mesh) with ~ 1.5 mmol.g^{-1} amine loading (500 mg, 0.75 mmol, 1 eq.), N-succinimidyl-3-(diphosphino)propionate (533 mg, 1.5 mmol, 2 eq.) were added and purged with N_2 using a

balloon. Dried and degassed CH_2Cl_2 (3 mL) was added to the reaction vial via syringe and placed in one of the positions in a 6-well plate thermomixer. The heterogenous mixture was heated up to 50 °C and set for shaking at 500 rpm overnight. The reaction proceeded heterogeneously and after completion, the mixture was exposed to air and filtered through a sinter funnel. The filtrate solution, which contains excess ligand, was concentrated to dryness (169 mg by mass) and analysed by ^{1}H NMR spectroscopy. The solid filter was washed with dichloromethane (3 × 10 mL) and diethyl ether (5 × 5 mL) and concentrated to dryness without further purification. The functionalised resin was obtained in 97.3% yield (663.2 mg by mass) and analysed by solid-state ^{31}P NMR and ATR-IR spectroscopy. Solid-state ^{31}P NMR (162 MHz, 10kHz, D1 = 5s): δ -13.68 (br, isotropic signal), δ 47.91 (s, anisotropic side band) δ -74.17 (br, anisotropic side band). IR [ν, ATR, solid-state, cm^{-1}]: 3060, 2846, 1672, 1647, 1598, 1490, 1449, 1227, 1019, 736, 695, 534.

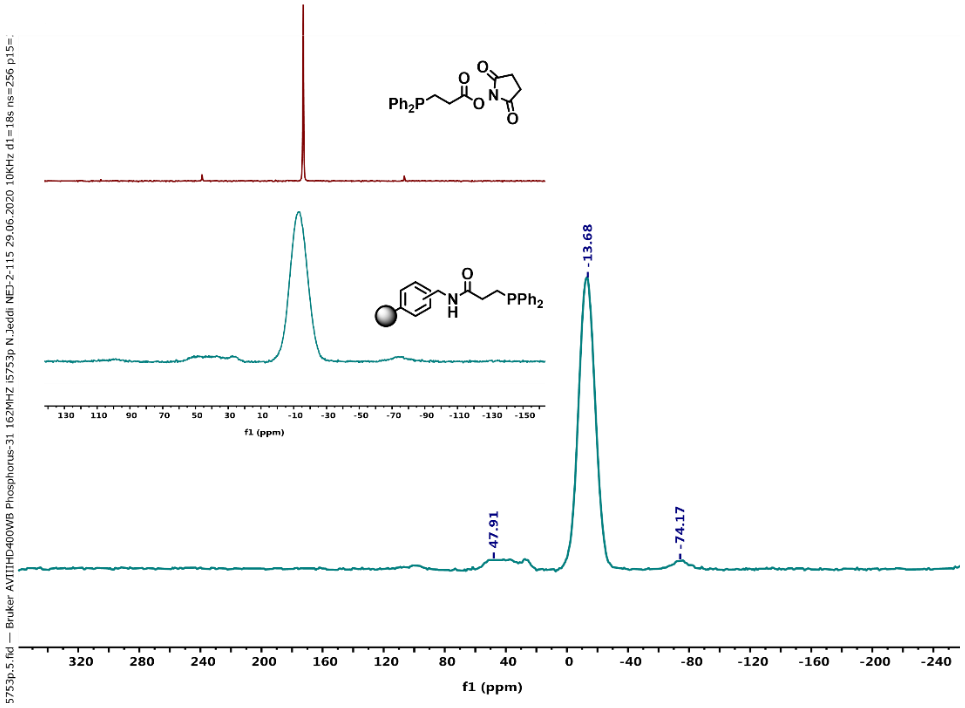

Figure S1 Solid-state ^{31}P NMR spectrum of the functionalised resin with diphenyl phosphine motif. The stack spectra compare the signal peak of N-succinimidyl-3-(diphosphino)propionate and the functionalised resin.

2.3 Synthesis procedure for immobilization of $[Pd_3(\mu\text{-}Cl)(\mu\text{-}PPh_2)_2(PPh_3)_3][Cl]$

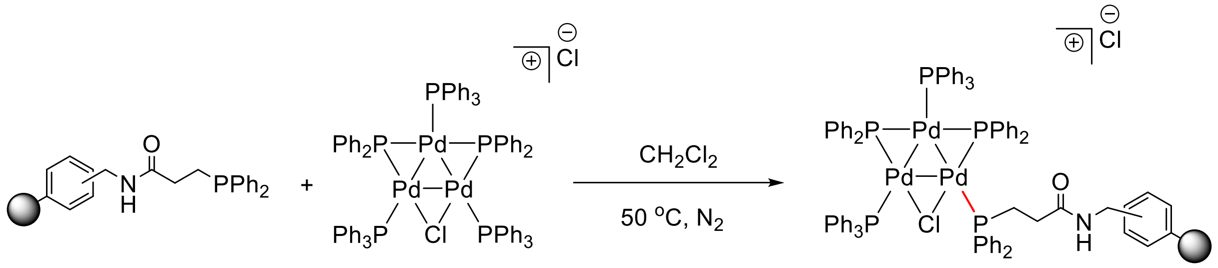

To an oven-dried septum-screw-cap vial, functionalised resin (248 mg, 0.27 mmol, 1 eq) and $Pd_3Cl.Cl$ cluster (572 mg, 0.37 mmol, 1.4 eq) were added and purged using a N_2 balloon. Dried and degassed methylene dichloride (3 mL) was added to the reaction vial via syringe and placed in one of the positions in a 6-well plate thermomixer. The heterogenous mixture was heated up to 50 °C and set for shaking at 500 rpm overnight. The reaction proceeded heterogeneously and after completion the mixture was exposed to air and filtered through a sinter funnel. The filtrate solution, which contains free PPh_3 and excess Pd_3Cl_2 (confirmed by ^1H and ^{31}P NMR spectroscopy), was concentrated to dryness (410 mg by mass) and set aside for recycling the Pd_3Cl_2 cluster by crystallisation. The solid filter was washed with dichloromethane (3 × 10 mL) and diethyl ether (5 × 5 mL) and concentrated to dryness without further purification. The red solid immobilized Pd_3Cl_2 cluster was obtained in 32.5% yield (193.1 mg by mass) and analysed by ATR-IR and solid-state ^{31}P NMR spectroscopy.

Solid-state ^{31}P NMR (162 MHz, 10kHz, D1 = 5s): δ 215.2 (br, 2P, PPh_2-bridged), δ 339.4, 275.1, 152.3, 92.5 (br, anisotropic side band for 2 PPh_2-bridged), δ 16.8 (br, 3P, distal PPh_3, proximal PPh_3 and proximal PPh_2), δ 81.4, -43.6 (br, anisotropic side band for distal PPh_3, proximal PPh_3 and proximal PPh_2). IR {ν, ATR, solid-state, cm^{-1}}: 3085, 3060, 3030, 2912, 2846, 1662, 1645, 1598, 1490, 1450, 1097, 1019, 736, 695, 515, 483, 477. Solid UV-vis (reflectance mode): λ_{max} = 590 nm.

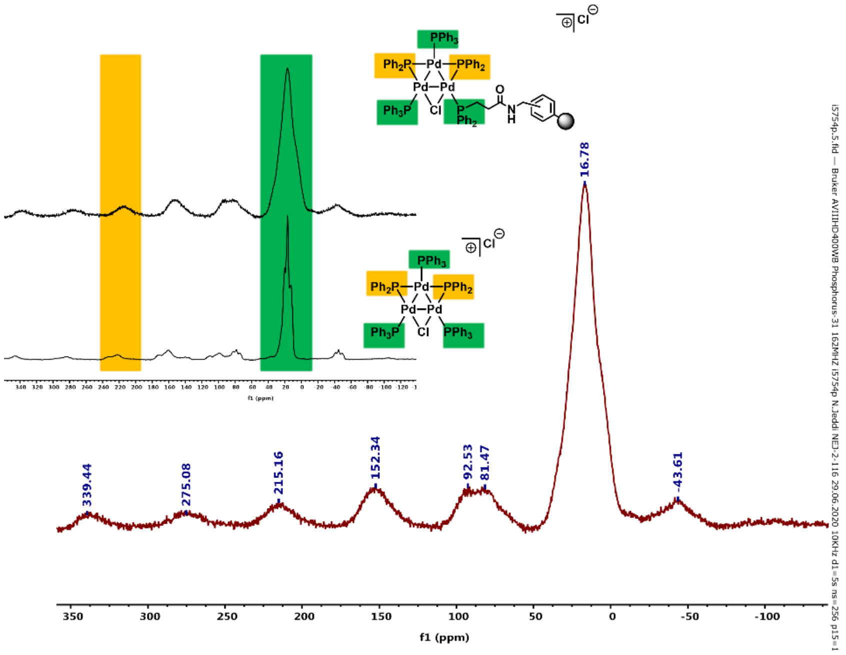

Figure S2 Solid-state ^{31}P NMR spectrum of the immobilized Pd_3Cl_2 cluster. The solid-state ^{31}P NMR spectra stack inset compares the phosphine signals of immobilized Pd_3Cl_2 (top) with Pd_3Cl_2 cluster (bottom).

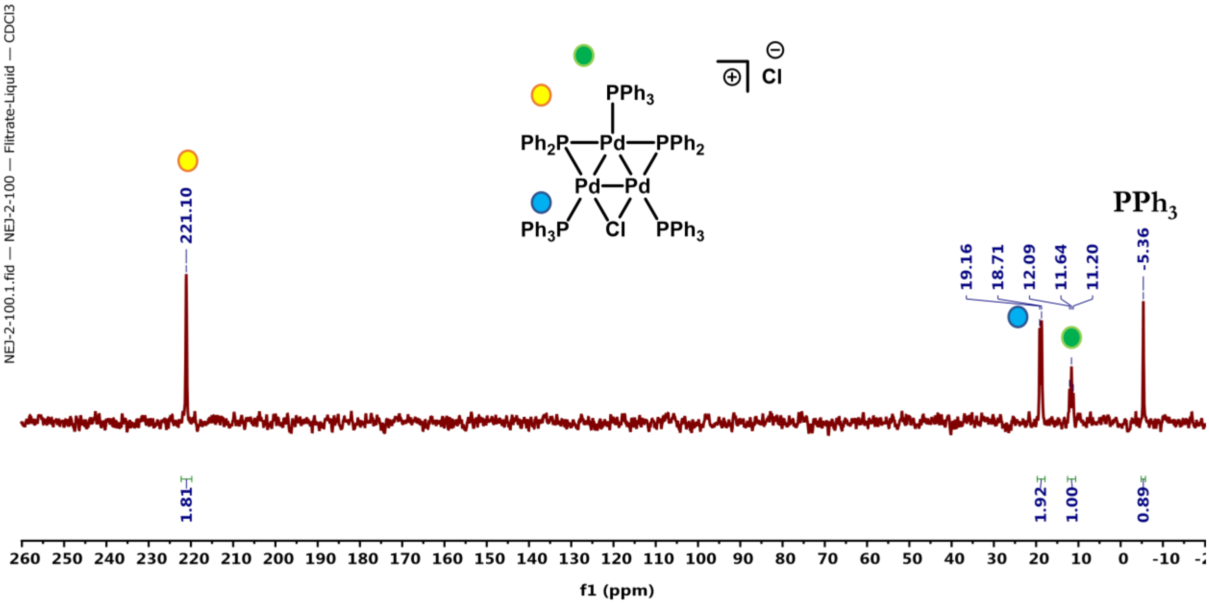

Figure S3 ^{31}P NMR ($CDCl_3$) spectrum recorded from filtrate reaction solution, showing liberated PPh_3 and excess Pd_3Cl_2.

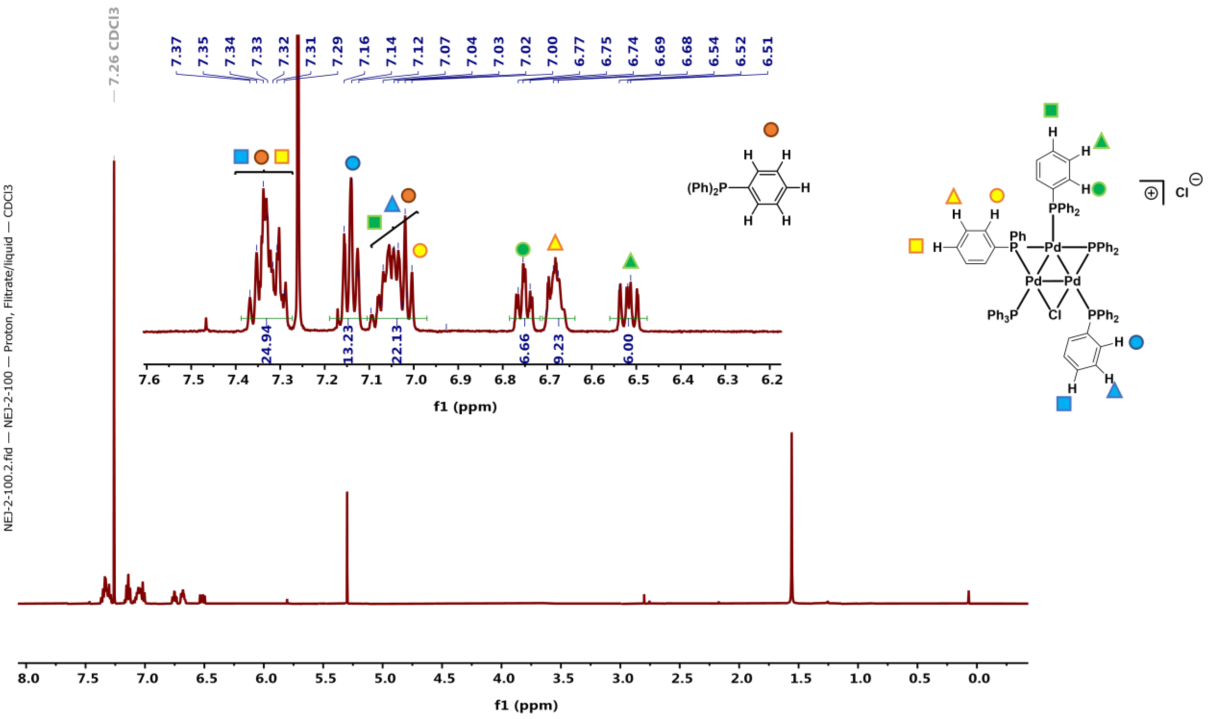

Figure S4 ^{1}H NMR ($CDCl_3$) spectrum recorded from filtrate reaction solution showing liberated PPh_3 and excess Pd_3Cl_2.

2.4 General procedure for immobilized [Pd_3(μ-Cl)(μ-PPh_2)$_2$(PPh_3)$_3$][Cl]-catalyzed Suzuki-Miyaura cross-coupling reaction of 4-bromo-1-fluorobenzene and 4-methoxy-phenylboronic acid

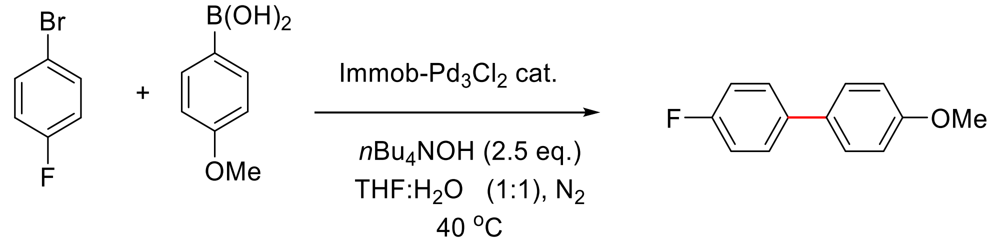

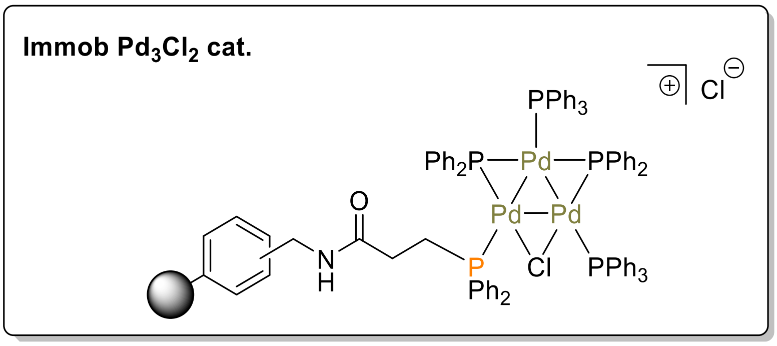

To an oven dried Schlenk flask equipped with a stirrer bar, 4-bromo-1-fluorobenzene (1.0 mmol, 110 μL, 1 eq.), 4-anisyl boronic acid (1.2 mmol, 182.35 mg, 1.2 eq.), immobilised Pd_3 cluster (10 mg, 1 mol% Pd), 1,3,5-trimethoxybenzene (84 mg, 0.5 mmol, 0.5 eq., internal standard) and 4,4'-difluorobenzophenone (109 mg, 0.5 mmol, 0.5 eq., internal standard) were added and the reaction flask was evacuated and backfilled with N_2 three times. Dry, degassed THF (2.5 mL) was added via syringe to the reaction flask. A heterogenous mixture was formed as the reaction temperature reached 40 °C with magnetic stirring. To this solution, a degassed aqueous solution of tetra-*n*-butylammonium hydroxide (2.5 mL, 1.0 M) was added via a syringe - *n*Bu_4NOH was sonicated for *ca*. 15 minutes before adding to the reaction mixture to avoid solidification (gelling) of the base during the transfer. The reaction was stirred at the set temperature for the specified time and samples were taken as indicated. After completion, the reaction mixture was allowed to cool to room temperature and quenched with a saturated aqueous solution of NH_4Cl (5 mL). The crude reaction mixture was subsequently transferred to a separating funnel for the work-up process and extracted with EtOAc (3 × 5 mL). The extract solution filtered through a celite plug (2 cm depth) on a sintered funnel before concentration *in vacuo*. The crude solid was purified by column chromatography using hexane and ethyl acetate (97.5/2.5 v/v, R_f = 0.115) and white solid obtained in 62.1% yield (isolated product).

^{1}H NMR (600 MHz, $CDCl_3$): δ 7.52 – 7.44 (m, *J* = 8.80 Hz, 4H, 2CH-*aryl*-CF, 2CH-*aryl*-CF), δ 7.12-7.07 (t, *J* = 8.80, 3.10, 2.16 Hz, 2H, 2CH-CH-$COCH_3$), δ 6.99-6.95 (d, *J* = 8.80 Hz, 2H, 2CH-$COCH_3$), δ 3.85 (s, 3H, OCH_3). ^{19}F NMR (564 MHz, $CDCl_3$): δ -116.76 (s, F-*aryl*). ^{13}C NMR (150 MHz, $CDCl_3$): δ 163.0-161.4 (d, ^{1}J = 245.6 Hz, C-F), 159.3 (s, $COCH_3$), 137.1 (d, ^{4}J = 3.5 Hz, *aryl*-C-C-*aryl*), 133.0 (s, *aryl*-C-C-*aryl*), 128.3 (d, ^{3}J = 8.4 Hz, -CH-CH-CF), 128.2 (s, CH), 115.7-115.6 (d, ^{2}J = 21.5 Hz, CH-CF), 114.4 (s, CH), 55.5 (s, OCH_3). Melting Point (°C): 90.9-92.4.

2.5 Calculation of palladium wt.% within immobilized Pd_3Cl_2 cluster using elemental analysis along with subsequent ppm and mol% calculation of palladium in Suzuki-Miyaura Cross-coupling Reaction

The elemental analysis results of immobilized [Pd_3(μ-Cl)(μ-PPh_2)$_2$(PPh_3)$_3$][Cl], which was run each time after synthesis, were highly consistent and showed a range of 11.1% to 13.9% for calculated Pd wt.%, with most of the data obtained between 12.1-12.7% for Pd as shown below.

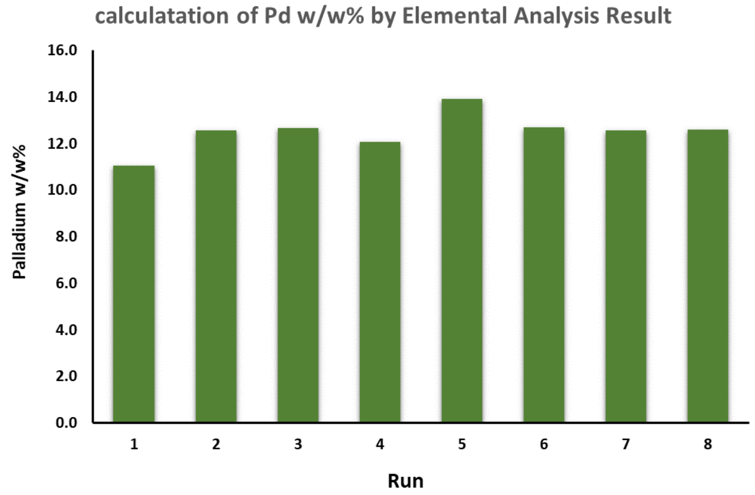

Figure S5 Consistency of Pd wt.% calculations obtained from elemental analysis results of each time the immobilized Pd_3Cl_2 was synthesised.

Based on the mass percentage of C, H, and N in the sample (from CHN analysis), an upper limit on the Pd content was calculated at 14.4 wt.% assuming full conversion to species **3** (and accounting for the relative masses of P, Pd, Cl and O atoms that would be additionally present). It is possible the Pd contents are actually slightly lower if there is either incomplete conversion or some clusters in which multiple positions undergo substitution by the resin bound phosphines (but substitution at 2 positions rather than one would, for example, change a loading of 12.6% Pd to 12.3% Pd so is not a large effect). Pleasingly as seen in Figure S5, all the Pd loadings from each synthesis, lie slightly under the theoretical maximum loading of 14.4% Pd,

also suggesting that most clusters are mono-phosphine substituted at the resin. It should be noted that based on the ^{31}P NMR results showing all the immobilised phosphine interacts with Pd, that any variance must be the result of some di- (or even tri-) substituted clusters, but the tethering phosphine is deliberately chosen to be sterically and electronically like the original PPh_3 in Pd_3Cl_2 **1**, thereby minimising differences in reactivity.

Based on the quantity of the catalyst used in SMCC reaction (here in one example 10 mg) and quantity of all reagents, additives and solvents, the mol% and ppm of the palladium was calculated as shown below.

Table S1 Calculation of mol% and ppm of palladium in immob-Pd_3Cl_2 catalyst used in SMCC reaction, based the wt.% of palladium calculated from elemental analysis results.

Mass of immobilized Pd_3 used in SMCC reaction (mg)	Pd wt% calculated in NEJ-3-279	Mass of Pd element in catalyst used in SMCC (mg)
10	12.571	1.2571

Catalyst made from NEJ-3-279							
	equivalent	Volume (mL)	Mw ($gmol^{-1}$)	Density (gmL^{-1})	Pd mass used (mg)	mmol	ppm of Pd in SMCC reaction
4-bromo-1-fluorobenzene	1		175.000	1.593		1.000	
4-MeO-PhB(OH)$_2$	1.2		151.960			1.200	
nBu_4NOH	2.5		259.470			2.500	
THF		2.5	72.110	0.889		30.821	
H_2O		2.5	18.010	0.997		138.395	
Pd			106.420		1.2571	0.0118	**68.073**

2.6 Kinetic assay analysing the effect of different quantities of Pd catalysts used in the standard Suzuki-Miyaura cross-coupling reaction.

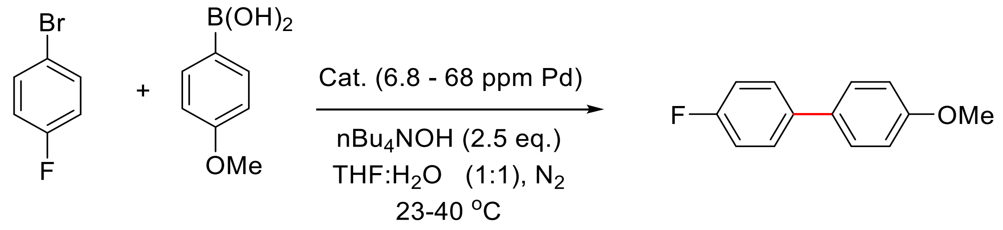

cat. immob-Pd_3Cl_2, Pd_3Cl_2, $Pd(OAc)_2$/2PPh_3, $Pd(OAc)_2$/3PPh_3, Pd_3Cl_2/resin

To an oven dried Schlenk flask equipped with a stirrer bar 4-bromo-1-fluorobenzene (1 eq.), 4-anisyl boronic acid (1.2 eq.), the catalyst, 1,3,5-trimethoxybenzene (0.5 eq., internal standard) and 4,4'-difluorobenzophenone (0.5 eq., internal standard) were added and the reaction flask was evacuated and backfilled with N_2 three times. Dry, degassed THF was added via syringe to the reaction flask. The reaction was heated to 40 °C with magnetic stirring. To

this solution, a degassed aqueous solution of tetra-*n*-butylammonium hydroxide (2.5 eq., 1.0 M) was added *via.* a syringe - nBu_4NOH was sonicated for *ca*. 15 minutes before adding to the reaction mixture to avoid crystallisation of the base during the transfer. A sample (0.1 mL) at t = 0 was taken from reaction, transferred to a vial containing saturated NH_4Cl aq. solution (5 mL) to quench and subsequently was extracted by EtOAc by small scale. The extract solution was filtered through a celite plug packed in a Pasteur pipette supported by cotton wool and then concentrated in vacuo for ^1H and ^{19}F NMR analysis. Samples were taken at short intervals and were analysed ex situ via NMR spectroscopy, using the signals that appeared in the spectra for both the product and internal standards. ^1H and ^{19}F NMR spectral analysis showed very good consistency.

2.6.1 Kinetic analysis of $Pd_3(OAc)_6/2PPh_3$ and $Pd_3(OAc)_6/3PPh_3$ as catalyst in standard SMCC reaction (68 ppm Pd)

The kinetic analysis of the model SMCC reaction catalyzed with $Pd(OAc)_2/2PPh_3$ (1:2) system, which was previously reported to involve high-order Pd species.[3] The kinetic analysis of the model reaction, catalyzed by $Pd(OAc)_2/3PPh_3$ (1:3) was also carried out to compare the results if applicable, as shown in Figure S6 and Figure S7. It should be noted the first data point is taken as soon as feasible on initial mixing but the sampling via syringe is not instant, which reasonably accounts for the non-zero conversion observed in these rapid processes.

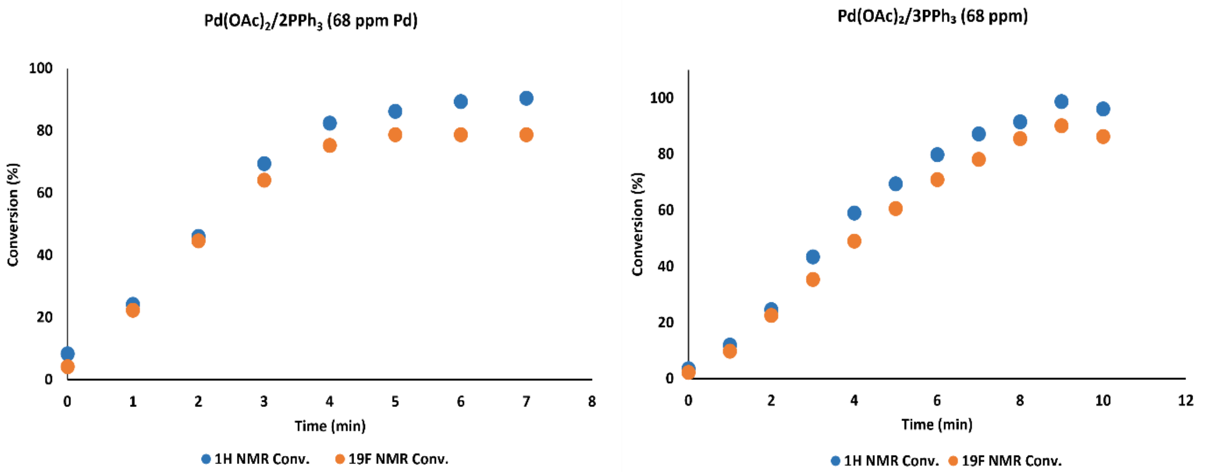

Figure S6 Kinetic profile of model SMCC reaction that is catalyzed by $Pd(OAc)_2/2PPh_3$ (left) and $Pd(OAc)_2/3PPh_3$ (right) analysed by both ^1H and ^{19}F NMR analysis.

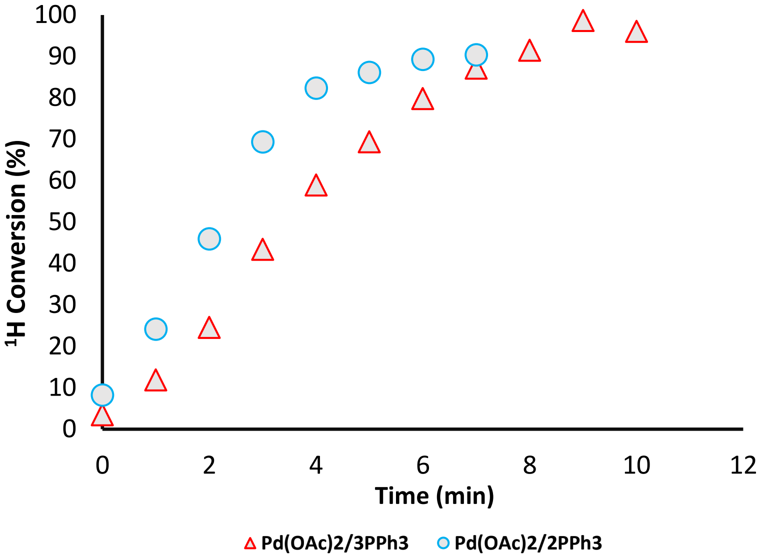

Figure S7 Overlay of kinetic data obtained from SMCC reaction catalyzed by $Pd(OAc)_2/2PPh_3$ (blue squares) and $Pd(OAc)_2/3PPh_3$ (red triangles) by *ex situ* ^1H NMR analysis.

2.6.2 Kinetic analysis of individual immobilized Pd_3Cl_2 as catalyst (24-68 ppm Pd) used in standard SMCC reaction

The individual kinetic assay of SMCC reaction that is catalyzed by immob-Pd_3Cl_2 with each quantity from 24 to 68 ppm Pd is obtained by ^1H and ^{19}F NMR analysis, shown in Figures 8-10. The reproducibility of the kinetics is demonstrated for the 68 ppm Pd catalyzed reaction (Figure 11).

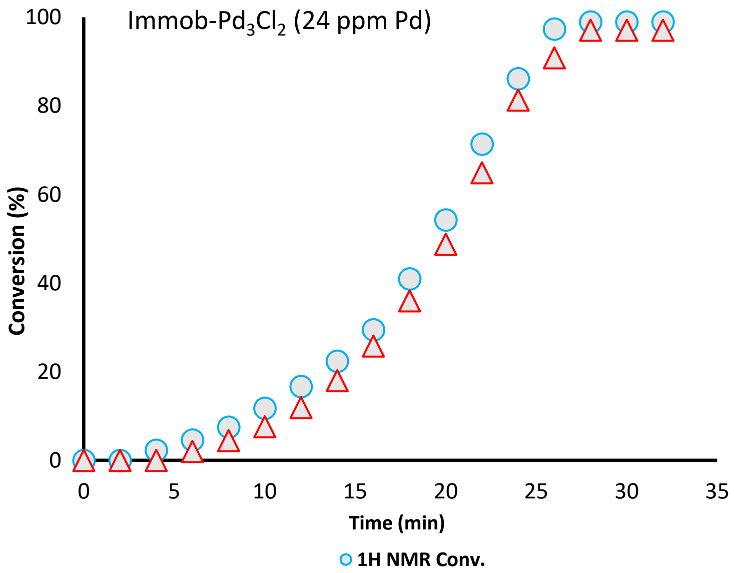

Figure S8 Kinetic profile of model SMCC reaction catalyzed by immob-Pd_3Cl_2 catalyst (24 ppm Pd), analysed by ^1H and ^{19}F NMR.

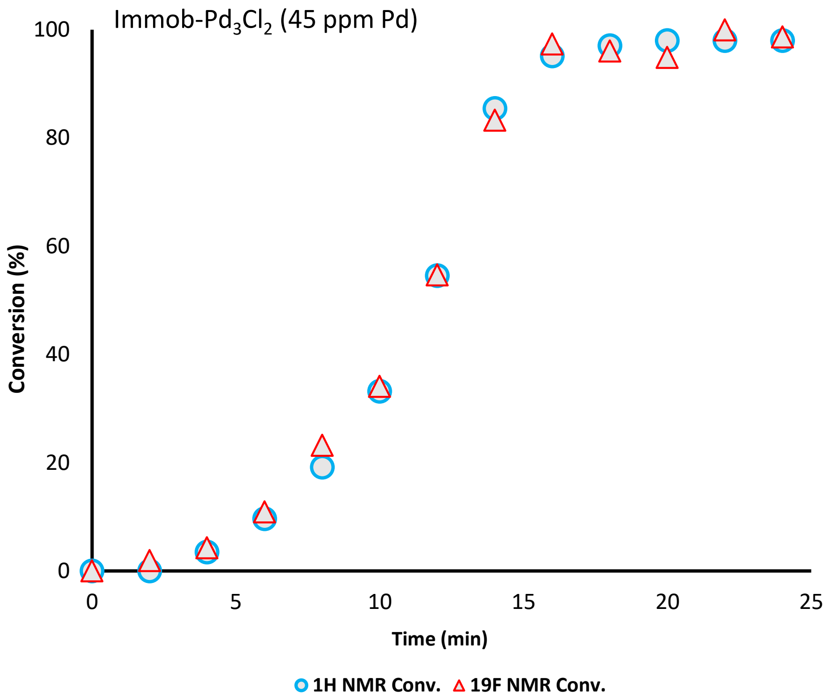

Figure S9 Kinetic profile of model SMCC reaction catalyzed by immob-Pd_3Cl_2 catalyst (45 ppm Pd), analysed by ^1H and ^{19}F NMR.

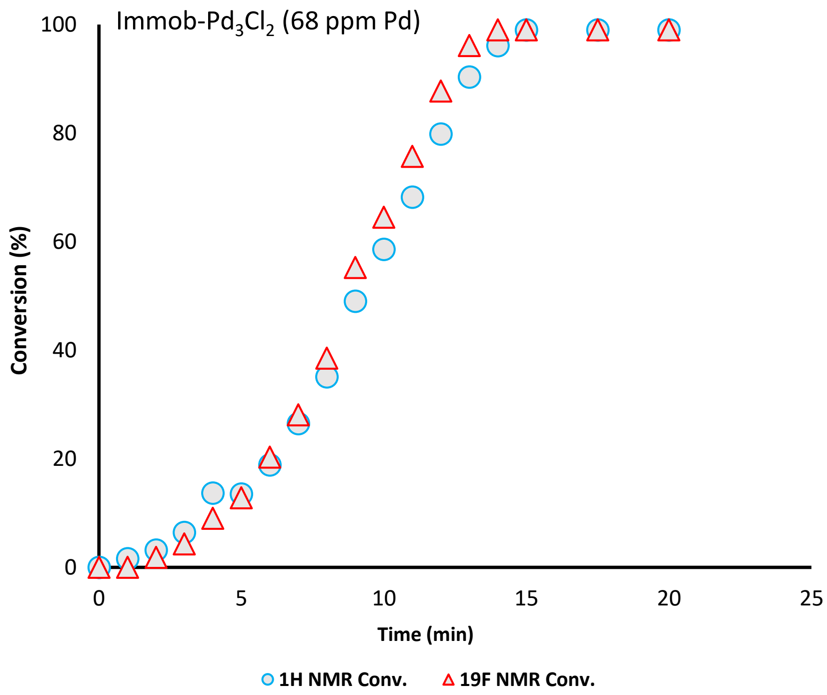

Figure S10 Kinetic profile of model SMCC reaction catalyzed by immob-Pd_3Cl_2 catalyst (68 ppm Pd), analysed by ^1H and ^{19}F NMR.

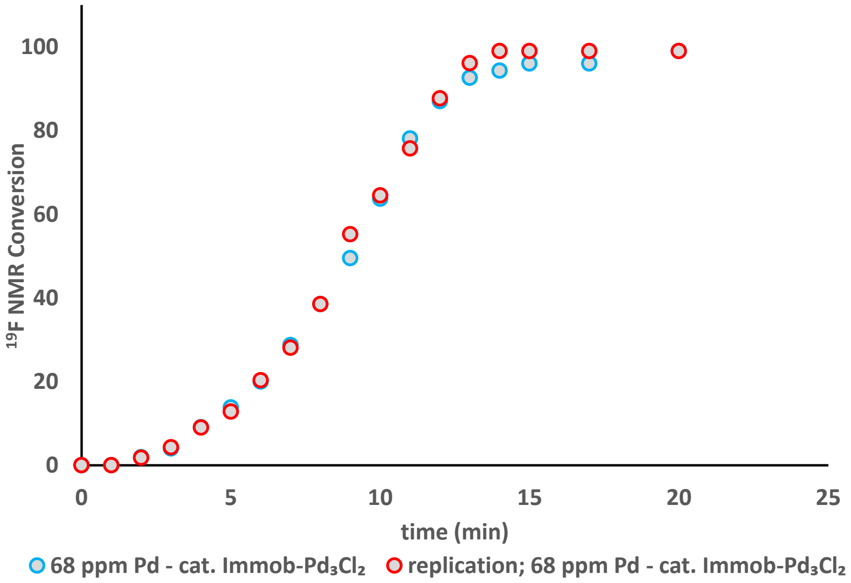

Figure S11 Reproducibility tests showing the kinetic profiles of the model SMCC reaction catalyzed by immob-Pd_3Cl_2 catalyst (68 ppm Pd), analysed by ^{1}H NMR.

2.6.3 Kinetic analysis of individual Pd_3Cl_2 as catalyst (6.8-68 ppm Pd) used in the standard SMCC reaction

The individual kinetic assay of SMCC reaction that is catalyzed by Pd_3Cl_2 with each quantity from 6.8 to 68 ppm Pd is obtained by ^{1}H and ^{19}F NMR analysis, shown as below.

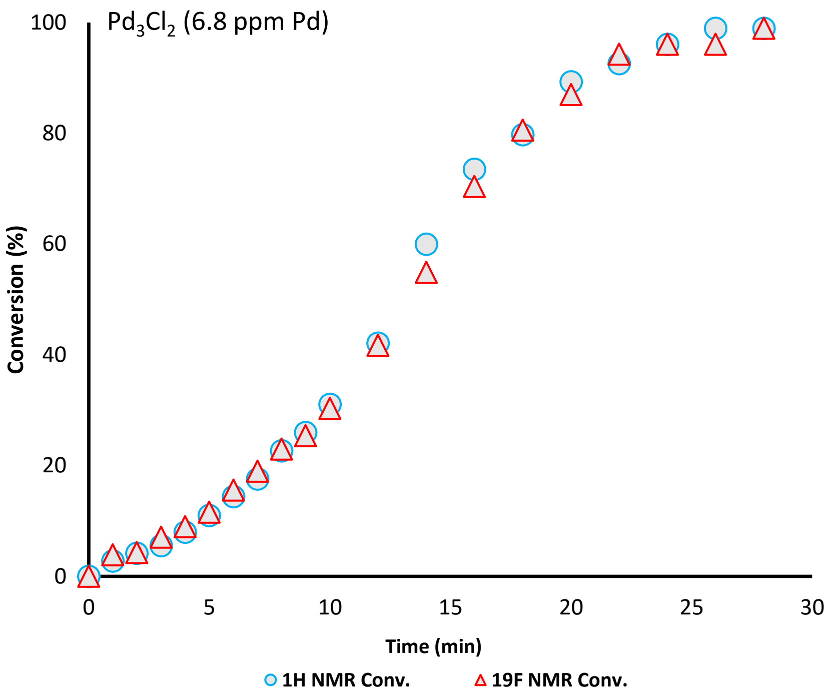

Figure S12 Kinetic profile of model SMCC reaction catalyzed by Pd_3Cl_2 catalyst (6.8 ppm Pd), analysed by ^1H and ^{19}F NMR.

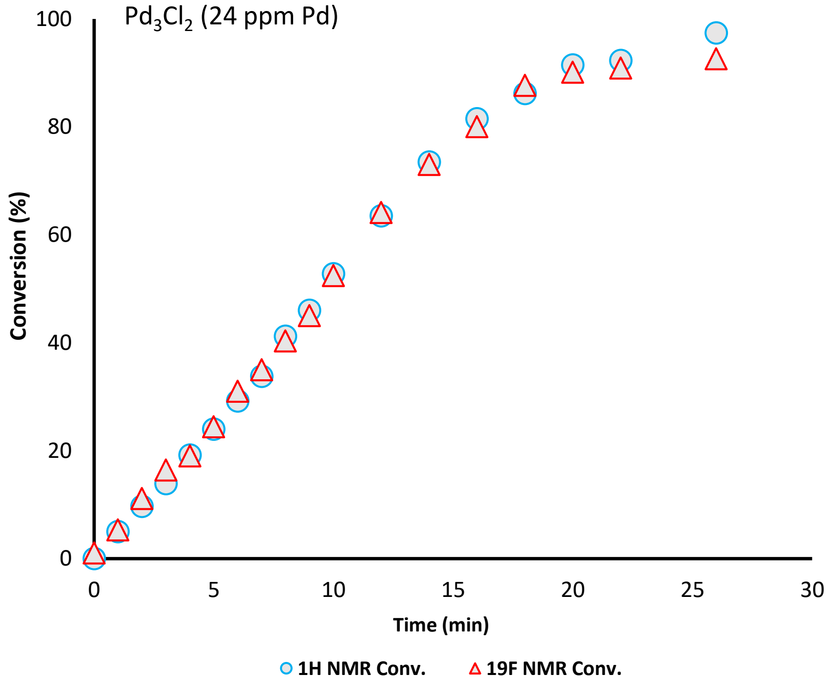

Figure S13 Kinetic profile of model SMCC reaction catalyzed by Pd_3Cl_2 catalyst (24 ppm Pd), analysed by ^1H and ^{19}F NMR.

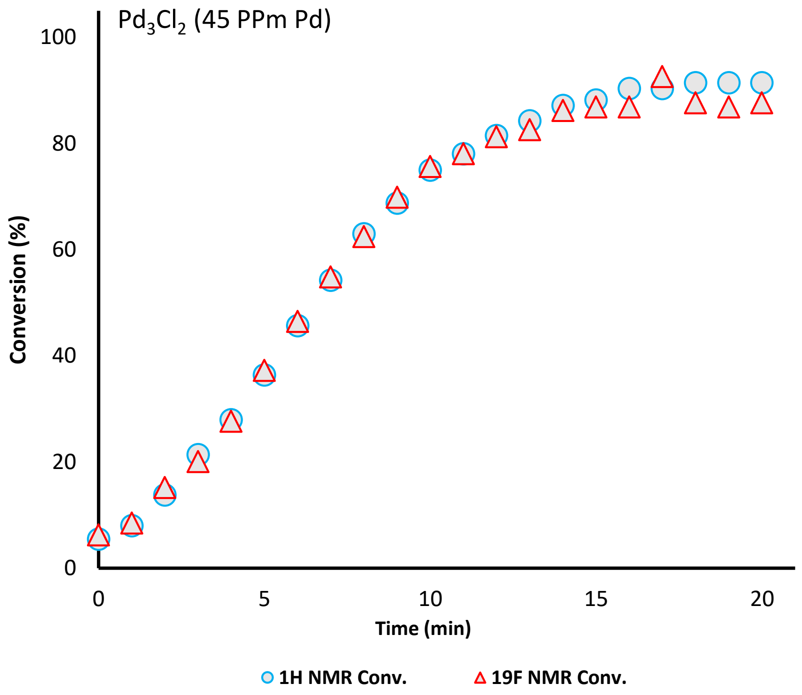

Figure S14 Kinetic profile of model SMCC reaction catalyzed by Pd_3Cl_2 catalyst (45 ppm Pd), analysed by ^{1}H and ^{19}F NMR.

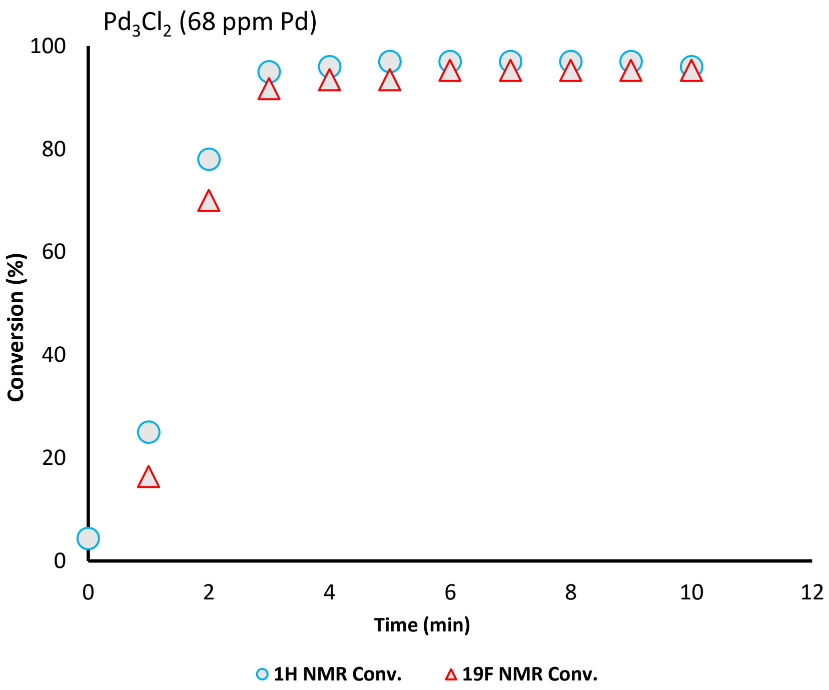

Figure S15 Kinetic profile of model SMCC reaction catalyzed by Pd_3Cl_2 catalyst (68 ppm Pd), analysed by ^{1}H and ^{19}F NMR.

2.6.4 Kinetic analysis of individual Pd_3Cl_2:resin as catalyst (68 ppm Pd) in different ratios used in the standard SMCC reaction

The individual kinetic assay of SMCC reaction that is catalyzed by Pd_3Cl_2:resin (68 ppm Pd), added separately to the reaction mixture, with mass ratios of 1:1, 1:2 and 1:3, was obtained by ^{1}H and ^{19}F NMR analysis, as shown below.

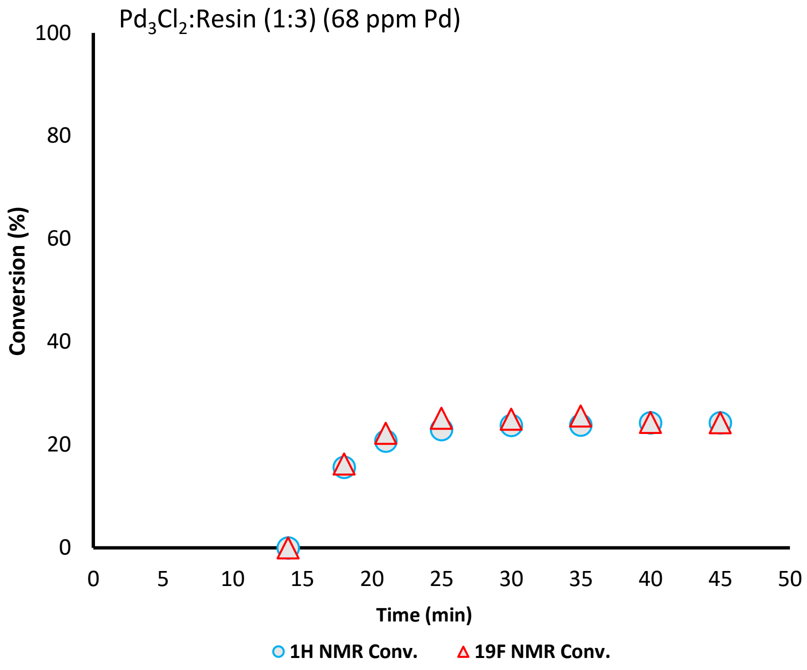

Figure S16 Kinetic profile of model SMCC reaction catalyzed by Pd_3Cl_2:resin (1:3, w/w) catalytic system (68 ppm Pd) analysed by ^{1}H and ^{19}F NMR – adding Pd_3Cl_2 to the reaction at t=15 min.

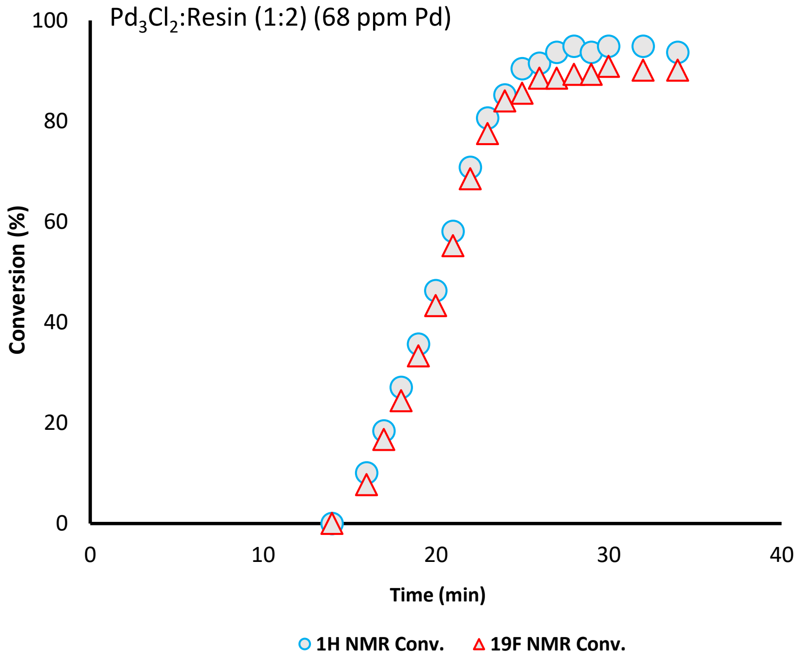

Figure S17 Kinetic profile of model SMCC reaction catalyzed by Pd_3Cl_2:resin (1:2, w/w) catalytic system (68 ppm Pd) analysed by ^{1}H and ^{19}F NMR – adding Pd_3Cl_2 to the reaction at t=15 min.

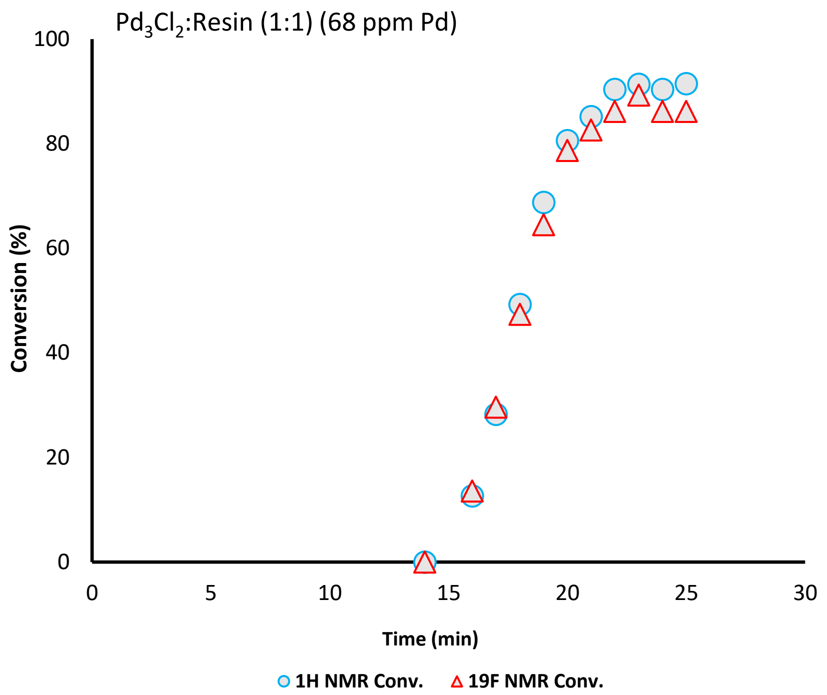

Figure S18 Kinetic profile of model SMCC reaction catalyzed by Pd_3Cl_2:resin (1:1, w/w) catalytic system (68 ppm Pd) analysed by ^{1}H and ^{19}F NMR – adding Pd_3Cl_2 to the reaction at t=15 min.

2.6.5 Kinetic analysis of Pd_3Cl_2 as catalyst (24 ppm Pd) with *n*Bu_4NBr (1 equiv.)

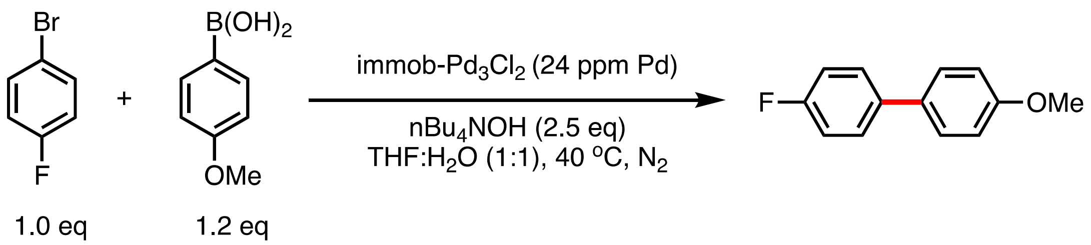

An oven dried Schlenk flask equipped with a magnetic stirrer bar was charged with 4-bromo-1-fluorobenzene (350 mg, 220 µL, 2.0 mmol, 1 eq.), 4-anisyl boronic acid (365 mg, 2.4 mmol, 1.2 eq.), immob-Pd_3Cl_2 catalyst (7.0 mg, 24 ppm), tetra-*n*-butylammonium bromide (665 mg, 2.0 mmol, 1.0 eq.), 1,3,5-trimethoxybenzene (168 mg, 1.0 mmol, 0.5 eq., internal standard) and 4,4'-difluorobenzophenone (218 mg, 1.0 mmol, 0.5 eq., internal standard) were added and the reaction flask was evacuated and backfilled with N_2 three times. Dry, degassed THF (5 mL) was added via syringe to the reaction flask and the subsequent solution was magnetically stirred at 40 °C for 30 minutes. To this solution, a degassed aqueous solution of tetra-*n*-butylammonium hydroxide (5.0 mL, 1.0 M, 2.5 eq.). base was added via syringe. From the resulting stirred solution, samples (0.1 mL) were taken via syringe from the reaction 2.5, 5.0, 7.5, 10, 15, 20, 25, 30, 40, 50, 60, 80, 100 minutes after addition of base and each transferred to vials containing NH_4Cl (5 mL; sat. aq.)

solution to quench and subsequently extracted with EtOAc (3 × 3 mL). The combined EtOAc extract solution was filtered through a celite plug (1 cm depth) packed in a Pasteur pipette, supported on cotton wool before being concentrated in vacuo and redissolved in $CDCl_3$ for NMR spectroscopic analysis.

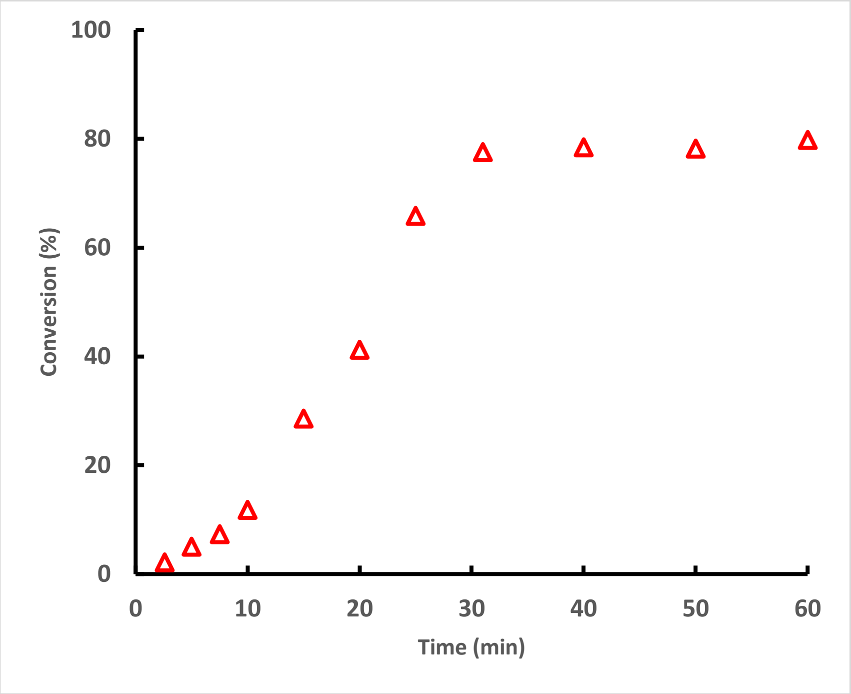

Figure S19 Kinetic profile of model SMCC reaction catalyzed by immobilised-Pd_2Cl_3 (24 ppm Pd), with additive *n*Bu_4NBr,analysed by ^{1}H and ^{19}F NMR.

2.7 Procedure for recharging substrates and reagents in the standard SMCC reaction

To an oven dried Schlenk flask equipped with a stirrer bar, 4-bromo-1-fluorobenzene (1.0 mmol, 110 µL, 1 eq.), 4-anisyl boronic acid (1.2 mmol, 182.3 mg, 1.2 eq.), solid-supported Pd_3 cluster (10 mg, 1 mol% Pd), 1,3,5-trimethoxybenzene (84 mg, 0.5 mmol, 0.5 eq., internal standard) and 4,4'-difluorobenzophenone (109 mg, 0.5 mmol, 0.5 eq., internal standard) were added and the reaction flask was evacuated and backfilled with N_2 three times. Dry, degassed THF (2.5 mL) was added via syringe to the reaction flask. A heterogeneous solution was formed as the reaction temperature was reached to 40 °C under stirring. To this solution, degassed aqueous solution of tetra-*n*-butylammonium hydroxide (2.5 mL, 1.0 M) was added via a syringe - *n*Bu_4NOH was sonicated for ca. 15 minutes before adding to the reaction mixture to avoid crystallisation of the base during the transfer. The reaction was stirred at the set temperature for the specified time and samples were taken as indicated. At t=14 min a sample was taken from the reaction mixture and at t=15 min, a new batch of 4-bromo-1-fluorobenzene

(0.5 mmol, 55 µL, 0.5 eq.), 4-anisyl boronic acid (0.6 mmol, 91.2 mg, 0.6 eq.), 1,3,5-trimethoxybenzene (0.25 mmol, 0.25 eq.), 4,4′-difluorobenzophenone (0.25 mmol, 0.25 eq.), THF (1.25 mL) and *n*Bu_4NOH (1.25 mL) were added to the reaction mixture while stirring at 40 °C. A sample was taken from the reaction mixture at t=29 min and a new batch of substrates, reagents, and solvents were again added in the same quantity at t=30 min. This was repeated for 6 times and the reaction samples were frequently taken before the fresh batches of substrates were added (Table S2).

Table S2 The timetable shown for taking samples from reaction mixture and adding fresh batch of substrates, reagents, and solvents**.**

	Time (min)					
Fresh substrates added	15	30	45	60	75	
Sample taken	14	29	44	59	74	90

2.8 Hg poisoning test kinetic assay for immobilized Pd_3Cl_2-catalyzed standard Suzuki-Miyaura cross-coupling reaction

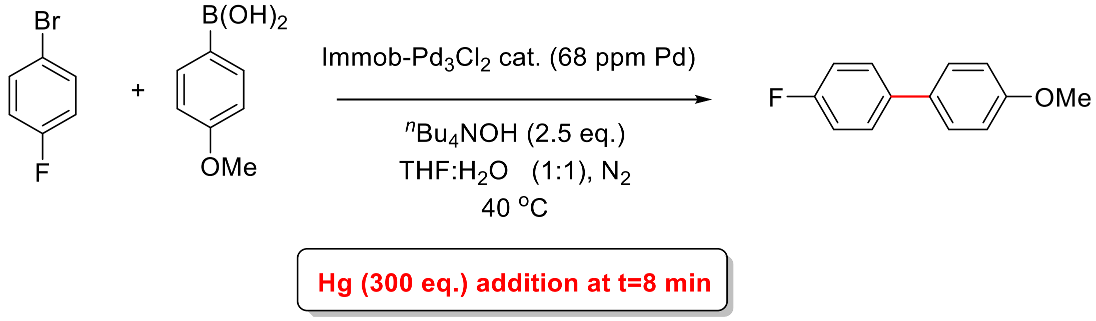

To an oven dried Schlenk flask equipped with a stirrer bar 4-bromo-1-fluorobenzene (1.0 mmol, 110 µL, 1 eq.), *4-an*isyl boronic acid (1.2 mmol, 182.35 mg, 1.2 eq.), immob-Pd_3Cl_2 cluster (10 mg, 1 mol% Pd), 1,3,5-trimethoxybenzene (84 mg, 0.5 mmol, 0.5 eq., internal standard) and 4,4'-difluorobenzophenone (109 mg, 0.5 mmol, 0.5 eq., internal standard) were added and the reaction flask was evacuated and backfilled with N_2 three times. Dry, degassed THF (2.5 mL) was added via syringe to the reaction flask. A heterogenous mixture was formed as the reaction temperature reached 40 °C under stirring. To this solution, a degassed aqueous solution of tetra-*n*-butylammonium hydroxide (2.5 mL, 1.0 M) was added via a syringe - *n*Bu_4NOH was sonicated for ca. 15 minutes before adding to the reaction mixture to avoid crystallisation of the base during the transfer. The reaction was stirred vigorously (using a standard magnetic stirrer hot plate, see Figure S20) at the set temperature and samples were taken at intervals specified as follows. A sample (0.1 mL) at t=0 was taken from reaction and transferred to a vial containing saturated NH_4Cl aq. solution (5 mL) to quench and subsequently

was extracted by EtOAc (3 x 5 mL) on small scale. The extract solution was filtered through a celite packed in a pipette supported with cotton wool and then concentrated *in vacuo* for ^{1}H and ^{19}F NMR analysis. Samples were taken at *minute* intervals until t = 7 min of the reaction; at this point, metallic Hg (50 μL, 300 eq. in respect to Pd) was added via disposable syringe at t=8 min and vigorous stirring continued. Samples subsequently were taken a *minute* intervals as follows: *i*. stirrer power was tuned off, *ii*. metallic Hg was allowed to settle down in the bottom of reaction flask (for few seconds) and *iii*. samples were taken carefully from reaction mixture without contacting Hg. Sample taking continued until reaction completion, after which the mixture was subsequently quenched, extracted, and analysed *ex situ* via NMR spectroscopy using the signals that appeared in the spectra for both the product and internal standards. It should be noted the conditions of rapid stirring and 300 eq. (a large excess) of Hg were selected to ensure intimate contact between Hg and the entire reaction system.[4] We deliberately spiked a reaction occurring during productive catalysis, ensuring we were sensitive to inhibition effects and not other factors. The test would be not informative if the reaction had reached close to full conversion (to product).

Caution: glassware and consumables used for Hg drop test, should be disposed separately, according to local and national Health and Safety regulations.

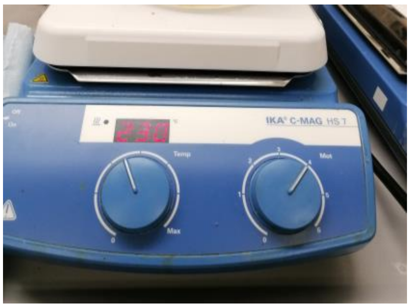

Figure S20 Standard magnetic stirrer hot plate used for Hg drop test, with vigorous stirring. The temperature of the probe (picture not shown) was set to the desired reaction temperature (*i.e.* 'bath temperature'), as is typical practice. Through control experiments on similar equipment, we estimate the temperature difference inside the reaction flask to be (±2 °C). This information is given in the revised SI document.

2.8.1 Procedure for reaction of Hg(0) with Pd_3Cl_2

An experiment designed to assess the reactivity of **Pd_3Cl_2** with excess metallic Hg (300 eq.) in THF over time.

Procedure: To an oven dried Schlenk flask equipped with a stirrer bar Pd_3Cl_2 cluster (10 mg, 0.006 mmol, 1 eq.) was added and the reaction flask was evacuated and backfilled with N_2 three times. Dry, degassed THF (3 mL, 2 mM) was added via syringe to the reaction flask. A homogenous red solution was formed and stirred under N_2 at room temperature. To this

solution, metallic Hg (15 μL, 300 eq. with respect to Pd) was added via disposable syringe and vigorous stirring continued. The reaction colour started changing after 30 min and turned to a pale-yellow solution within 2 h. Samples were taken at t=2 h and t=24 h after addition of Hg(0) and analysed by ^{31}P NMR spectroscopy.

The ^{31}P NMR spectrum of **Pd_3Cl_2** as shown in Figure S21, contains three characteristic resonances assigned to three phosphorus environments in the cluster. After addition of excess Hg(0) to a solution of **Pd_3Cl_2** in THF, the reaction colour changed from a characteristic dark red to pale yellow within 2 h. ^{31}P NMR analysis of sample taken after 2 h disclosed the complete disappearance of signals attributed to **Pd_3Cl_2** and formation of few singlets in high field regions at δ_P 20.5, 24.1, 24.7, 26.2, 32.6, and 33.7 ppm that their chemical structure remained unknown. A singlet at δ_P 24.1 ppm is indicated as triphenylphosphine oxide. The reaction after 24 h turned completely colourless and a ^{31}P NMR spectrum of the sample showed disappearance of some of the signals seen after 2 h, including a doublet centred at δ_P 33.1 ppm, which again indicated their chemical structure remained unidentified. No Hg containing compound could be detected by ESI-MS analysis.

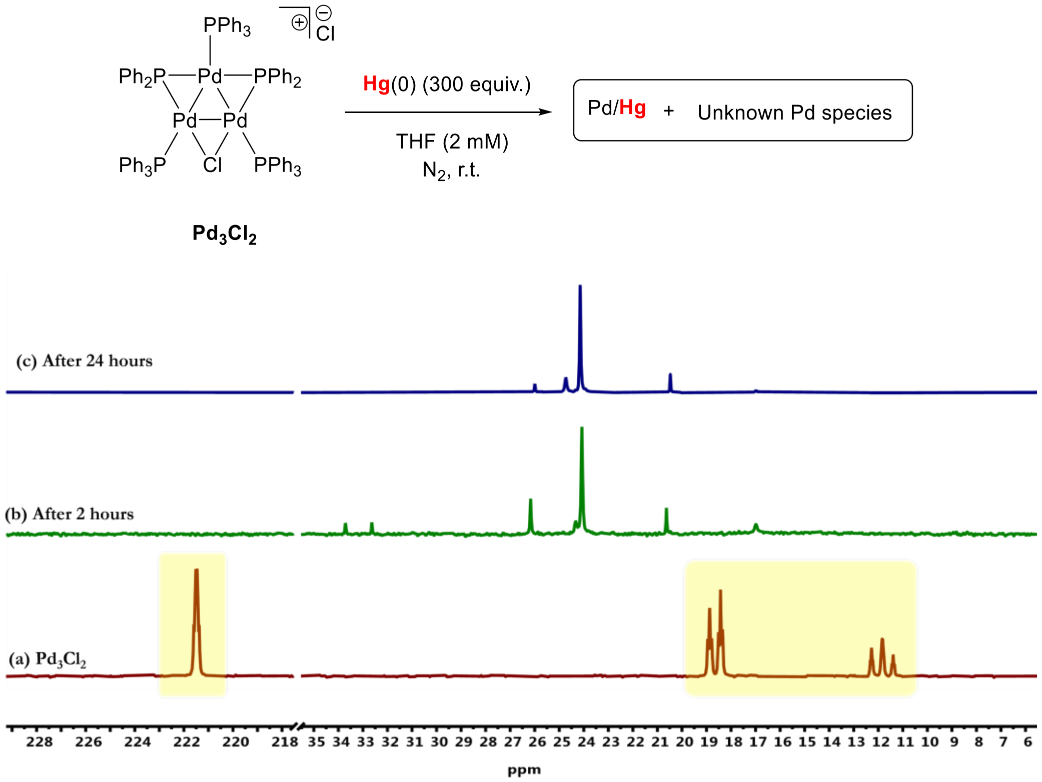

Figure S21 Reaction of excess Hg(0) with Pd_3Cl_2 in THF, showing full decomposition of the triangular Pd_3 motif and formation of few unknown species over time, confirmed by ^{31}P NMR (242 MHz, 256 scans, THF-lockoff) spectra of reaction samples taken after 2 and 24 hours of reaction.

Another Hg drop test was attempted with the model SMCC reaction promoted by **Pd_3Cl_2** as

catalyst (the lowest concentration used of 6.8 ppm was selected to slow down the catalysis), however, the experiment showed inconsistent results and was counted unreliable due to reproducibility issues. Nevertheless, the potential reaction of metallic Hg^0 with **Pd_3Cl_2** or **48** in catalytic cross-coupling reaction, in the presence of all reagents and additives, cannot not be ruled out on the basis of clear reaction of Hg^0 and **Pd_3Cl_2** cluster. In addition, the practical parameters in reaction set-up could also play a role in the outcomes of the experiment as their reproducibility have been also questioned in Ananikov's study.[5]

2.9 Procedure for filtration test of immobilized Pd_3Cl_2-catalyzed in a standard Suzuki-Miyaura cross-coupling reaction

To an oven dried Schlenk flask equipped with a stirrer bar 4-bromo-1-fluorobenzene (1.0 mmol, 110 μL, 1 eq.), 4-anisyl boronic acid (1.2 mmol, 182.4 mg, 1.2 eq.), immob-Pd_3Cl_2 cluster (10 mg, 1 mol% Pd), 1,3,5-trimethoxybenzene and 4,4'-difluorobenzophenone (0.5 eq.) as internal standards were added and the reaction flask was evacuated and backfilled with N_2 three times. Dry, degassed THF (2.5 mL) was added via syringe to the reaction flask. A heterogenous mixture was formed as the reaction temperature reached 40 °C under stirring. To this solution, degassed aqueous solution of tetra-*n*-butylammonium hydroxide (2.5 mL, 1.0 M) was added via a syringe - $n Bu_4NOH$ was sonicated for ca. 15 minutes before adding to the reaction mixture to avoid crystallisation of the base during the transfer. The reaction was stirred at 40 °C under the standard reaction condition. At t = 4 minutes, the catalyst particles were removed from the reaction mixture by filtration through a syringe filter (Nylon, 13 mm, 0.22 μm). For this, the contents of the reaction flask were transferred to a new flask (oven-dried and placed under N_2, equipped with a magnetic stirring bar); the premise of the experiment is that the catalyst particles cannot pass through the syringe filter (remaining in the original reaction flask). The filtration process did result in separating most of the catalyst particles. However, smaller particles passed through the filter (which were visible to the naked eye in the filtrate mixture). Further samples were taken at 8, 15 and 30 minutes which were assessed by ^1H and ^{19}F NMR spectroscopic analysis. The reaction continued in the new reaction flask under the standard reaction conditions used, albeit at a slower rate. While this suggests the presence of soluble leached species from immob-Pd_3Cl_2 catalyst into the post-filtered solution, the visual presence of smaller Pd particles in the second reaction flask, prevented any conclusion to be drawn based on the result of the experiment.

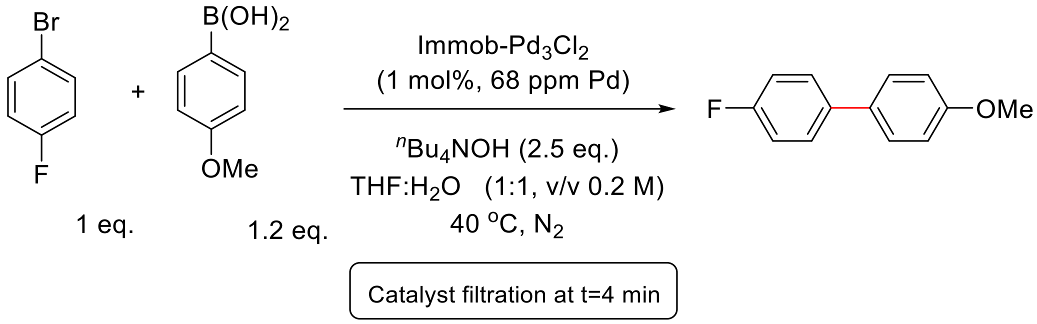

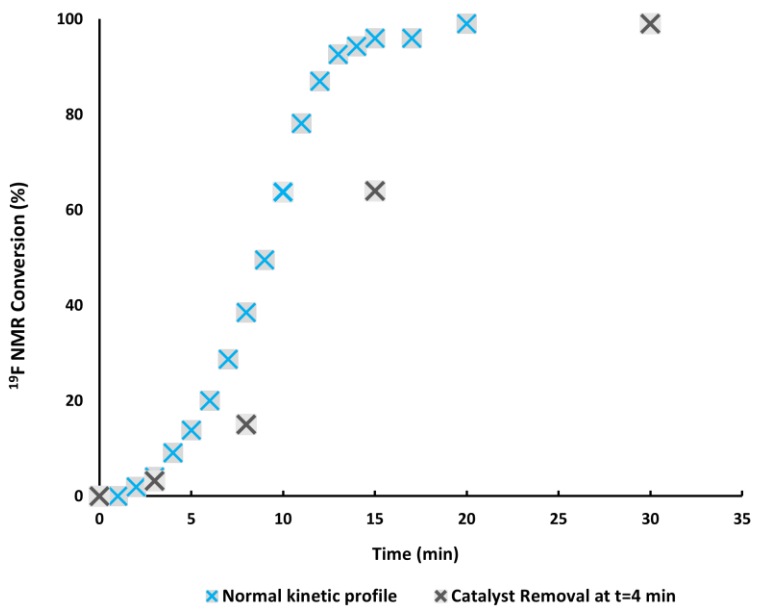

Figure S22 Kinetic profile of the model SMCC reaction (68 ppm Pd) when catalyst was filtered from reaction solution at t = 4 min. The reaction progress was analysed by ^{19}F NMR spectroscopy using 4,4'-difluoro-benzophenone as internal standard.

2.9.1 Quantification of palladium leached in solution, obtained from ICP analysis of the filtrate from a reaction catalyzed by immob-Pd_3Cl_2 3, stopped by filtration at 10 minutes (> 50 % conversion) reaction time.

The filtrate set aside in the experiment above that was terminated by filtration at 10 minutes reaction time was first evaporated to near dryness, then carefully dissolved in ~5 mL aqua regia with significant effervescence due to the high organic content. After agitation to disperse the washings these were transferred to three capped Teflon inserts for an EthosEasy microwave digester. These in turn were placed in a large vessel with deionised water and heated to 210 °C for 30 minutes. The resulting digested solution was transferred to at 25 mL volumetric flask and deionised water also used to wash thrice the inserts also transferred to the volumetric flask to ensure all traces of Pd would be transferred. The flask was made up to 25 mL before samples were analysed using an Agilent 5800 VDV ICP-OES Spectrometer, using the 229.651 and 340.458 nm lines. A blank and three standards (0.10, 0.50 and 1.00 ppm) were used to calibrate the instrument prior to analysis and a separately obtained 0.50 ppm check standard used during the experimental run. The sample was run in duplicate and gave 0.29 ppm in both runs, with an analysis error of < 5% of the value based on repeats of the calibration standards. Given the Pd amount in the reaction was 1.25 mg (10 mg of the Pd bound to the resin, with a 12.5 wt. % loading), leaching of 100% of the Pd into the reaction filtrate would correspond to 50 ppm. The measured value corresponds to 0.58 ± 0.03 % of the total palladium used in the SMCC reaction. It should be noted that this agrees well with the palladium on the resin sample in the transmission XAS samples remaining consistent in terms of the step-edge height before, during and after reaction within error (+/-3%).

2.10 Three-phase test procedure for standard Suzuki-Miyaura cross-coupling reaction catalyzed by both immob-Pd_3Cl_2 and Pd_3Cl_2 cluster

We prepared a polystyrene-bounded aryl iodide which was employed in SMCC reaction with soluble 4-anisyl boronic acid in the presence of immob-Pd_3Cl_2 and Pd_3Cl_2 separately, under the similar reaction conditions used in model SMCC reaction; the procedures are described as below.

2.10.1 Synthesis procedure for *N*-succinimidyl 4-iodo-benzoate[6]

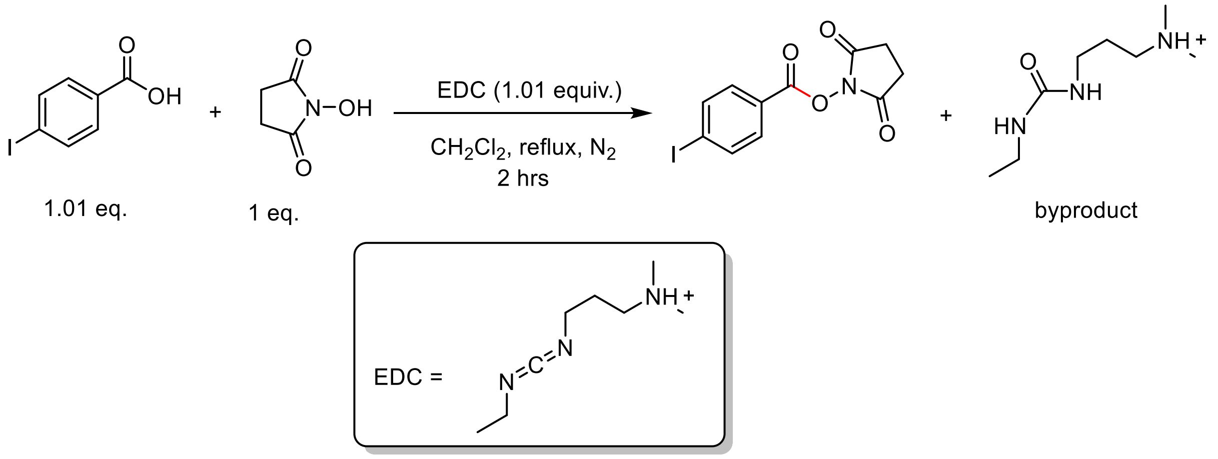

To an oven-dried three-neck round-bottom flask equipped with a stirrer bar and fitted with a condenser, 4-iodobenzoic acid (1.283 g, 5.18 mmol, 1.01 eq) and N-hydroxysuccinimide (590 mg, 5.12 mmol, 1eq.) were added and the system was purged with nitrogen. Dry, degassed dichloromethane (100 mL, 0.05 M) and EDC (986 mg, 5.18 mmol, 1.01 eq) was added to the flask and the mixture was subsequently heated at reflux temperature for 2 h under stirring. After completion, the reaction mixture was allowed to cool down and was washed with saturated sodium bicarbonate solution (3 × 15 mL) followed by water (3 × 15 mL) to remove byproduct. After washing, the organic layer was dried with $MgSO_4$ and concentrated in vacuo to dryness without further purification. The product was obtained as a white solid (1.050 g, 60% yield) and analysed by ^{1}H and ^{13}C NMR spectroscopy.

^{1}H NMR (500 MHz, $CDCl_3$): δ 7.91-7.87 (m, *J* = 8.70 Hz, 1.95, 2H, *aryl*-C**H**), 7.84-7.81 (m, *J* = 8.70, 1.95 Hz, 2H, *aryl*-C**H**), 2.91 (s, 4H, C**H**$_2$-C**H**$_2$). ^{13}C NMR (125 MHz, $CDCl_3$): δ 169.11 (s, *aryl*-**C**O), 161.68 (s, *alkyl*-**C**O), 138.4 (s, *aryl*-**C**h), 131.7 (s, *aryl*-**C**H), 124 (s, *aryl*-**C**-X), 103.4 (s, *aryl*-**C**-X), 25.7 (s, *alkyl*-**C**H$_2$). Melting point (°C): 227.1 – 232.1 (decomposed to black material).

Elemental analysis: measured (%) C 37.81, H 2.23, N 3.72, and remainder 56.35. This is comparable with theoretical values (%) C 38.29, H 2.34, N 4.06, and remainder 55.31.

2.10.2 Synthesis procedure for immobilized aminomethyl-polystyrene bound 4-iodo-benzoate

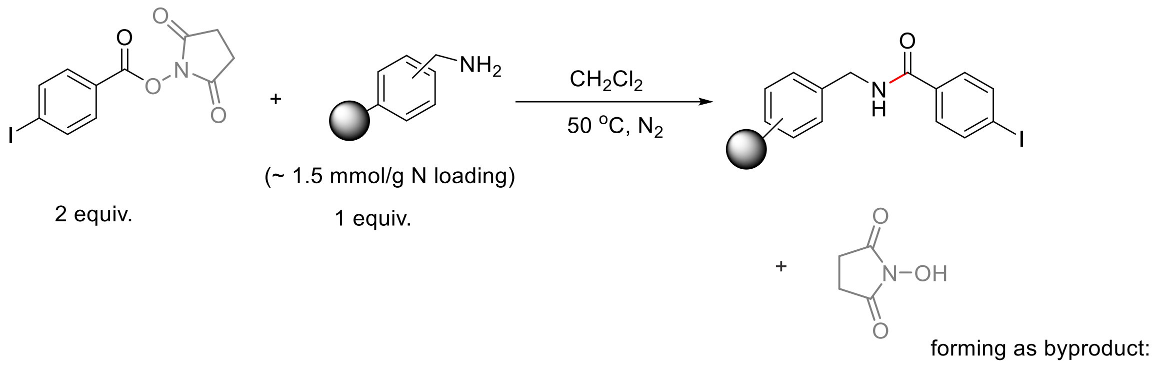

To an oven-dried septum-screw-cap vial, aminomethyl polystyrene resin (200-400 mesh) with ~ 1.5 mmol.g^{-1} amine loading (332.9 mg, 0.5 mmol, 1 eq.) and N-succinimidyl-4-iodobenzoate (345.1 mg, 1 mmol, 2 eq.) was added and purged with N_2 using a balloon. Dried and degassed methylene dichloride (3 mL) was added to the reaction vial via syringe and was placed in one of the positions in a 6-well plate thermomixer. The heterogenous mixture was heated up to 50 °C and set for shaking at 500 rpm overnight. The reaction proceeded heterogeneously and after completion, the mixture was exposed to the air and filtered through a sinter funnel. The filtrate solution, containing released N-hydroxysuccinimide as well as 1 equivalent excess *N*-succinimidyl-4-iodobenzoate, was concentrated to dryness (152.8 mg by mass, analysed by ^{1}H NMR spectroscopy) confirming conversion to be quantitative. The solid filter was washed with dichloromethane (3 × 10 mL) and diethyl ether (5 × 5 mL) and left under vacuum to dryness without further purification. The immobilized 4-iodo-benzoate was obtained as white solid (481.7 mg by mass) and analysed by ATR-IR spectroscopy. In ^{1}H NMR of the filtrate, the integration ratio of signals assigned to N-succinimidyl-4-iodobenzoate and released N-hydroxysuccinimide is 1:1 which confirmed 1 equivalent of the substrate was consumed to anchor to aminomethyl polystyrene resin, as shown in Figure S23.

IR [ν, ATR, soild-state, cm^{-1}]: 3024 (w), 2922 (m), 2851 (w), 1713 (w), 1645 (br, C=O), 1586 (m), 1492 (w), 1449 (w), 1005 (m), 748 (s), 695 (s), 530 (m).

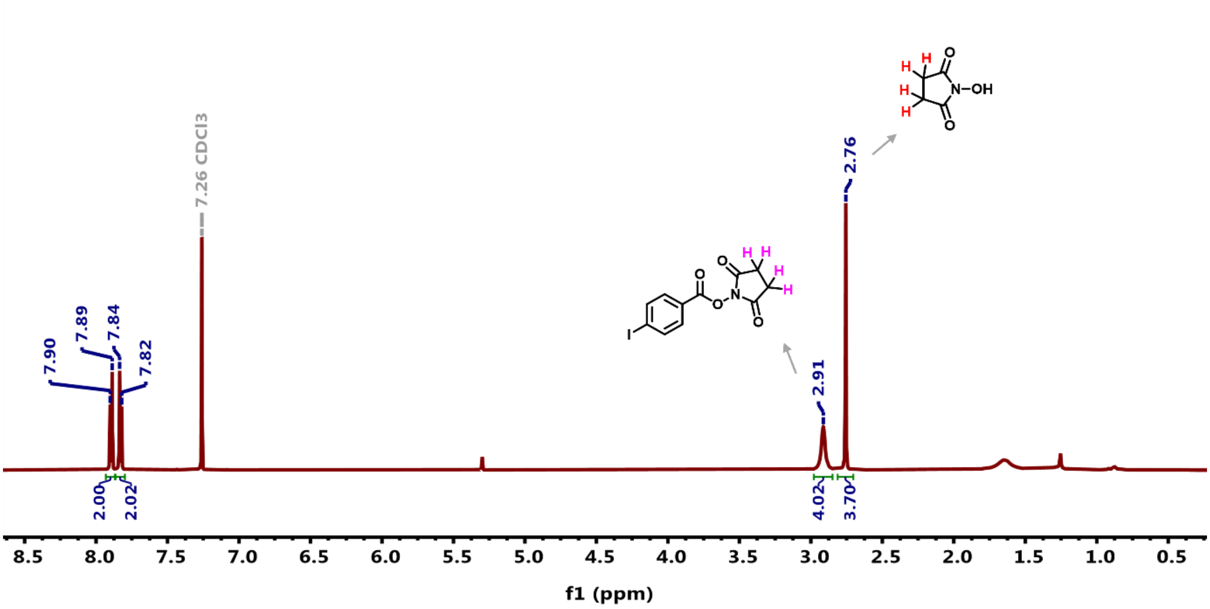

Figure S23 ^{1}H NMR spectrum of filtrate showing clean presence of released N-hydroxysuccinimide (1 eq.) and excess N-succinimidyl-4-iodobenzoate (1 eq.) with their integration ratio of 1:1 in $CDCl_3$.

2.10.3 General procedure for three-phase test experiment: SMCC reaction of immobilized aryl iodide and 4-anisyl boronic acid

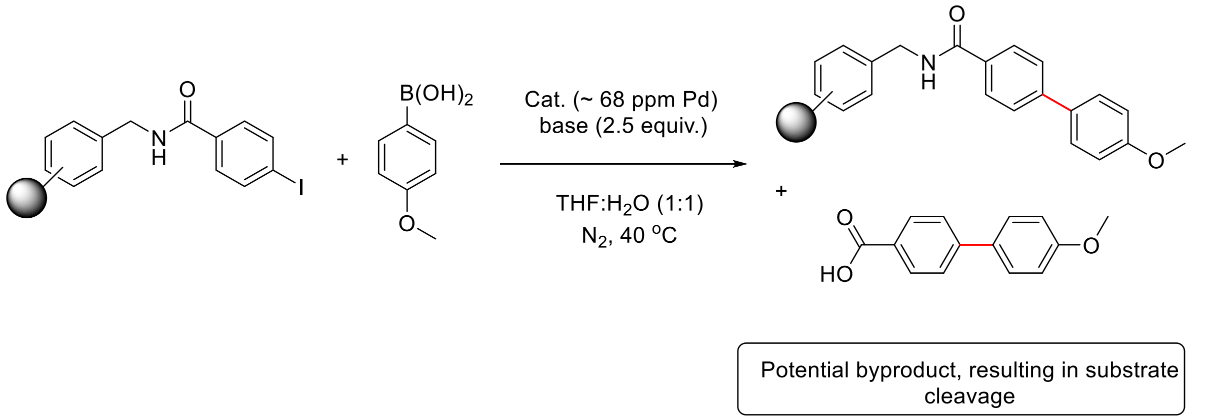

Catalyst	*Catalyst Mass /mg*	*Pd Amount /mmol*	*Equivalence of Pd:Substrate*	*Reference*
Immob-Pd_3Cl_2 base: nBu_4NOH	10	0.01 (Pd)	0.01:1	NEJ-3-277
Pd_3Cl_2 base: nBu_4NOH	6	0.01 (Pd)	0.01:1	NEJ-3-269
Immob-Pd_3Cl_2 base: K_2CO_3	10	0.01 (Pd)	0.01:1	NEJ-3-281

To an oven dried Schlenk flask equipped with a stirrer bar, immobilized 4-iodo-benzoate anchored to aminomethyl polystyrene (665 mg, 1 mmol, 1 eq.), 4-anisyl boronic acid (152 mg, 1 mmol, 1 eq.), Solid-supported Pd_3Cl_2 cluster (10 mg, 1 mol% Pd), trimethoxybenzene (84.1

mg, 0.5 mmol, 0.5 eq.) as internal standard were added and the reaction flask was evacuated and backfilled with N_2 three times. Dry, degassed THF (2.5 mL) was added via syringe to the reaction flask. A heterogenous mixture was formed as the reaction temperature reached 40 °C under stirring. To this solution, a degassed aqueous solution of tetra-*n*-butylammonium hydroxide (2.5 mL, 1.0 M) was added via syringe - *n*Bu_4NOH was sonicated for ca. 15 min before adding to the reaction mixture to avoid solidification (gelation) of the base during the transfer. The reaction was stirred at the set temperature for the specified time and samples were taken at certain time points. Samples were analysed ex situ via NMR spectroscopy, using the signals in the spectra for both soluble 4-anisyl boronic acid reactant and the internal standard to obtain the reaction conversion.

Since, 4-anisyl boronic acid is known to be in hydrolysis equilibrium with its boroxine (trimerization of free boronic acids) in presence of water,[7] signals attributed to 2,4,6-tris(4-methoxyphenyl)boroxine were seen as shown in Figure S24 as an example of the ^1H NMR spectrum from the three-phase test mixture after 1 h of reaction. By having the integrals of internal standard and the combined methoxy signals from unreacted boronic acid and boroxine together, the conversion to the cross-coupled product could be calculated Table S3.

Table S3 Calculation of conversion to cross-coupled product in the three-phase test reaction based on the proton integral peaks of internal standard, unreacted boronic acid and 2,4,6-tris(4-anisyl)boroxine in ^1H NMR spectrum of crude after 1 h.

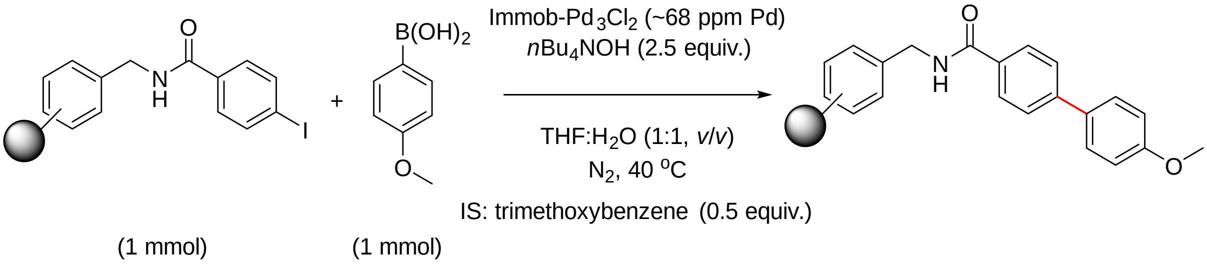

^1H NMR spectrum	*OCH_3 peak integral*	*Number of protons*	*amount/mmol*	*Conversion/%*
MeO-PhB(OH)$_2$ + Boroxine (unreacted)	4.99	3	<0.99 *(calculated)*	>99[a]
trimethoxybenzene (IS)	7.40	9	0.50 *(known)*	N/A
Cross-coupled (main Product)	N/A	N/A	<0.01	<1

[a] Unreacted 4-anisyl boronic acid and boroxine.

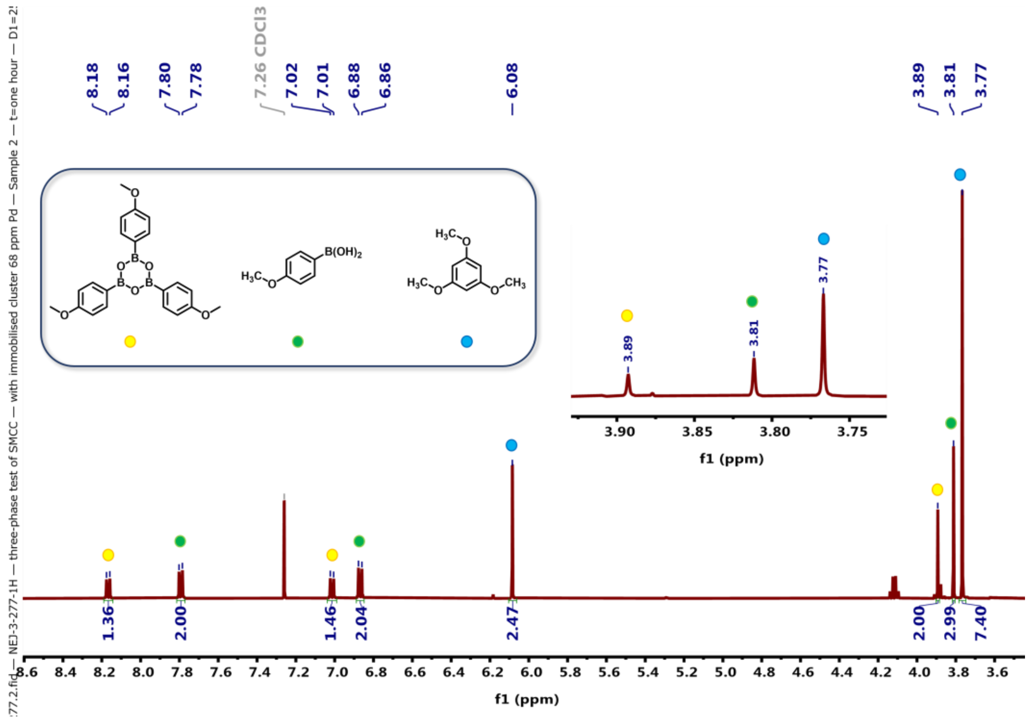

Figure S24 ^{1}H NMR spectrum of the crude reaction mixture from the three-phase test after 1 h – immob-Pd_3Cl_2-catalyzed SMCC reaction of immobilized 4-iodo-N-benzamide with soluble 4-anisyl boronic acid under the standard reaction conditions. Presence of signals assigned to boroxine product (yellow circle), internal standard (blue circle) and the remaining unreacted 4-anisyl boronic acid(green circle) and their integrals shown in the spectrum.

2.10.4 Validation test for three-phase test experiment

Since the substrate used in three-phase test contains an amine functional group, one could argue that the amine in the structure of the substrate could potentially poison the catalyst and inhibit the reaction progress, so the interpretation based on the reaction rate being sluggish may relate to catalyst poisoning rather than inaccessibility between the catalyst and substate. To test this, we checked the reactivity of immob-Pd_3Cl_2 in the SMCC reaction of 4-iodo-N-methylbenzenamide with aryl boronic acid under the same reaction conditions. The procedure for synthesis of 4-iodo-N-methylbenzenamide and its reaction in SMCC with 4-anisyl boronic acid is described below.

2.10.4.1 Synthesis procedure for formation of 4-iodo-N-methylbenzenamide[8,9]

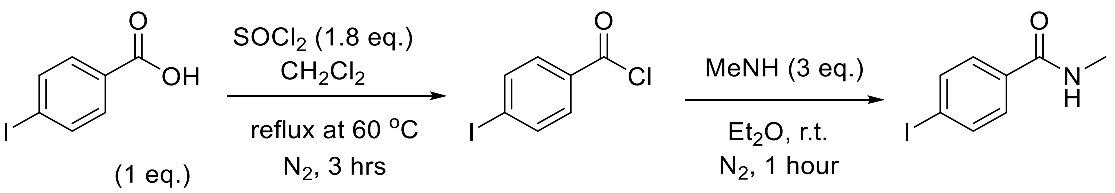

***i**. Synthesis of 4-iodobenzoic acid chloride*: to a three-neck round-bottom flask equipped with a stirrer bar and fitted with a condenser, $SOCl_2$ (3.6 mmol, 0.260 mL, 1.8 eq.) was added and evacuated and backfilled with N_2 three times. To a separate round-bottom flask equipped with

a stirrer bar, 4-iodo-benzoic acid (2 mmol, 496 mg, 1.0 eq.) was added and subsequently evacuated and backfilled with N_2 three times. Dried, degassed CH_2Cl_2 (5 mL, 0.4 M) was added to the flask containing 4-iodo-benzoic acid, which was stirred for 5 min. At this point solids were not completely solubilised and transferred dropwise via syringe to the round-bottom flask containing $SOCl_2$ under nitrogen and at room temperature. The resulting reaction mixture was heated to 60 °C under reflux and stirred for 3 h. After 0.5 h the reaction mixture became a homogeneous solution and a color change to bright yellow was observed. After the reaction time, the crude mixture was concentrated *in vacuo* and was directly used for the next step without further purification.

***ii**. Synthesis of 4-iodo-N-methylbenzamide*: to a round-bottom flask purged with N_2 and containing 4-iodobenzoic acid chloride that was formed in the previous step, degassed Et_2O (4mL, 0.5 M) was added and stirred for few minutes to form a homogenous solution. $MeNH_2$ in EtOH / 8M solution (6 mmol, 0.75 mL, 3 eq.) was then added to the reaction flask and the mixture was stirred at room temperature for 1 h. After completion, the crude mixture was washed with water and HCl (3 × 30 mL) and extracted by CH_2Cl_2 (3 × 10 mL) and dried over $MgSO_4$ The organic layer was concentrated in vacuo and resulted in a colourless solid product (292 mg by mass, 56% yield of product). The product was characterised by ^{1}H and ^{13}C NMR spectroscopy, ATR-IR and ESI-MS analysis.

^{1}H NMR (600 MHz, $CDCl_3$): δ 7.79-7.75 (m, *J* = 8.52, 1.95 Hz, 2H, *aryl*-C**H**), 7.49-7.46 (m, *J* = 8.52, 1.95 Hz, 2H, *aryl*-C**H**), 6.14 (s, N**H**), 3.01-2.98 (d, *J* = 4.87 Hz, 3H, HN-C**H**$_3$). ^{13}C NMR (150 MHz, $CDCl_3$): δ 167.5 (*aryl*-**C**O), 137.9 (*aryl*-**C**H), 134.1 (*aryl*-**C**-CO), 128.6 (*aryl*-**C**H), 98.4 (*aryl*-**C**I), 27.0 (*alkyl*-**C**H$_3$).

IR [ν, ATR, soild-state, cm^{-1}]: 3291 (s, N-H), 2936 (w), 2855 (w), 1631 (s, C=O), 1588 (s), 1551 (s), 1476 (s), 1398 (m), 1321 (s), 1301 (s, C-N), 1062 (m), 1001 (s), 840 (s), 744 (m), 709 (m), 705 (m), 648 (s), 487 (m), 452 (m). Melting point (°C): 163.4 – 167.1 (turned to yellow liquid).

ESI-MS Data (+ve mode) [M+H]: Found 261.9722 Calc. (for C_8H_8INO) 261.9728.

2.10.4.2 Procedure for validation test: SMCC reaction of 4-iodo-N-methylbenzamide with 4-anisyl boronic acid, catalyzed by immob-Pd_3Cl_2

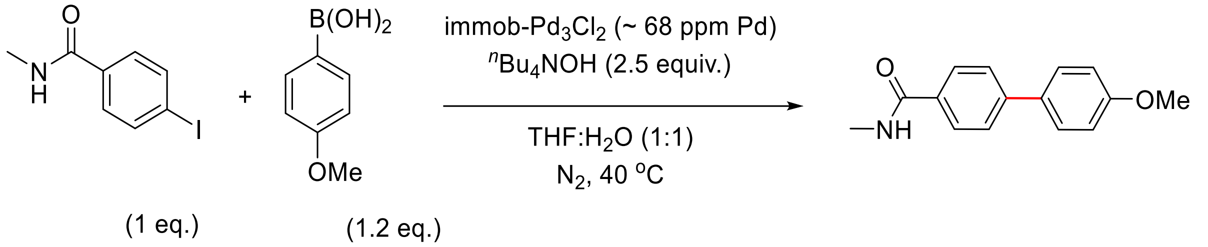

To an oven dried Schlenk flask equipped with a stirrer bar, 4-iodo-N-methylbenzoamide (130.5 mg, 0.5 mmol, 1 eq.), 4-anisyl boronic acid (91.2 mg, 0.6 mmol, 1.2 eq.), immob-Pd_3 cluster (5 mg, 1 mol% Pd), and 1,3,5-trimethoxybenzene (42.05 mg, 0.25 mmol, 0.5 eq.; internal standard) were added and the reaction flask was evacuated and backfilled with N_2 three times. Dry, degassed THF (1.25 mL) was added via syringe to the reaction flask. A heterogenous mixture was formed as the reaction temperature reached 40 °C under stirring. To this solution, a degassed aqueous solution of tetra-*n*-butylammonium hydroxide (1.25 mL, 1.0 M) was added via a syringe - *n*Bu_4NOH was sonicated for ca. 15 minutes before adding to the reaction mixture to avoid crystallisation of the base during the transfer. The reaction was stirred at the set temperature for over 24 h and samples were taken as indicated in the paper. After completion, the reaction mixture was allowed to cool down to room temperature and quenched with a saturated aqueous solution of NH_4Cl (5 mL). The crude reaction mixture was subsequently transferred to a separating funnel for the work-up process and extracted with EtOAc (3 × 5 mL). The extract solution was filtered through a celite plug (2 cm depth) before being concentrated in vacuo. The crude solid was purified by column chromatography on silica gel using Et_2O and EtOAc (90/10 v/v, $R_f = 0.21$) and a colourless solid was obtained in 74% yield (isolated product). The product was characterised by ^{1}H and ^{13}C NMR spectroscopy, ATR-IR and ESI-MS analysis.

^{1}H NMR (600 MHz, $CDCl_3$): δ 7.83-7.78 (m, *J* = 8.40, 1.90 Hz, 2H, *aryl*-C**H**), 7.62-7.58 (m, *J* = 8.40, 1.90 Hz, 2H, *aryl*-C**H**), 7.56-7.53 (m, *J* = 8.80, 2.14 Hz, 2H, *aryl*-C**H**), 7.00-6.96 (m, *J* = 8.80, 2.14 Hz, 2H, *aryl*-C**H**), 6.23 (br, 1H, N**H**), 3.86 (s, 3H, OC**H**3), 3.03 (d, *J* = 4.8 Hz, 3H, NH-C**H**3). ^{13}C NMR (150 MHz, $CDCl_3$): δ 167.9 (**C**=O), 159.7 (*aryl*-**C**-CO), 143.7 (*aryl*-**C**-OCH3), 132.6 (*aryl*-**C**-C-*aryl*), 132.4 (*aryl*-C-**C**-*aryl*), 128.2 (*aryl*-**C**H), 127.3 (*aryl*-**C**H), 126.7 (*aryl*-**C**H), 114.3 (*aryl*-**C**H), 55.4 (HN-**C**H3), 26.8 (O**C**H3). IR [ν, ATR, soild-state, cm^{-1}]: 3343 (m, NH), 2926 (br), 1633 (s, CO), 1602 (s), 1536 (s), 1490 (s), 1404 (s), 1287 (s), 1254 (m), 1203 (s), 1182 (m), 1152 (m), 1031 (m), 1024 (s), 1010 (m), 823 (s), 768 (s), 717 (s), 635 (w), 619 (w), 517 (s), 468 (s). Melting point (°C): 209.7–211.9 (decomposed to black material).

2.11 Procedure for testing the detachment of the Pd_3Cl_2 cluster from the resin support in the presence of excess (exogenous) triphenylphosphine ligand

An experiment was conducted to investigate potential release of Pd_3Cl_2 cluster from the resin support in the presence of excess triphenyl phosphine in dichloromethane as shown below.

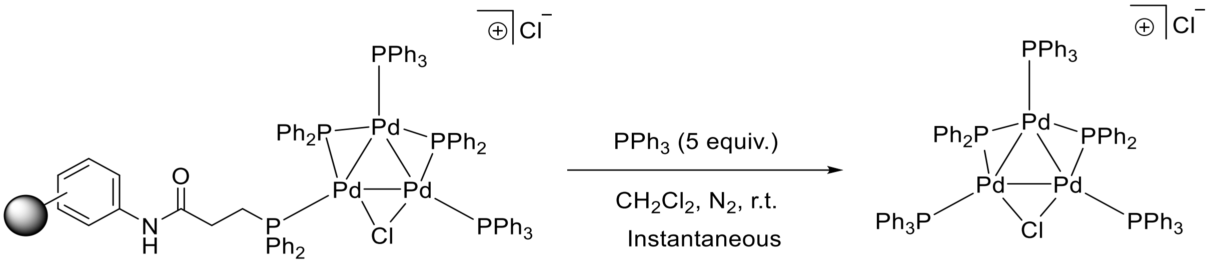

To an oven dried Schlenk flask equipped with a stirrer bar, immob-Pd_3 cluster (5 mg, 1 mol% Pd), 1,3,5-trimethoxybenzene (10 mg, 0.0039 mmol Pd, 1 eq.) and triphenylphosphine (5.12 mg, 0.0195 mmol, 5 eq.) were added and the reaction flask was evacuated and backfilled with N_2 three times. Dry, degassed CH_2Cl_2 (1.5 mL) was added via syringe to the reaction flask. A heterogenous mixture was formed upon stirring overnight. Samples that were taken from the reaction instantaneously (t = 0) and after 1 and 24 h were analysed by solution-phase ^{31}P NMR analysis. However, due to the low concentration of the formed product, the samples required a high number of scans, meaning that the reaction could continue proceeding while being NMR data was acquired, although the absence of agitation in the NMR tube may be expected to slow any further reaction.

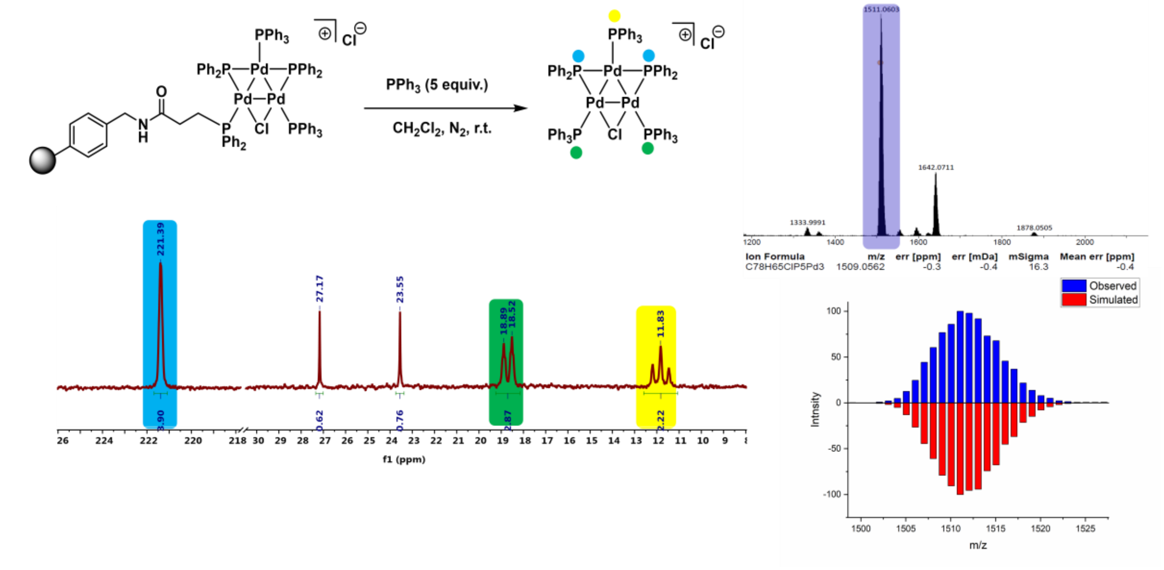

Figure S25 Reaction of immob-Pd_3Cl_2 catalyst with excess PPh_3; ^{31}P NMR spectrum of the reaction mixture, sample is taken instantaneously, and running under long scan by NMR (left) and presence of released Pd_3Cl_2 from support that is confirmed by ESI-MS and following the match of observed isotopic pattern of the signal at 1511 in ESI-MS with its simulated isotopic pattern (right).

2.12 Procedure for immobilized $[Pd_3(\mu\text{-}Cl)(\mu\text{-}PPh_2)_2(PPh_3)_3][Cl]$-catalyzed Suzuki-Miyaura cross-coupling reaction of 2,4-dibromopyridine and 4-fluoro-phenylboronic acid

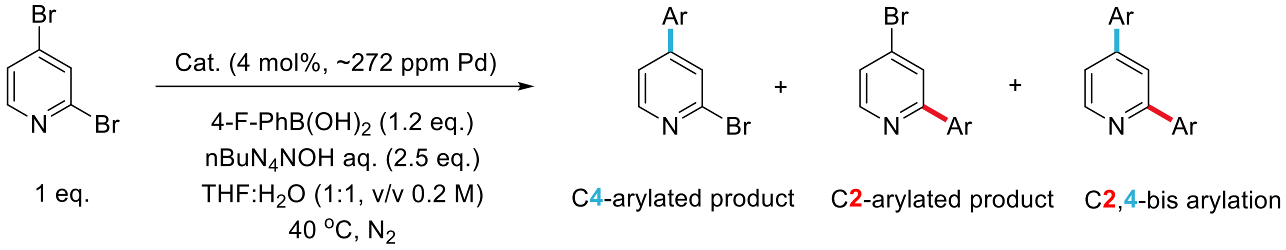

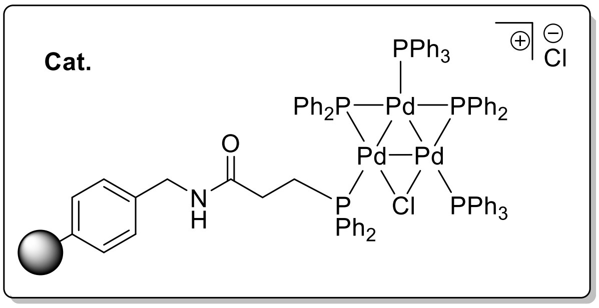

Ar: 4-F-Ph

To an oven-dried Schlenk flask equipped with a stirrer bar, 2,4-dibromopyridine (236.9 mg, 1.0 mmol, 1 eq.), 4-fluorophenyl boronic acid (1.2 mmol, 1.2 eq.), Pd catalyst (4 mol% Pd, 272 ppm Pd), 1,3,5-trimethoxybenzene (84 mg, 0.5 mmol, 0.5 eq.; internal standard) were added, and the reaction flask was evacuated and backfilled with N_2 three times. Dry, degassed THF (2.5 mL) was added via syringe to the reaction flask. A homogenous red solution was formed as the reaction temperature was reached to 40 °C under stirring. To this solution, base (2.5 eq.) was added; degassed aqueous solution of nBu$_4$NOH (2.5 mL, 1.0 M) was added via. a syringe - nBu$_4$NOH was sonicated for ca. 15 minutes before adding to the reaction mixture to avoid crystallisation of the base during the transfer. The reaction was allowed to stir at the set temperature for the specified time and samples were taken as indicated. Samples were quenched with saturated aqueous solution of NH_4Cl and subsequently transferred to a vial for the work-up process and extracting with EtOAc (3 × 1 mL). The extract solution was filtered through a celite plug (2 cm depth) before concentration in vacuo. ^{1}H NMR spectra were recorded from samples taken in different times and conversions obtained using the internal standards.

Table S4 SMCC conversion at different time intervals (obtained by ^{1}H NMR analysis).

	Conversion (%)			
Time / hours	C4-arylated product	C2-arylated product	C2,4-diarylated product	C4:C2
2	28	8	9	7:2
4	54	12	12	9:2
24	66	8	21	16:2

2.13 Synthesis replication of Pd_3Cl_2 cluster synthesis using procedure reported in Li *et al.* paper[10]

$PdCl_2$ (0.48 mmol, 85 mg) was dissolved in an aqueous solution of HCl (0.96 mmol, 0.6 mL from 1.6 M HCl) followed by addition of THF (10 mL) and triphenylphosphine (0.313 g, 1.2 mmol) forming a yellow solution. After vigorous stirring for 8 min, $NaBH_4$ (0.08 g, 2.1 mmol, dissolved in 5 mL of ethanol) was added, accompanied with sudden change of colour to brown/black - the reaction solution was kept under vigorous stirring for 60 min. Then centrifugation was performed to remove undissolved (excess) triphenylphosphine, and the remaining solution was evaporated to dryness in vacuo on a rotary evaporator. To remove sodium salts, the solid was redissolved in 10 mL of methylene chloride, washed with water, and then evaporated to dryness again. The remaining brown/black solid was extracted with ethanol several times, and the obtained ethanol solution was concentrated to dryness by roto-evaporator. The solid was dissolved in minimum ethanol, and the solution was added dropwise to hexane. The precipitate was collected by centrifugation, and the product (black colour) was obtained in 2% yield (mass 26 mg).

^{31}P NMR spectrum confirmed presence of Pd_3Cl_2 cluster forming through this procedure by appearance of signals at δ_P 11.62, 18.68, and 221.40 ppm (Figure S26), although the signal to noise ratio was poor and several singlets representing impurities appeared in the region δ_P 18 – 36 ppm, their identity unknown. ESI-MS analysis of the cluster after work-up also confirmed presence of Pd_3Cl_2 cluster. ESI (+ve mode) [M^+] of $Pd_3(\mu\text{-}Cl)(\mu\text{-}PPh_2)(PPh_3)_3]^+$: Found 1511.0590 Calc. (for $C_{78}H_{65}ClP_5Pd_3$) 1511.0576. No ESI-MS signal found at 1553.13 for $[Pd_3(\mu\text{-}Ph)(\mu\text{-}PPh_2)(PPh_3)]^+$ at any steps of the synthesis.

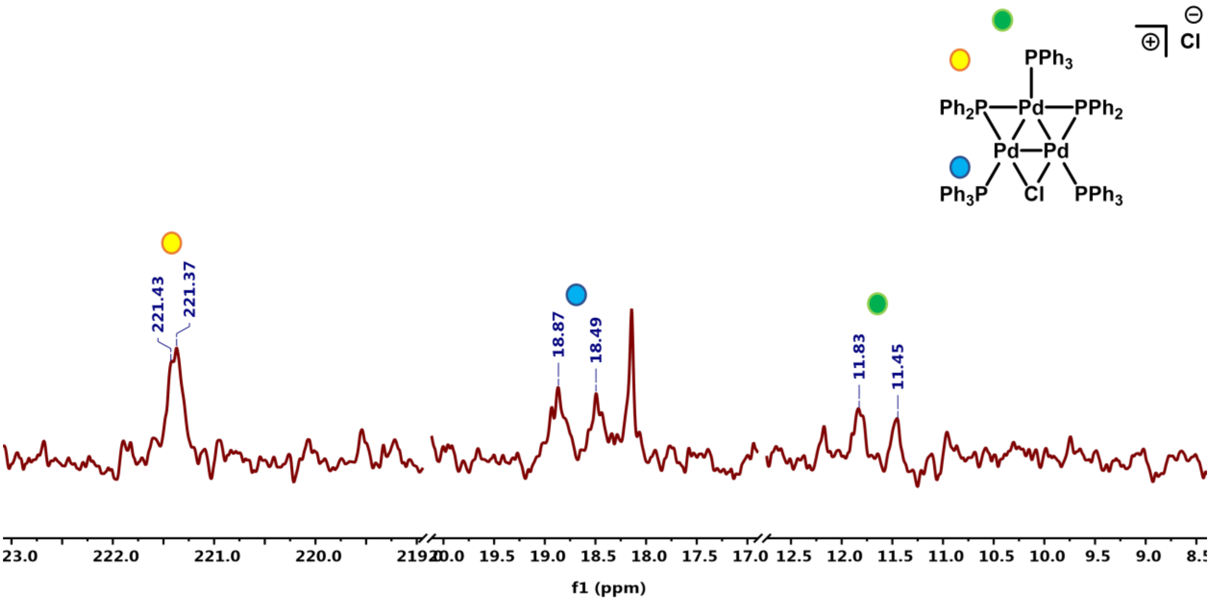

Figure S26 $^{31}P\{^{1}H\}$ NMR spectrum of the Pd_3Cl_2 (in CD_2Cl_2) synthesis through the procedure reported by Li *et al.*[9]

2.14 Detection of $[Pd_3(\mu\text{-}Ph)(\mu\text{-}PPh_2)(PPh_3)]^+$ in ESI-MS by analysing the post-reaction mixture of $Pd_3(OAc)_6$ and 6 equivalents of PPh_3 in THF

This procedure was carried out as previously published.[1] The $Pd(OAc)_2$ (2.3 mg, 0.01 mmol; 1 equivalent) and PPh_3 (5.2 mg, 0.02 mmol; 2 equivalents) were weighed and transferred into a J. Youngs NMR tube (in an Ar-filled Glove box). THF-d_8 (0.5 mL) was added, and the reaction was sealed and held at room temperature for 16 hours. After this time, an aliquot (10 µL) was taken and diluted in THF (1 mL), the sample was transferred into a vial which was sealed under an Ar atmosphere and analysed by ESI-mass spectrometry.

Figure S27 exhibits the measured and simulated isotopic distribution pattern of ESI-MS signals assigned to $[Pd_3(\mu\text{-}Ph)(\mu\text{-}PPh_2)(PPh_3)]^+$ and $[Pd_3(\mu\text{-}Br)(\mu\text{-}PPh_2)(PPh_3)]^+$ clusters. The two species have approximately similar mass-to-charge signals but distinctive isotopic distribution patterns.

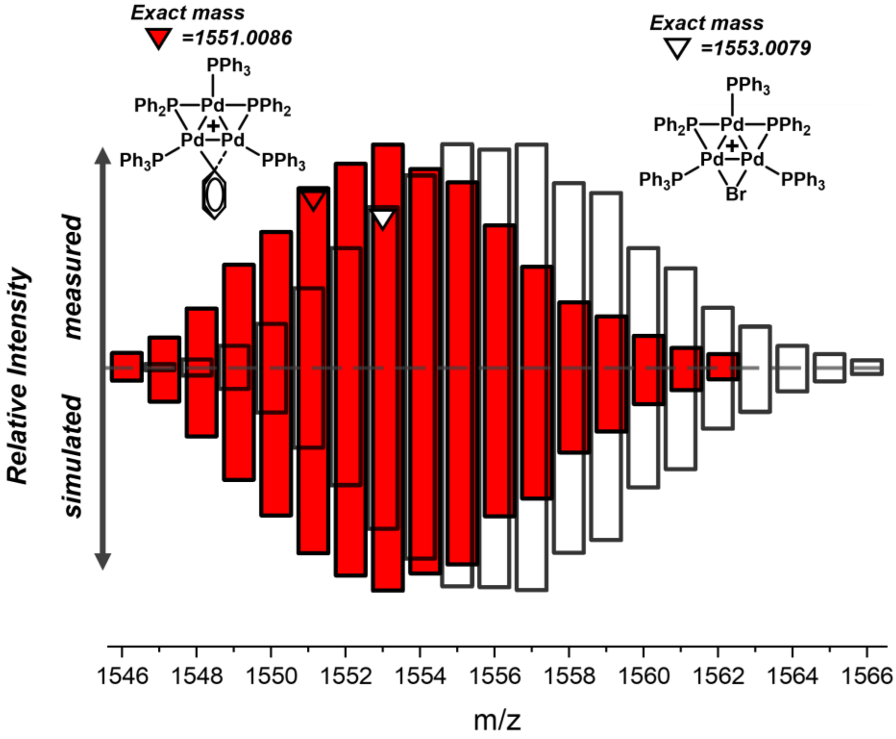

Figure S27 Highlighting differences in the m/z ratios and isotopic distributions of tripalladium cluster molecular cations Pd_3Ph^+ and Pd_3Br^+ (stack of relative intensity: measured vs. simulated). The Pd_3Ph^+ ion (red columns) was detected by analysing the post-reaction solution of $Pd(OAc)_2/2PPh_3$ by ESI-MS (16 hours reaction, room temperature in THF); shown alongside the Pd_3Br^+ ion (white columns) which was detected by analysing a THF solution of authentic $Pd_3Br.Br$ by ESI-MS.

2.15 Investigating Halide Ligation at the Immobilised-Pd_3Cl_2 Cluster Suzuki–Miyaura Catalysis

To an oven dried Schlenk flask equipped with a stirrer bar, 4-bromo-1-fluorobenzene (1.0 mmol, 110 μL, 1 eq.), 4-anisyl boronic acid(1.2 mmol, 182.35 mg, 1.2 eq.), solid-supported Pd_3 cluster (10 mg, 1 mol% Pd), trimethoxybenzene (84.1 mg, 0.5 mmol, 0.5 eq.; internal standard) were added and the reaction Schlenk flask was evacuated and backfilled with N_2 three times. Dry, degassed THF (2.5 mL) was added via syringe to the reaction flask. The resulting solution was placed in a 40 °C oil bath. To this solution, a degassed aqueous solution of tetra-*n*-butylammonium hydroxide (2.5 mL, 1.0 M) was added via a syringe, initiating the Suzuki–Miyaura reaction. The reaction was stirred at 40 °C for 10 minutes after which point the reaction solution was separated from the solid-supported Pd_3 cluster by cannula filtration, taking *ca*. 45 seconds to complete the transfer of filtrate to a second Schlenk flask (under N_2, previously evacuated and backfilled three times). The filtrate was set aside for ICP-MS analysis (see page S78 for further information and discussion). The filter contents (including the cannula filter tip) were washed with THF (2.5 mL degassed) followed by H_2O (2.5 mL, degassed) and then again with THF (2.5 mL, degassed) (A. Figure 1). PPh_3 (26.2 mg, 0.1 mmol; 10 eq. per Pd) was weighed onto a fresh oven dried Schlenk tub which was evacuated and backfilled with N_2. CH_2Cl_2 (dry,

degassed) was added, and the resulting solution was added to the Schlenk tube containing the reaction filter contents, stirring for 1 hour at 23 °C (B. and C. Figure S28).

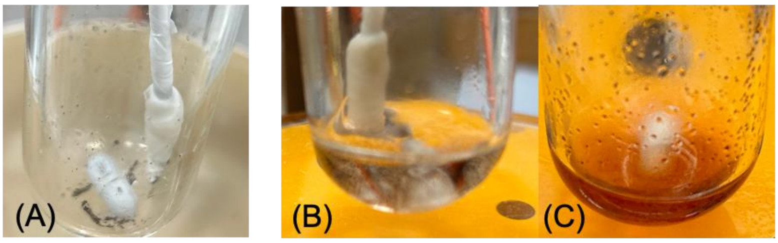

Figure S28 Images of the reaction Schlenk contents: (A) solid material after filtering and washing the reaction solution, (B) ca. two minutes after addition of PPh_3 in CH_2Cl_2 solution, and (C) the solution after one hour of stirring at 23 °C.

A sample of this solution was analysed by ESI-MS which showed presence of $[Pd_3Cl]^+$ and $[Pd_3Br]^+$ ions (Figure S29), giving evidence that the immobilised cluster is to an extent converted from the Pd_3Cl^+ to Pd_3Br^+ under the working catalytic conditions.

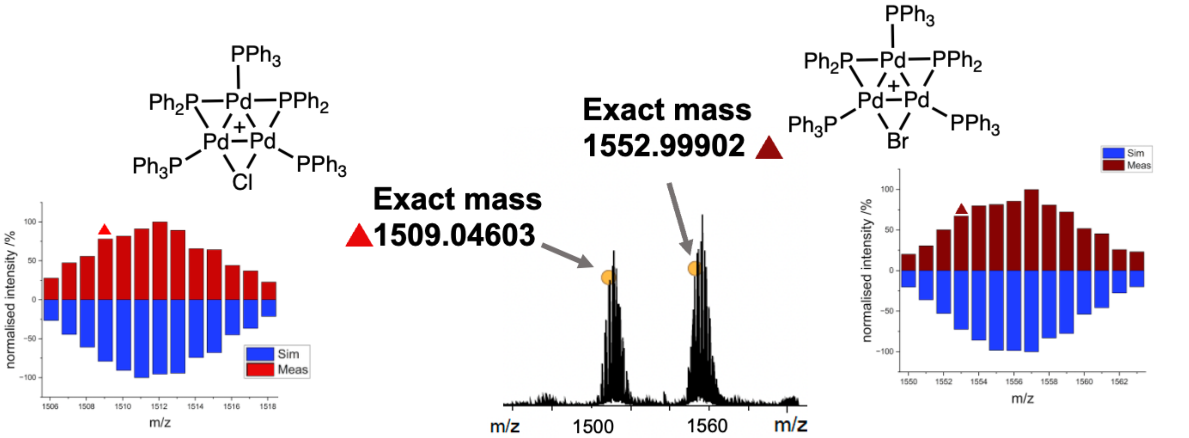

Meas. m/z	#	Ion Formula	m/z	err [ppm]	err [mDa]	mSigma	Mean err [ppm]
1509.046026	1	C78H65ClP5Pd3	1509.059965	6.7	10.2	61.9	6.6
1552.999022	1	C78H65BrP5Pd3	1553.009154	4.3	6.7	61.2	5.6

Figure S29 ESI-MS analysis of the PPh_3 solution extract from immob-Pd_3Cl_2 cluster which was filtered off 10 minutes into Suzuki-Miyaura catalytic turnover.

XAS details of analysis

2.16 Experimental and analysis procedures for XAS data.

XAS experiments were performed at beamline B18, Diamond Light Source, Oxfordshire, UK. A double-crystal Si(311) monochromator was used to scan X-ray energy from -200 to 830 eV relative to Pd K-edge (24,350 eV). Samples were prepared as described from section 2.16.1 to section 2.16.4. The reference standards were selected to include combinations of bromide, phosphines and nBu_4N ligands, the latter from the base used in reaction, and these have largely not been reported previously. $PdBr_2$ is consistent with spectra reported in the literature.[11] The samples were employed for fluorescence or transmission XAS measurements depending on the concentration of the sample. For fluorescence measurements it was not consistently possible to measure a pure Pd metal foil concurrently behind the sample due to the experimental geometry, but the energy scales were noted to be invariant over many hours within < 0.5 eV, and checked intermittently through the measurements with Pd foil samples. The energy scale was corrected using the first maximum in the derivative of the Pd foil spectrum (24,350 eV).

XAS data processing was performed using IFEFFIT[12] with the Horae package[13] (Athena and Artemis). The amplitude reduction factor, S_0^2 was derived from EXAFS data analysis of the Pd foil reference spectrum (for which the co-ordination numbers of the fcc metal are known), yielding a value of 0.80±0.04 or 0.85±0.04 (depending on which of two beamtime sessions data was taken from, although within error). This was then fixed in the analysis of sample spectra. The parameters corresponding to the correction to the photoelectron energy origin, bond lengths, and mean-squared relative deviation of atoms around absorbing atoms were then varied during fitting. Structures based on the Pd_3Cl_2 cluster with a bridging chlorine, bromine or aryl group were considered. In each case the first shell Pd, P and halogen or carbon were used as possible contributions for first shell fits. Given the similarity of the bond lengths in available X-ray crystal structures (see Section 4) for the Pd-PPh_2 and Pd-PPh_3, the number of variables was kept to a minimum by fitting only one Pd-P distance. All fits were performed using multiple k-weight fitting, although k^3-weighted data is shown in figures. Improved fits of the reference metal foil were obtained by fitting to higher values of R using a model accounting for multiple scattering paths (those regarded significant in Feff) up to 5.5 Å effective path length, and using the further physically reasonable assumption that all Pd-Pd interactions grow or shrink by a constant scaling factor relative to the known bulk Pd fcc metal crystal. Assumed relations of co-ordination number and mean-squared relative deviation are tabulated in Table S5. PCA and Least squares fitting was conducted using the inbuilt package within the Athena software. PCA of the XANES region was based on data 20 eV below the edge and 60 eV above

the edge and used the normalised $\mu(E)$ data, k-space analysis was performed between $k = 3$ and $k = 12$, and this region only was Fourier transformed to return the representative R-space plots given.

Table S5 Assumed relations of co-ordination number and mean-squared relative deviation used in extended Pd fits (based on fcc structure).

Scattering path	*Co-ordination number*	σ^2
Pd1 Single scattering	12	σ^2(Pd1)
Pd2 Single scattering	6	σ^2(Pd2)
Pd1Pd1 Acute triangle	48[a]	2 × σ^2(Pd1)
Pd1Pd2 Other double scattering	48	σ^2(Pd1)+ σ^2(Pd2)
Pd3 Single scattering	24	σ^2(Pd3)
Pd1Pd1 Obtuse triangle	48 [a]	2 × σ^2(Pd1)
Pd1Pd3 Obtuse triangle	96	σ^2(Pd1)+ σ^2(Pd3)
Pd4 Single scattering	12	σ^2(Pd4)
Pd1Pd1 Non-forward linear	12 [a]	2 × σ^2(Pd1)
Pd1Pd4 Forward scattering	24	σ^2(Pd4)
Pd1 Forward through absorber	12	2 × σ^2(Pd1)
Pd1Pd4Pd1 Double forward scattering	12	σ^2(Pd4)

[a]To minimise the number of independent parameters the approximation is made that $CN_{Pd1xPd1}$ can be used when fitting all three of these paths involving two nearest shell Pd scatterers and the 4:4:1 ratio maintained.

2.16.1 General procedure for preparation of reaction samples of immob-Pd_3Cl_2 for EXAFS/XANES analysis

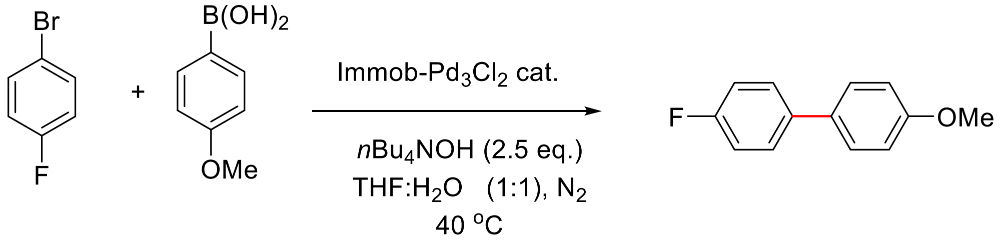

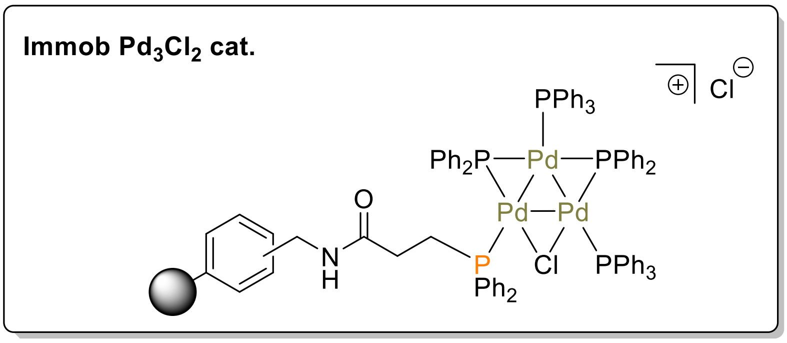

To an oven dried Schlenk flask equipped with a stirrer bar, 4-bromo-1-fluorobenzene (1.0 mmol, 110 µL, 1 eq.), 4-anisyl boronic acid(1.2 mmol, 182.35 mg, 1.2 eq.), and solid-supported Pd_3 cluster (10 mg, 1 mol% Pd) was added and the reaction flask was evacuated and backfilled

with N_2 three times. Dry, degassed THF (2.5 mL) was added via syringe to the reaction flask. A heterogenous mixture was formed as the reaction temperature reached 40 °C under stirring. To this solution, a degassed aqueous solution of tetra-*n*-butylammonium hydroxide (2.5 mL, 1.0 M) was added via a syringe - nBu$_4$NOH was sonicated for ca. 15 min before adding to the reaction mixture to avoid crystallisation of the base during the transfer. The reaction was stirred at the set temperature for the specified time (separately 5 and 40 min). At these specific times, the reaction was stopped, and the catalyst was separated from reaction solution by canula filtration. The catalyst was dried over vacuum, ground to a fine homogeneous powder using a mortar and pestle (grinding was undertaken with care and slowly to avoid generating the mechanical heat) and later transferred to cryogenic storage vials to be analysed using ex situ XAS. The filtrate reaction solution after catalyst filtration was also concentrated to dryness, ground up to fine homogeneous powders using mortar and pestle and later transferred to cryogenic storage vials for further XAS analysis of potential leached Pd species.

2.16.2 Procedure for preparation of standard reference samples for EXAFS/XANES study of immob-Pd$_3$Cl$_2$ experiments

Reference samples: immob-Pd$_3$Cl$_2$ (*in-house* synthesised – see section 2.3), $PdBr_2$ (commercially available), and [nBu$_4$N]$_2$[Pd$_2$Br$_6$] (*in-house* synthesised – *vide infra*)

General: The standard reference Pd were mixed with 200 mg of cellulose and ground in a pestle and mortar for formation of homogeneous solid powder. The samples were put in Nalgene cryovials for XAS analysis. The content of Pd in the samples was set to 1 wt.%.

Procedure for synthesis of [nBu$_4$N]$_2$[Pd$_2$Br$_6$] reference sample

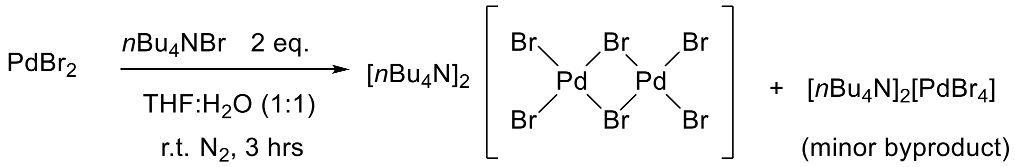

To an oven dried Schlenk flask equipped with a stirrer bar, $PdBr_2$ (0.15 mmol, 40 mg, 1 eq.) and nBu$_4$NBr (0.3 mmol, 96.7 mg, 2 eq.) were added and the reaction flask was evacuated and backfilled with N_2 three times. Degassed THF (1.5 mL) and degassed H_2O (1.5 mL) were added via syringe to the reaction flask. A heterogenous brown mixture was formed on addition of solvents. The reaction was stirred under N_2 for 3 h at room temperature until completion. The reddish-brown heterogenous mixture was allowed to stand until precipitated and then the solvent was removed via canula filtration. The brown solid was dissolved in THF (3 mL) and

set for crystallisation by layering with hexane, for further purification. The product was obtained in 49 mg (71% yield) as a brown solid. ^{1}H NMR (500 MHz, 128 scans, $CDCl_3$): δ 3.62-3.56 (m, 8H, $CH_3CH_2CH_2\boldsymbol{CH_2}N$), δ 1.88-1.78 (m, 8H, $CH_3CH_2\boldsymbol{CH_2}CH_2N$), δ 1.64-1.56 (sep, 8H, $CH_3\boldsymbol{CH_2}CH_2CH_2N$), δ 1.09-1.03 (t, 12H, $\boldsymbol{CH_3}CH_2CH_2CH_2N$). Far-IR [ν, cm^{-1}]: 244.91, 254.6 (bridging Pd-Br). Elemental analysis for $C_{32}H_{72}Br_6N_2Pd_2$: Found C, 33.38 H, 6.36 N, 1.56; Calc. C, 32.65 H, 6.17 N, 2.38.

2.16.3 Procedure for preparation of reaction samples of Pd_3Cl_2 for EXAFS/XANES analysis

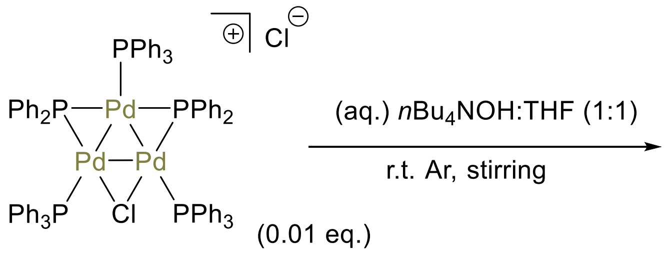

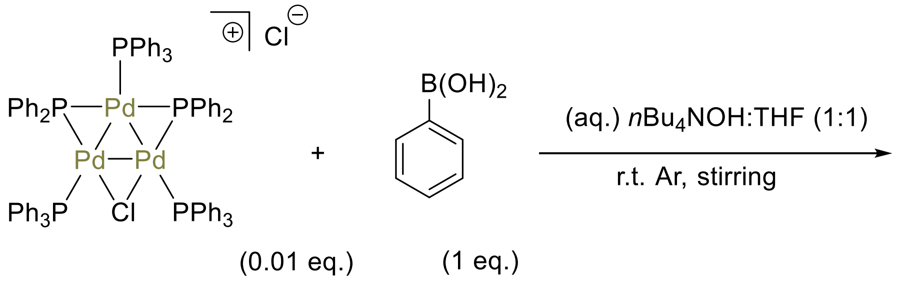

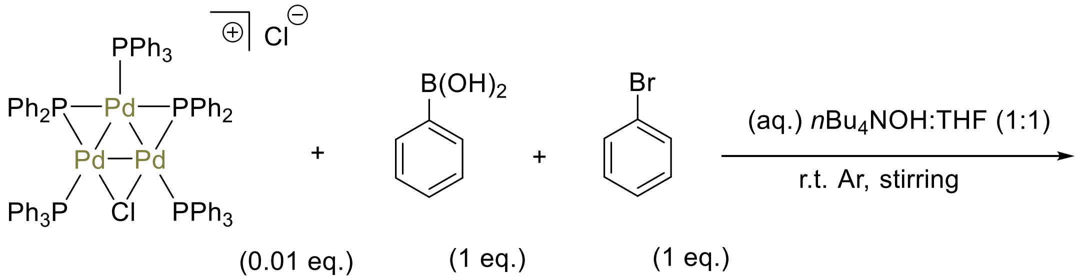

To an oven dried Schlenk flask equipped with a stirrer bar, Pd_3Cl_2 (18 mg, 0.012 mmol) was added, and the flask was evacuated and backfilled with Ar three times. Dried and degassed THF (1 mL) and degassed *n*Bu_4NOH (1 mL) were added to the reaction flask via syringe while stirring. In another Schlenk flask, a Nalgene cryovial was with no cap was placed and the flask purged with argon under higher pressures of Ar. The content of the flask containing Pd_3Cl_2, THF and aqueous *n*Bu_4NOH was transferred via syringe to the Nalgene cryovial within the

Schlenk flask while purging with Ar. The cryovial was lifted from the flask using a hook and the cap was placed while being purged under argon (Figure S30). The Nalgene cryovial was placed within a liquid N_2 Dewar and transferred to the XAS instrument for analysis.

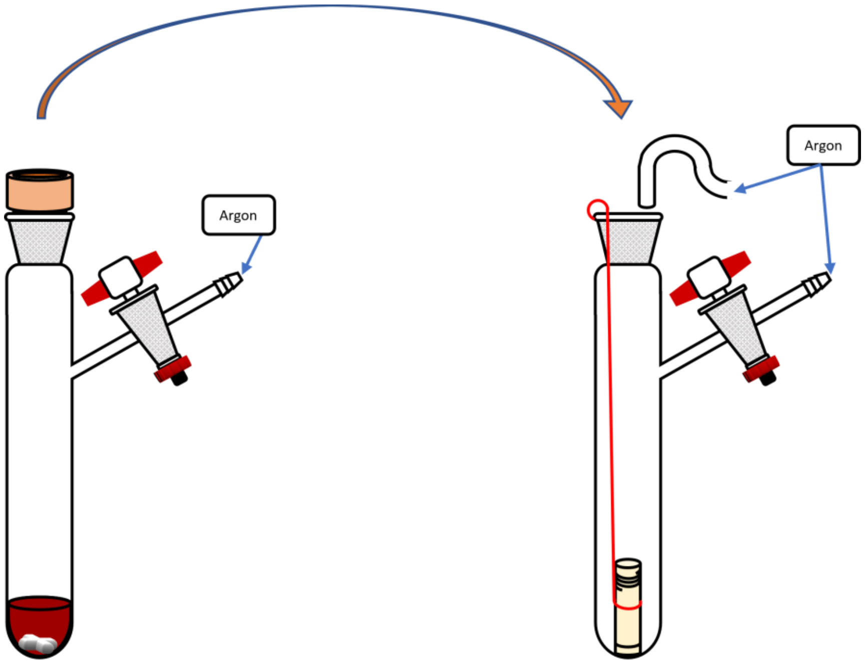

Figure S30 A diagram showing the process of making reaction solution in a separate Schlenk flask under argon and transferring them to a Nalgene cryovial within a Schlenk flask that is under active purge of Ar. The cryovial has lifted using a hook for placing the cap and subsequently cooling down to -195 °C by placing it in a liquid N_2 Dewar.

Another Schlenk flask equipped with a magnetic stirrer bar was charged with Pd_3Cl_2 (18 mg, 0.012 mmol) and $PhB(OH)_2$ (0.328 mmol, 40.0 mg, 1 eq.) and the flask was evacuated and backfilled with Ar three times. Dried and degassed THF (1 mL) and degassed $n$$Bu_4NOH$ (1 mL) were added to the reaction flask via syringe forming a black homogenous solution which was stirred for 20 min. In another Schlenk flask, a Nalgene cryovial was placed and purged with a strong flow of argon. The black homogenous solution was transferred to the Nalgene cryovial in the flask via syringe and the sample was lifted using a hook to screw the cap and placed in a Dewar of LN_2 to cool down to -195 °C (the similar process as shown in Figure S30) for XAS analysis. Another Schlenk flask equipped with stirrer bar was charged with Pd_3Cl_2 (18 mg, 0.012 mmol) and $PhB(OH)_2$ (0.328 mmol, 40.0 mg, 1 eq.) and the flask was evacuated and backfilled with Ar three times. Bromobenzene (37 µL, 55.11 mg, 0.352 mmol, 1 eq.), dried and degassed THF (1 mL) and degassed $n$$Bu_4NOH$ (1 mL) were added to the reaction flask via syringe forming a black homogenous solution of similar appearance to before, which was stirred for 20 min. In another Schlenk flask, a Nalgene cryovial was placed and purged with strong flow of argon. The black homogenous solution was transferred to the Nalgene cryovial

in the flask via syringe and the sample was lifted using a hook to screw the cap and placed in a dewar of LN_2 to cool down to -195 °C (the similar process as shown in Figure S30) for XAS analysis.

2.16.4 Procedure for preparation of standard reference samples for EXAFS/XANES study for Pd_3Cl_2 experiments

Standard reference samples: Pd_3Cl_2 (*in-house* synthesised – see section 2.1), $Pd(PPh_3)_3$ (*in-house* synthesised – *vide infra*), $[nBu_4N]_2[Pd_2Br_6]$ (*in-house* synthesised – see section 0), $[nBu_4N]_2[PdCl_4]$ (*in-house* synthesised – *vide infra*), and H_2PdCl_4 (commercially available)

General: In an oven dried Schlenk flask equipped with stirrer bar, standard reference Pd was added, and the flask evacuated and backfilled with Ar three times. Dried and degassed THF (2 mL) was added to the flask via syringe under stirring. The content of Pd in reference samples was set to 500 ppm. A separate Schlenk flask containing a Nalgene cryovial was purged with a strong flow of argon. The standard reference solution was transferred via syringe to the Nalgene cryovial under argon and subsequently the vial was sealed and cooled down to LN_2 temperature for XAS analysis (as described in Figure S30).

Procedure for formation of $Pd(PPh_3)_3$ reference sample

Adapted procedure from literature[1]: $Pd(OAc)_2$ (100.0 mg, 0.445 mmol) and PPh_3 (467.3 mg, 1.782 mmol) were added to an oven-dried Schlenk flask, which was evacuated and backfilled with N_2 (three times). THF (5 mL; degassed) and H_2O (0.05 mL) was added *via* syringe, the reaction temperature was raised to reflux (66 °C; cold finger condenser) and stirred for 18 h under flow of N_2. The reaction mixture was then allowed to cool to room temperature and filtered to remove a small amount of formed Pd black. The filtrate was then crystallised by careful layering with hexane (20 mL) and storing at –18 °C. After approximately 24 h of storage at –18 °C, the mother liquor was separated by filtration and the residual bright yellow solid (filter) was washed with Et_2O (3 x 2 mL). This residue was dried in vacuo overnight (ca. 0.02 mbar). The product was obtained as bright yellow crystals (190 mg, 48 %). Product formation was confirmed by ^{31}P and ^{1}H NMR spectroscopic analysis.

^{1}H NMR (500 MHz, Benzene-d_6) δ 7.49 (app. d, J = 7.4 Hz, 18H), 7.00 – 6.93 (m, 9H), 6.93 – 6.84 (m, 18H).^{31}P NMR (203 MHz, Benzene-d_6) δ 24.17.

Procedure for formation of $[nBu_4N]_2[PdCl_4]$ reference sample

Synthesised according to an established literature procedure.[14] In a Schlenk tube, to a stirred suspension of $PdCl_2$ (177.3 mg, 1.0 mmol) in CH_2Cl_2 (10 mL), a solution of n-Bu_4NCl (555.8, 2.0 mmol) was added *via* a syringe. The subsequent reaction mixture was magnetically stirred at room temperature for 18 hours, after which time the solvent was removed in vacuo and the

residue was triturated with Et_2O (10 mL), forming a brick-red solid which was separated by filtration and dried overnight *in vacuo* (*ca.* 0.02 mbar). The product **[*n*-Bu$_4$N]$_2$[PdCl$_4$]** appeared as a brick-red powder (524.4 mg; 71% yield). ^{1}H NMR (600 MHz, Chloroform-*d*) δ 3.57 – 3.51 (m, 8H; NC**H**$_2$), 1.81 – 1.72 (m, 8H; NCH$_2$C**H**$_2$), 1.54 (p, *J* = 7.4 Hz, 8H; NCH$_2$CH$_2$C**H**$_2$), 1.02 (t, *J* = 7.3 Hz, 12H; NCH$_2$CH$_2$CH$_2$C**H**$_2$). ^{13}C NMR (151 MHz, Chloroform-*d*) δ 59.43 (N*C*H$_2$), 24.58 (NCH$_2$*C*H$_2$), 20.00 (NCH$_2$CH$_2$*C*H$_2$), 14.00 (NCH$_2$CH$_2$CH$_2$*C*H$_3$). IR (ν, ATR, solid state, cm^{-1}) 2958 (s), 2934 (m), 2872 (m), 1476 (m), 1380 (w), 1152 (w), 1057 (w), 1028 (w), 881 (m), 793 (w), 736 (m).

2.17 XAS data analysis and discussion.

This section provides additional XAS data, processing and fitting information and PCA plots of the XAS data. For plots of data where spectra have been offset for clarity, XANES spectra are normalised between 0 and 1 prior to a fixed *y*-offset, EXAFS oscillations are centred about *y*=0, or EXAFS *R*-space magnitudes have a baseline of *y*=0.

In addition this section starts with a discussion of the use of XANES and EXAFS with PCA to speculatively assign the fate of palladium based on the spectra of the species in the concentrated filtrate of reactions of immob-Pd_3Cl_2 **3** and the end species of the stepwise interaction of Pd_3Cl_2 **1** with reaction species.

Within PCA, target transforms can be used to identify if a particular standard is a possible constituent of the data ensemble used in the PCA (or contains common components). Using the PCA model derived from the XANES spectra in Figure 9a, a range of possible standards were tested using this approach (Figure S35). While most are clearly not a constituent of the dataset used in the PCA, $Pd(PPh_3)_3$ dissolved in THF showed as a match. However, attempts to use this reference in linear combination fitting show it is not consistent with the species in the filtrate sample (*i.e.* it does not fully account for the data). Closer examination of the corresponding R-space EXAFS data for $Pd(PPh_3)_3$ in THF solution also highlights that upon dissolution this reference compound undergoes changes to Pd speciation which are more complicated than simply solvent co-ordination (*i.e.* it cannot be fitted by a simple molecular model of $Pd(PPh_3)_3$). The R-space data has a strong feature at ~2.4 Å (Figure S32). This is too large a distance to be attributable to either P, halide (X) or O nearest neighbors, and too intense to be light atoms (such as secondary shells of carbon scatterers – this was confirmed by unsuccessful attempts to fit this feature by interactions with the benzene ring of triphenylphosphine with the Pd such as are seen in linear Pd_3 chains.[15] Both $Pd(PPh_3)_3$ in THF solution and the species in the filtrate present at the end of reaction with immob-Pd_3Cl_2 **3** or

Pd_3Cl_2 **1** contain a mix of Pd-Pd and Pd-P interactions – some form of mixture of $Pd_x(PPh_3)_y$ species. The increase in the edge position in the filtrate samples (~24353-4 eV) also indicates a change of the Pd oxidation state to be more comparable to Pd(II) than the lower valent Pd_3Cl_2 **1** cluster, $Pd(PPh_3)_3$ in THF or Pd metal foil (~24350-1eV).

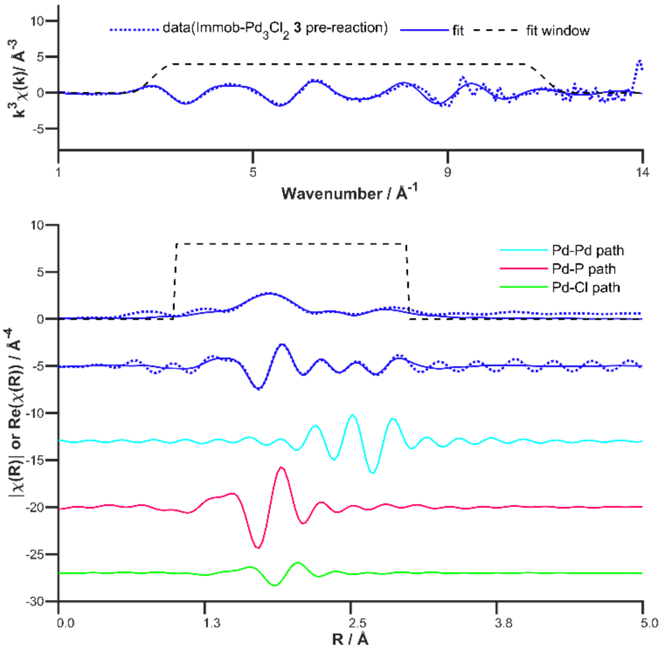

Figure S31 Immobilized-[Pd_3Cl_2] cluster prior to reaction, Pd K-edge EXAFS spectroscopy fitted data.

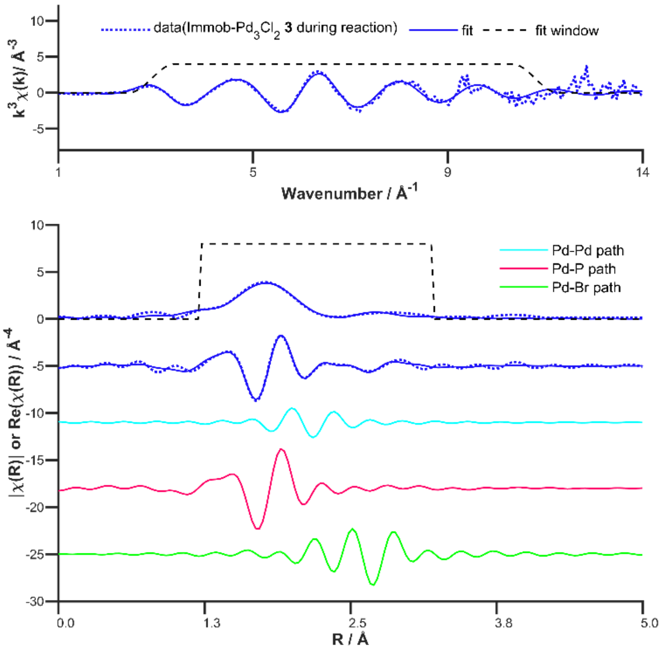

Figure S32 Immobilized-[Pd_3Cl_2] cluster filtered on polymer resin during catalysis, Pd K-edge EXAFS spectroscopy fitted data.

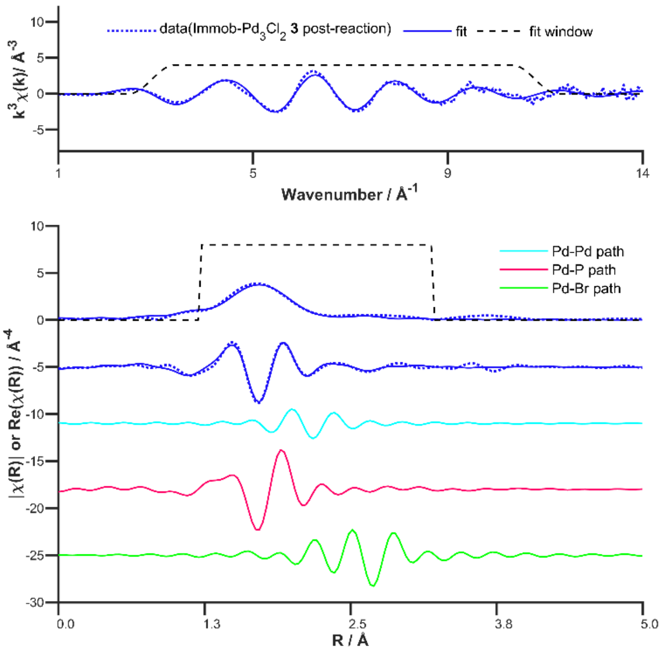

Figure S33 Immobilized-[Pd_3Cl_2] cluster filtered on polymer resin post-catalysis, Pd K-edge EXAFS spectroscopy fitted data.

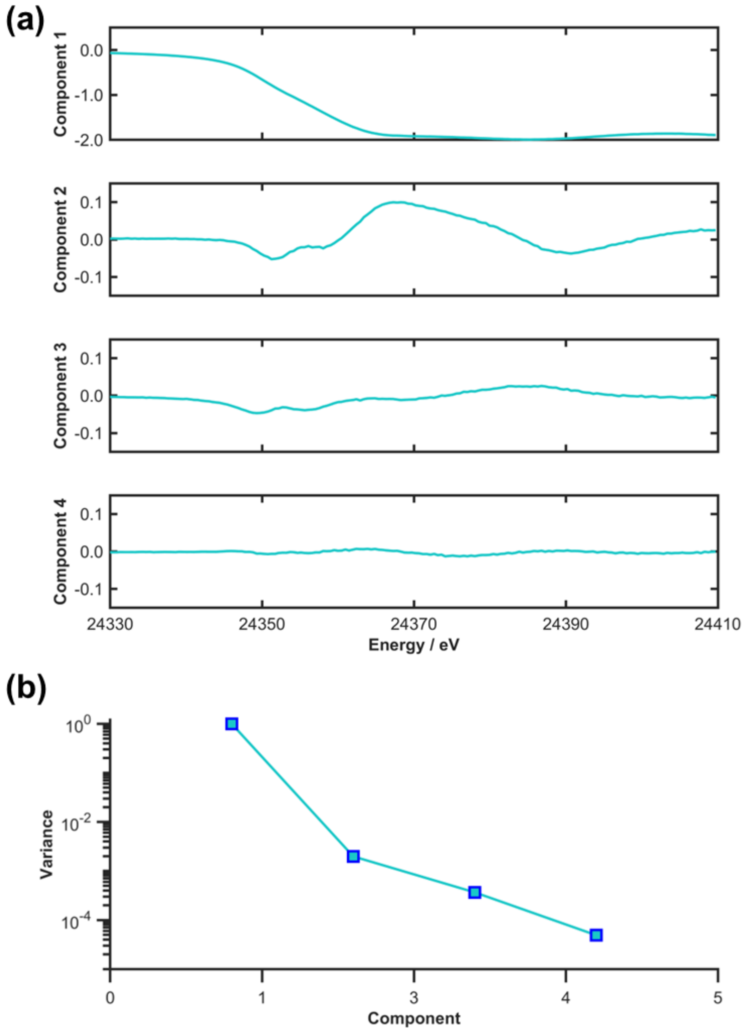

Figure S34 (**a**) PCA component plots derived from analysis of XANES region spectra from the series of 4 cryo-quenched solution samples with stepwise reagent addition reported in main text **Figure 9**. Note the y-scale of component 1 is different from the other three components. (**b**) Corresponding scree plot showing relative contribution of the components.

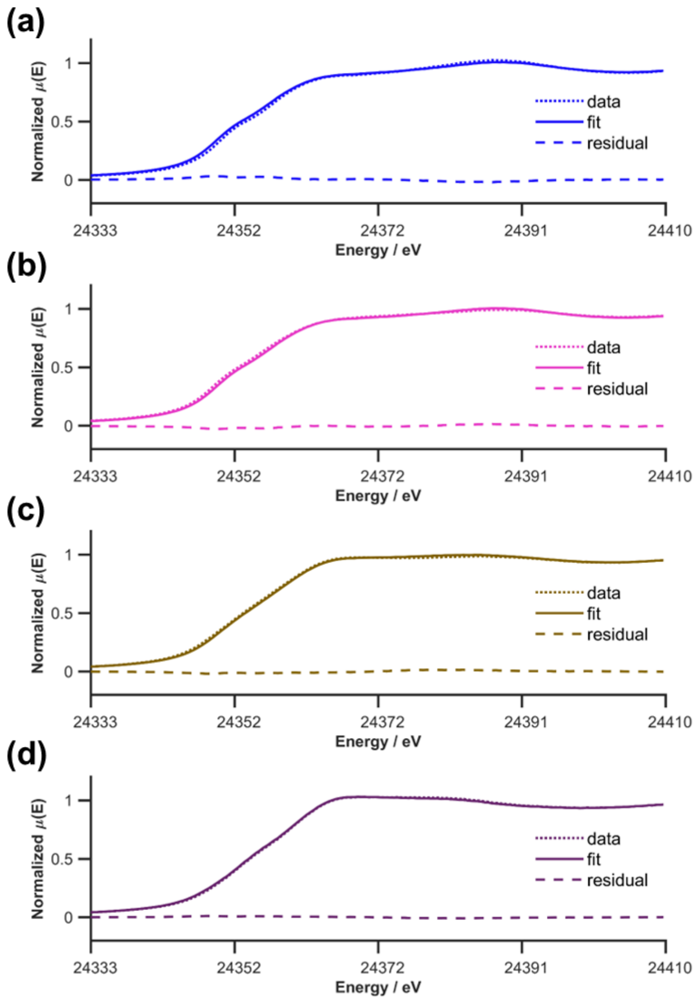

Figure S35 Reconstructions of data on which PCA analysis of XANES region in **Figure S34** was based using the first two components only: Pd_3Cl_2 cluster **1** alone (blue, **a**); after addition of H_2O/THF and *n*Bu_4NOH base (pink, **b**); after subsequent addition of phenylboronic acid (brown, **c**); and after subsequent addition of bromobenzene (platinate purple, **d**).

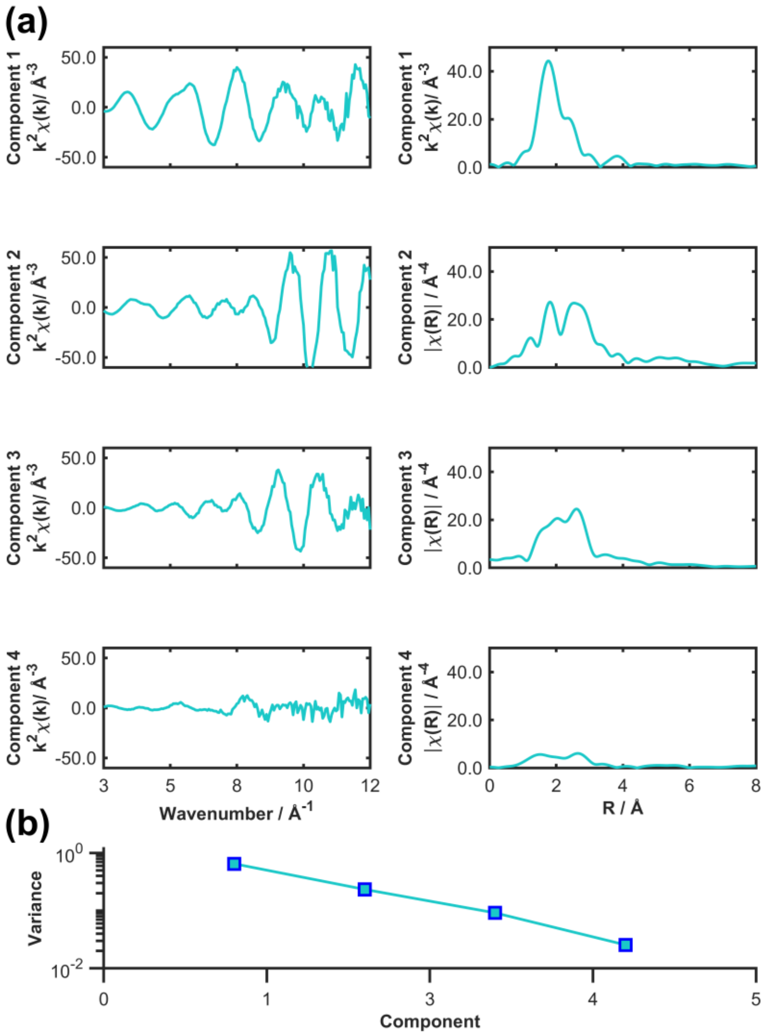

Figure S36 (**a**) PCA component plots derived from analysis of *k*-space EXAFS spectra from the series of 4 cryo-quenched solution samples with stepwise reagent addition reported in main text **Figure 9**, and corresponding Fourier transforms of data in the range $k = 3 - 12$. (**b**) Corresponding scree plot showing relative contribution of the components.

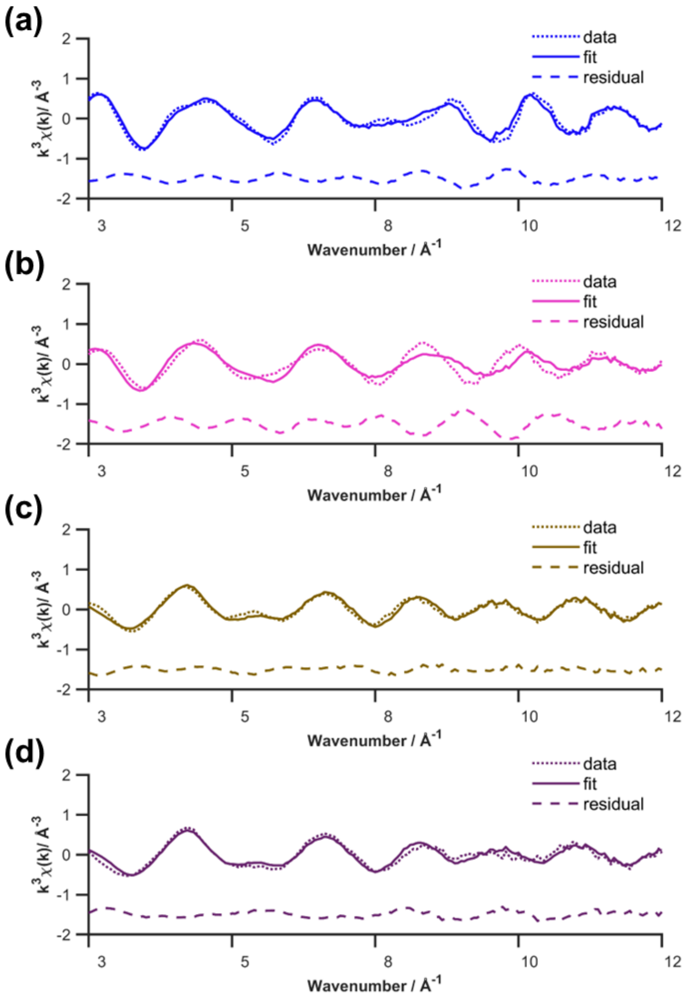

Figure S37 Reconstructions of data on which PCA analysis of *k*-space EXAFS in **Figure S36** was based using the first two components only: Pd_3Cl_2 cluster **1** alone (blue, **a**); after addition of H_2O/THF and *n*Bu_4NOH base (pink, **b**); after subsequent addition of phenylboronic acid (brown, **c**); and after subsequent addition of bromobenzene (platinate purple, **d**).

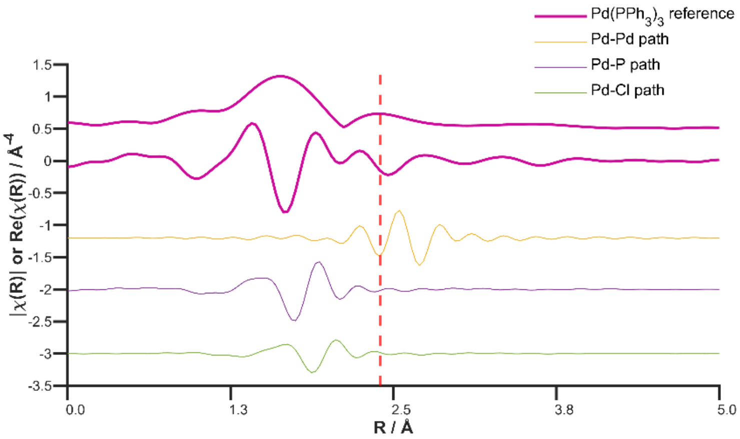

Figure S38 Pd K-edge *R*-space EXAFS data of $Pd(PPh_3)_3$ reference compound in THF, with the typical position used in the cluster fits of the contributing paths from Pd-Pd, Pd-P and Pd-Cl shown below. The red vertical line is a guide to the eye for the location of spectral weight in the data which is not accountable for in the absence of Pd-Pd co-ordination.

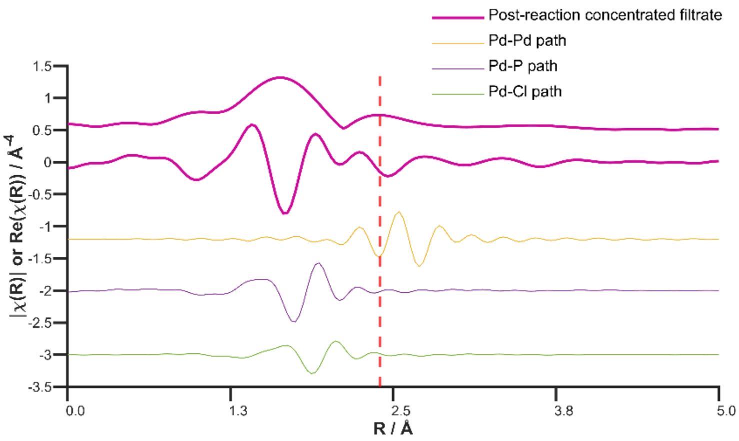

Figure S39 Pd K-edge *R*-space EXAFS data of the concentrated filtrate post-reaction using immob-Pd_3Cl_2 **3**, with the typical position used in the cluster fits of the contributing paths from Pd-Pd, Pd-P and Pd-Cl shown below. The red vertical line is a guide to the eye for the location of spectral weight in the data which is not accountable for in the absence of Pd-Pd co-ordination.

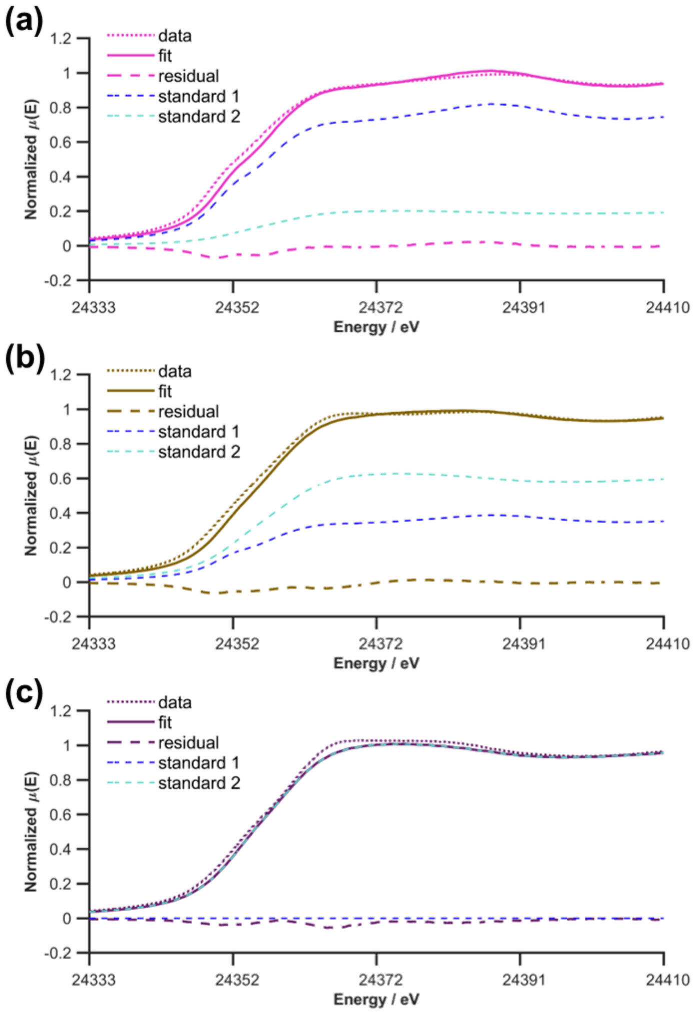

Figure S40 Linear combination fitting of data in XANES region using the initial Pd_3Cl_2 cluster **1** alone spectrum as standard 1 and the spectrum from the post-reaction concentrated filtrate material for the Immob-Pd_3Cl_2 reaction as standard 2, for the Pd_3Cl_2 cluster **1** after addition of H_2O/THF and $n$$Bu_4NOH$ base (pink, **a**); after subsequent addition of phenylboronic acid (brown, **b**); and after subsequent addition of bromobenzene (platinate purple, **c**).

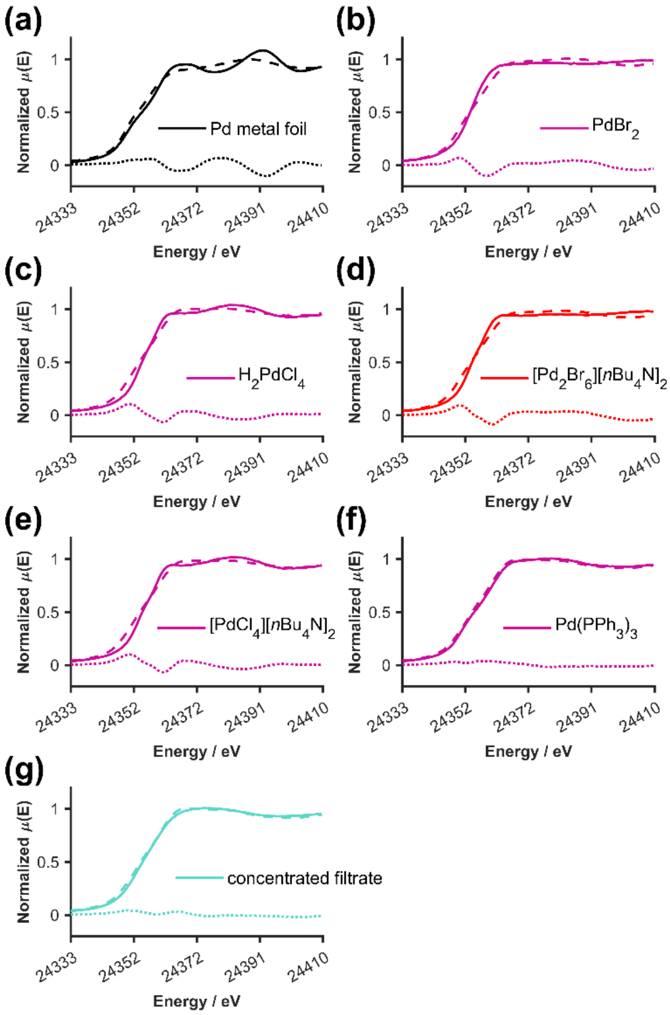

Figure S41 Target transform plots using first PCA two components for potentially contributing or similar reference species' spectra following PCA of XANES region spectra from the series of 4 cryo-quenched solution samples with stepwise reagent addition reported in main text **Figure 9**: Pd metal foil (**a**); $PdBr_2$ solid (**b**); H_2PdCl_4 in THF (**c**); $[Pd_2Br_6][nBu_4N]_2$ **11** in THF (**d**); $[PdCl_4][nBu_4N]_2$ in THF (**e**); $Pd(PPh_3)_3$ in THF (**f**); concentrated post-reaction filtrate solid material from reaction with immobPd$_3$Cl$_2$ **3** (**g**). Data for reference species shown in solid line, target transform with dashed line and residual with dotted line.

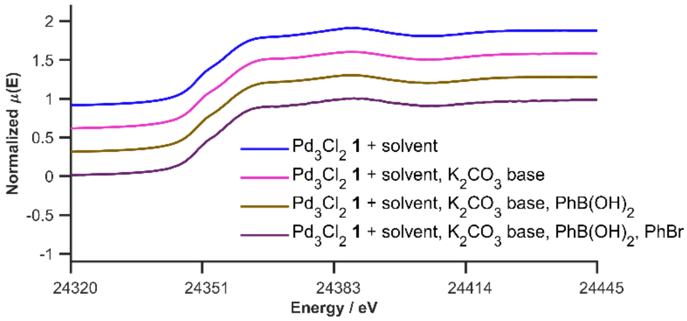

Figure S42 Pd K-edge XANES spectra of the Pd_3Cl_2 cluster **1** in 1:1 v/v 2-propanol:water solvent, and after stepwise addition and stirring with K_2CO_3 base (pink), phenylboronic acid (brown), and bromobenzene (platinate purple).

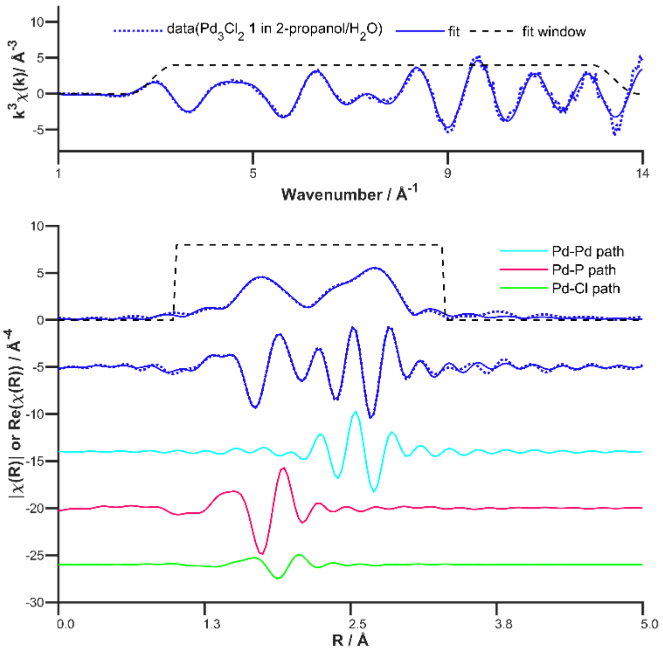

Figure S43 Pd K-edge EXAFS spectroscopy fitted data for the Pd_3Cl_2 cluster **1** in 1:1 v/v 2-propanol:water, showing a fit to the Pd3 cluster model as per 8.

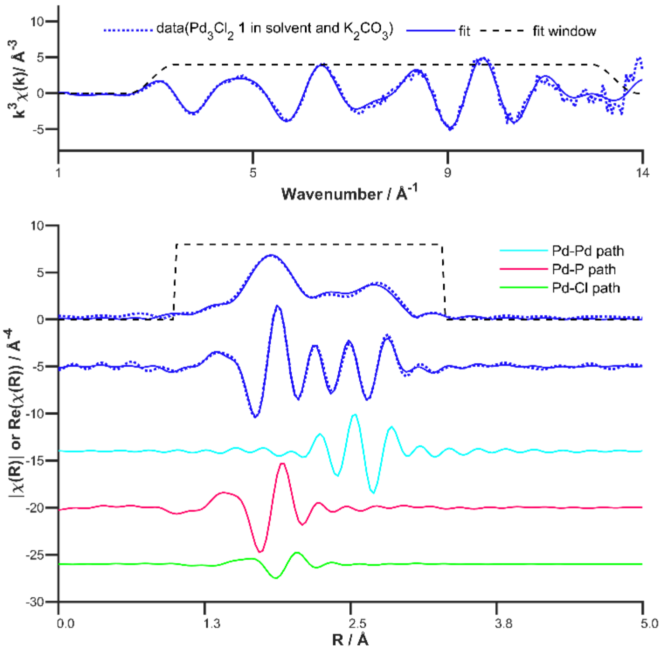

Figure S44 Pd K-edge EXAFS spectroscopy fitted data for the Pd_3Cl_2 cluster **1** in 1:1 v/v 2-propanol:water and subsequent addition and stirring with K_2CO_3 base, showing a fit to the Pd_3 cluster model as per 8.

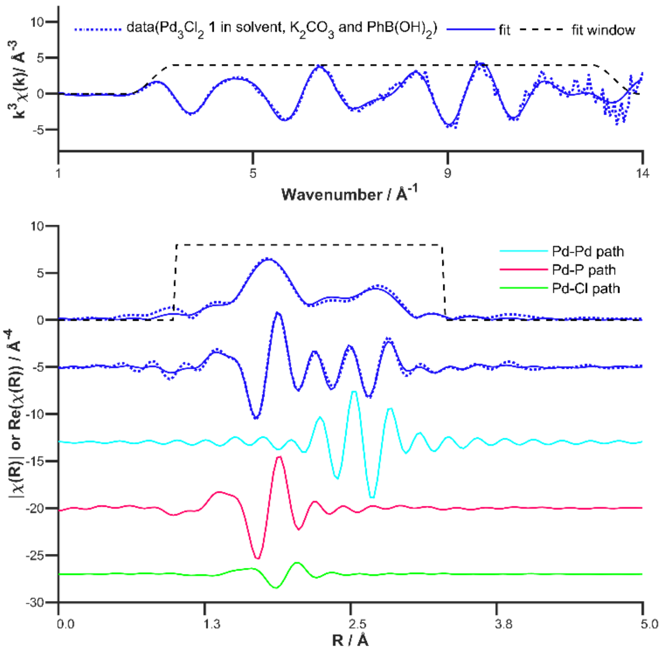

Figure S45 Pd K-edge EXAFS spectroscopy fitted data for the Pd_3Cl_2 cluster **1** in 1:1 v/v 2-propanol:water and subsequent stepwise addition and stirring with K_2CO_3 base and then phenylboronic acid, showing a fit to the Pd_3 cluster model as per Table S8.

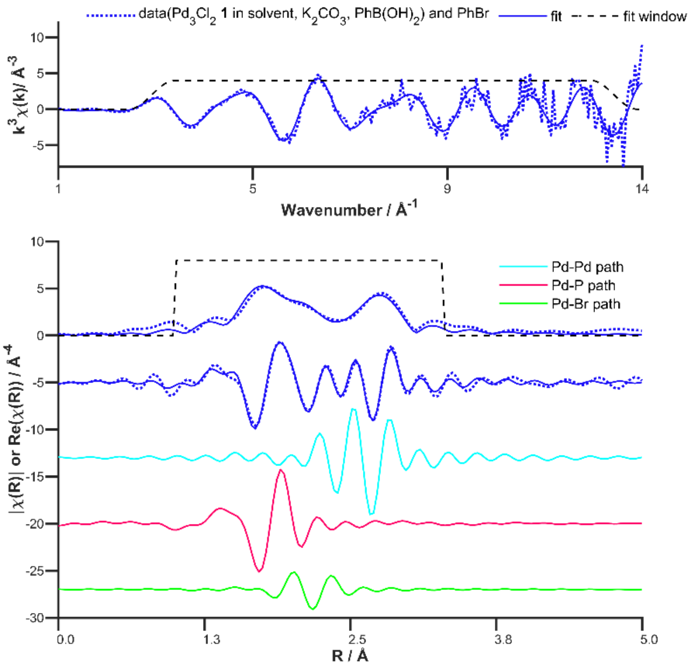

Figure S46 Pd K-edge EXAFS spectroscopy fitted data for the Pd_3Cl_2 cluster **1** in 1:1 v/v 2-propanol:water and subsequent stepwise addition and stirring with K_2CO_3 base, phenylboronic acid and then bromobenzene, showing a fit to the Pd_3 cluster model as per Table S8.

Table S6 EXAFS fitting parameters for the Pd K-Edge data of Immob-Pd_3Cl_2 **3** before reaction, during reaction and post-reaction, fitted as shown in the above figures/described in the experimental section to the Pd_3Cl_2 clusters using Pd, P (assumed equivalent) and halogen nearest neighbours.[a]

refined parameter	*Immob-[Pd_3Cl_2]*	*Immobilized catalyst filtered during reaction*	*Immobilized catalyst filtered post-reaction*
ΔE_0	4 (2)	0(2)	-9(4)
S_0^2	0.8	0.8	0.8
$CN_{Pd\text{-}Pd}$	2	2	2
$CN_{Pd\text{-}P}$	2.33	2.33	2.33
$CN_{Pd\text{-}X\ (X = Cl,\ Br)}$	0.667 (X = Cl)	0.667 (X = Br)	0.667 (X = Br)
$R_{Pd\text{-}Pd}$	2.97(3)	2.87(4)	2.8(1)
$R_{Pd\text{-}P}$	2.32(2)	2.26(2)	2.24(3)
$R_{Pd\text{-}X}$	2.56 (4) [b]	2.45(4)	2.45(5)
σ^2_{Pd}	0.015(4)	0.020(5)	0.028(14)
σ^2_{P}	0.009(2)	0.0077(8)	0.007(2)
σ^2_{X}	0.008(5)	0.012(5)	0.010(7)
Goodness of fit			
R-factor	0.020	0.006	0.018
Reduced χ^2	55.3	45.9	524
Fitting range			
k-range	3.0-11.4	3.0-11.2	3.0-11.2
R-range	1.0-3.0	1.2-3.2	1.2-3.2
No. of independent points	10.4	10.2	10.2
No. of fitted parameters	7	7	7

[a]Fitting parameters: $S_0^2 = 0.8$ as determined using a Pd foil standard. Parameters shown in blue were defined based on the structure rather than fitted variables. [b]This value appears slightly higher than expected, the reasons for which remain unclear, but a materially much worse fit is obtained with either aryl carbon or bromine in the bridging position.

Table S7 EXAFS fitting parameters for the Pd K-edge data of the untethered [Pd_3Cl_2] cluster **1** alone in dry dichloromethane under LN_2 quenched conditions (the first sample in the stepped reaction series in main text **Figure 9**).[a]

refined parameter	***[Pd_3Cl_2] 1***
ΔE_0	-2.6 (8)
S_0^2	0.85
$CN_{Pd\text{-}Pd}$	2
$CN_{Pd\text{-}P}$	2.33
$CN_{Pd\text{-}X\ (X = Cl,\ Br)}$	0.667 (X = Cl)
$R_{Pd\text{-}Pd}$	2.899(5)
$R_{Pd\text{-}P}$	2.261(9)
$R_{Pd\text{-}X}$	2.37(3)
σ^2_{Pd}	0.0034(4)
σ^2_{P}	0.004(2)
σ^2_{X}	0.005(5)
Goodness of fit	
R-factor	0.011
Reduced χ^2	35.2
Fitting range	
k-range	3.0-12.9
R-range	1.0-3.3
No. of independent points	14.2
No. of fitted parameters	7

[a]Fitting parameters: S_0^2 = 0.85 as determined using a Pd foil standard. Parameters shown in blue were defined based on the structure rather than fitted variables.

Table S8 EXAFS fitting parameters for the Pd K-edge data of the untethered Pd_3Cl_2 cluster **1** alone in 1:1 v/v 2-propanol:water solvent, and after stepwise addition and stirring with K_2CO_3 base, phenylboronic acid and bromobenzene analysed under LN_2 quenched conditions (the samples in the stepped reaction series in **Figure S42**).[a]

refined parameter	***[Pd₃Cl₂] 1*** *2-propanol water*	***[Pd₃Cl₂] 1*** *2-propanol water + K_2CO_3*	***[Pd₃Cl₂] 1*** *2-propanol water + K_2CO_3 + $PhB(OH)_2$*	***[Pd₃Cl₂] 1*** *2-propanol water + K_2CO_3 + $PhB(OH)_2$ + PhBr*
ΔE_0	4.0 (4)	5.7(6)	4.9(6)	4.5(7)
S_0^2	0.85	0.85	0.85	0.85
$CN_{Pd\text{-}Pd}$	2	2	2	2
$CN_{Pd\text{-}P}$	2.33	2.33	2.33	2.33
$CN_{Pd\text{-}X\ (X = Cl,\ Br)}$	0.667 (X = Cl)	0.667 (X = Cl)	0.667 (X = Cl)	0.667 (X = Br)
$R_{Pd\text{-}Pd}$	2.909(3)	2.889(6)	2.895(7)	2.918(8)
$R_{Pd\text{-}P}$	2.266(4)	2.268(4)	2.263(7)	2.272(7)
$R_{Pd\text{-}X}$	2.39(2)	2.40(4)	2.37(3)	2.500(9)
σ^2_{Pd}	0.0039(2)	0.0060(4)	0.0065(6)	0.0055(7)
σ^2_{P}	0.0048(5)	0.0034(5)	0.003(1)	0.0042(6)
σ^2_{X}	0.004(2)	0.02(1)	-0.01(2)	0.0021(8)
Goodness of fit				
R-factor	0.003	0.006	0.01	0.01
Reduced χ^2	16.0	29.5	35.9	11.0
Fitting range				
k-range	3.0-12.9	3.0-12.9	3.0-12.9	3.0-12.9
R-range	1.0-3.3	1.0-3.3	1.0-3.3	1.0-3.3
No. of independent points	14.2	14.2	14.2	14.2
No. of fitted parameters	7	7	7	7

[a]Fitting parameters: S_0^2 = 0.85 as determined using a Pd foil standard. Parameters shown in blue were defined based on the structure rather than fitted variables.

3 NMR Spectral Data

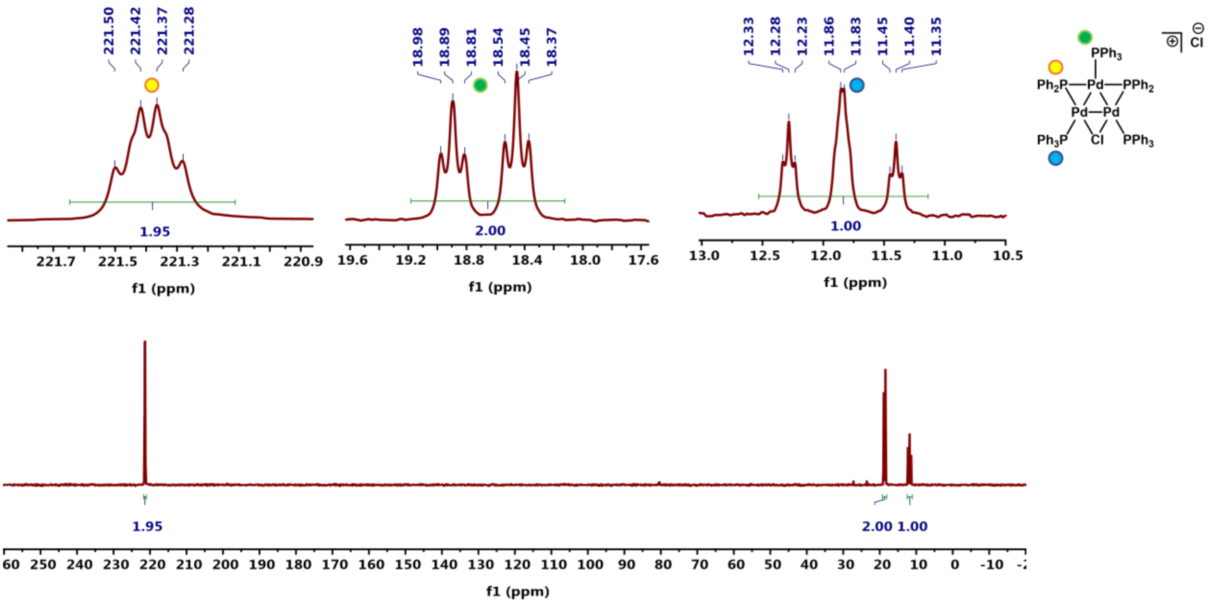

Figure S47 $^{31}P\{^{1}H\}$ NMR spectrum of Pd_3Cl_2 cluster $[Pd_3(\mu\text{-}Cl)(\mu\text{-}PPh_2)_2(PPh_3)_2][Cl]$ in CD_2Cl_2

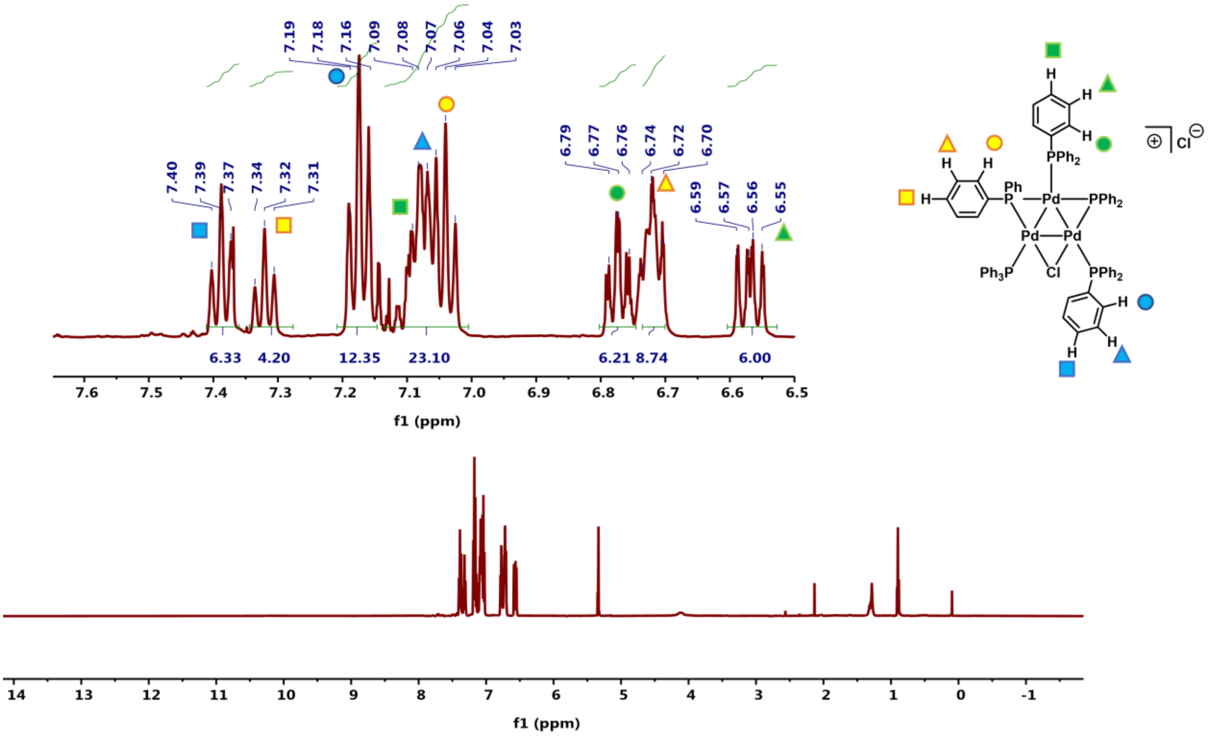

Figure S48 ^{1}H NMR spectrum of Pd_3Cl_2 cluster $[Pd_3(\mu\text{-}Cl)(\mu\text{-}PPh_2)_2(PPh_3)_2][Cl]$ in CD_2Cl_2

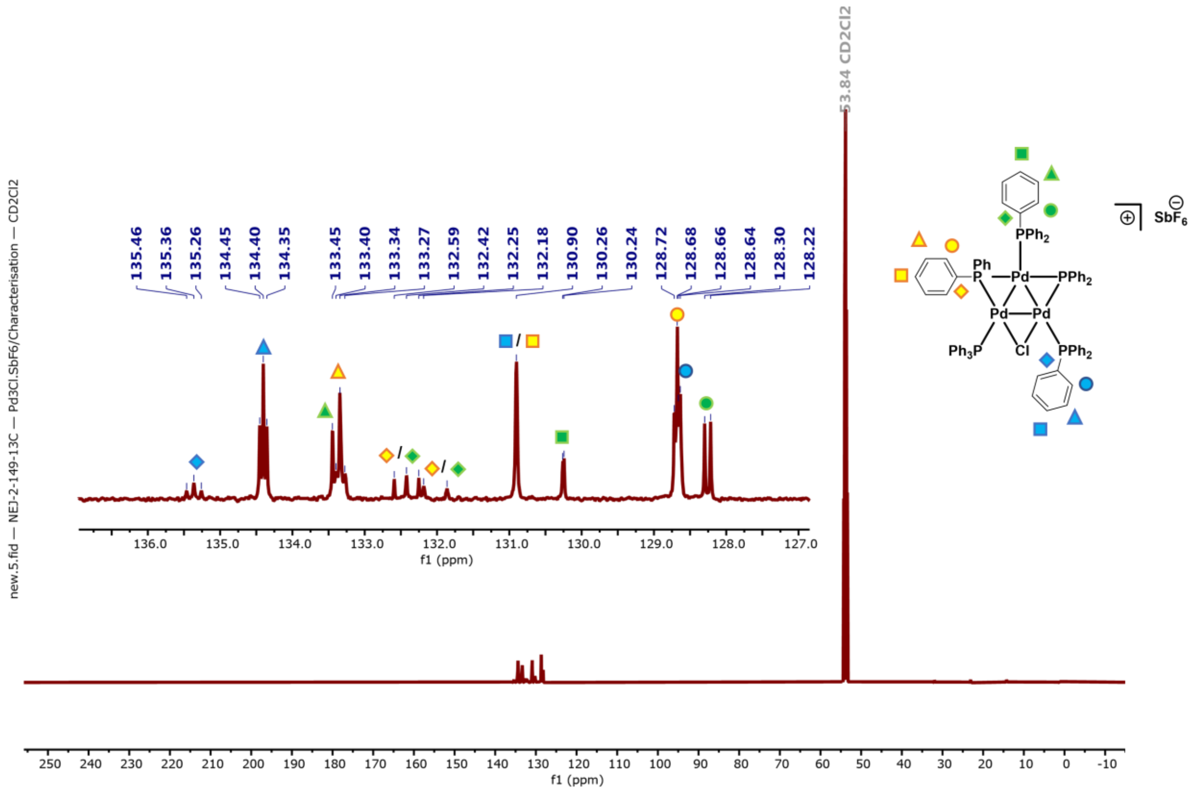

Figure S49 $^{13}C\{^{1}H\}$ NMR spectrum of Pd_3Cl_2 cluster $[Pd_3(\mu\text{-}Cl)(\mu\text{-}PPh_2)_2(PPh_3)_2][SbF_6]$ in CD_2Cl_2

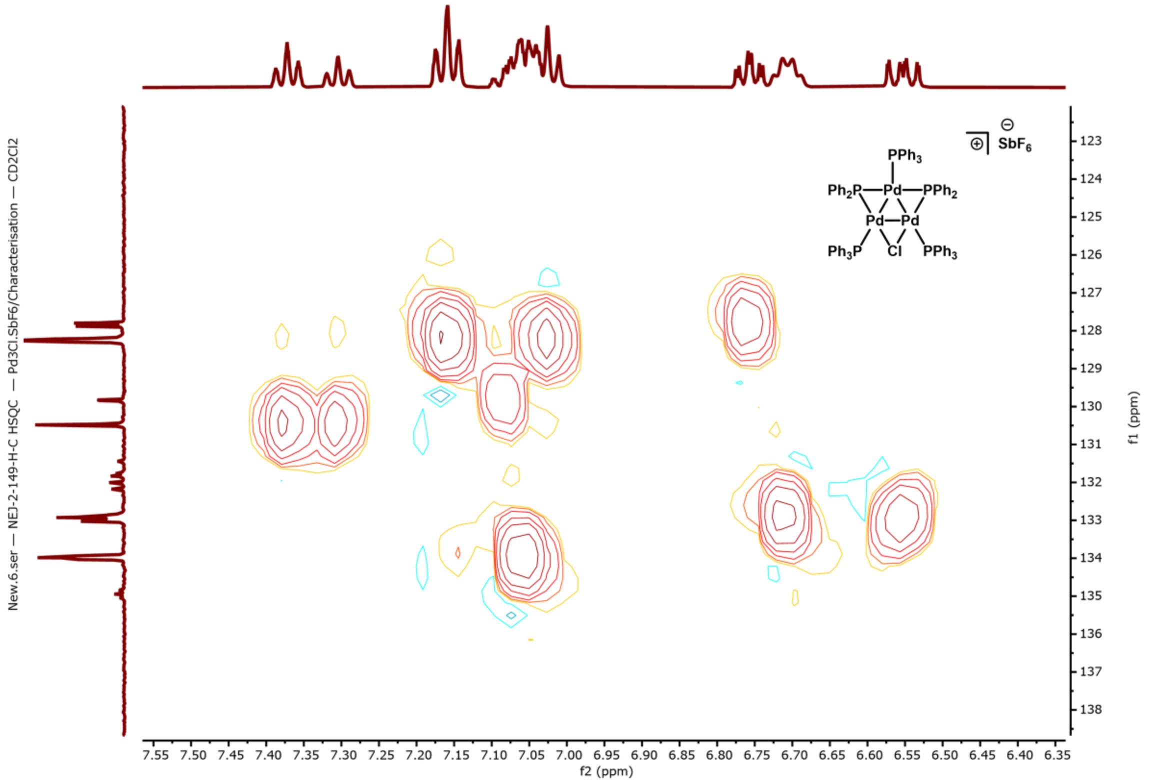

Figure S50 ^{1}H-^{13}C HSQC NMR spectrum of Pd_3Cl_2 cluster $[Pd_3(\mu\text{-}Cl)(\mu\text{-}PPh_2)_2(PPh_3)_2][SbF_6]$ in CD_2Cl_2

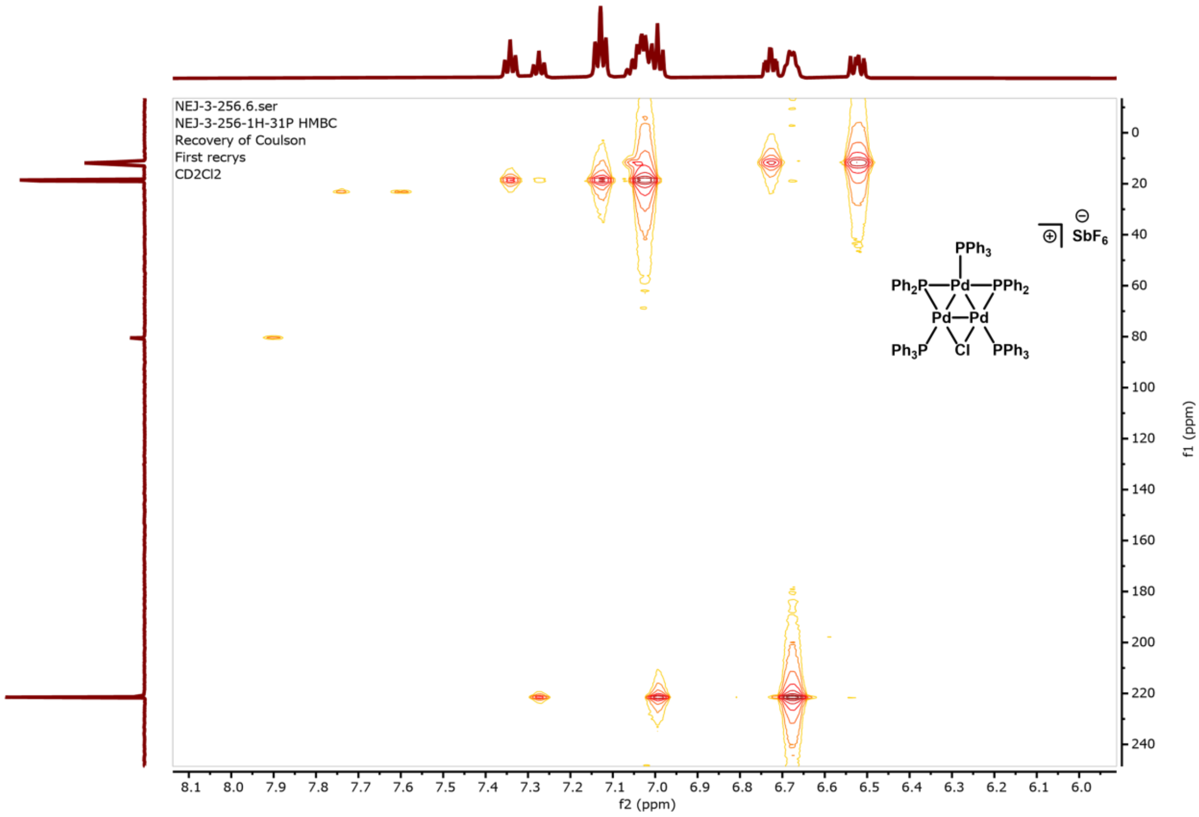

Figure S51 ^{1}H-^{31}P HMBC NMR spectrum of Pd_3Cl_2 cluster $[Pd_3(\mu\text{-}Cl)(\mu\text{-}PPh_2)_2(PPh_3)_2][SbF_6]$ in CD_2Cl_2

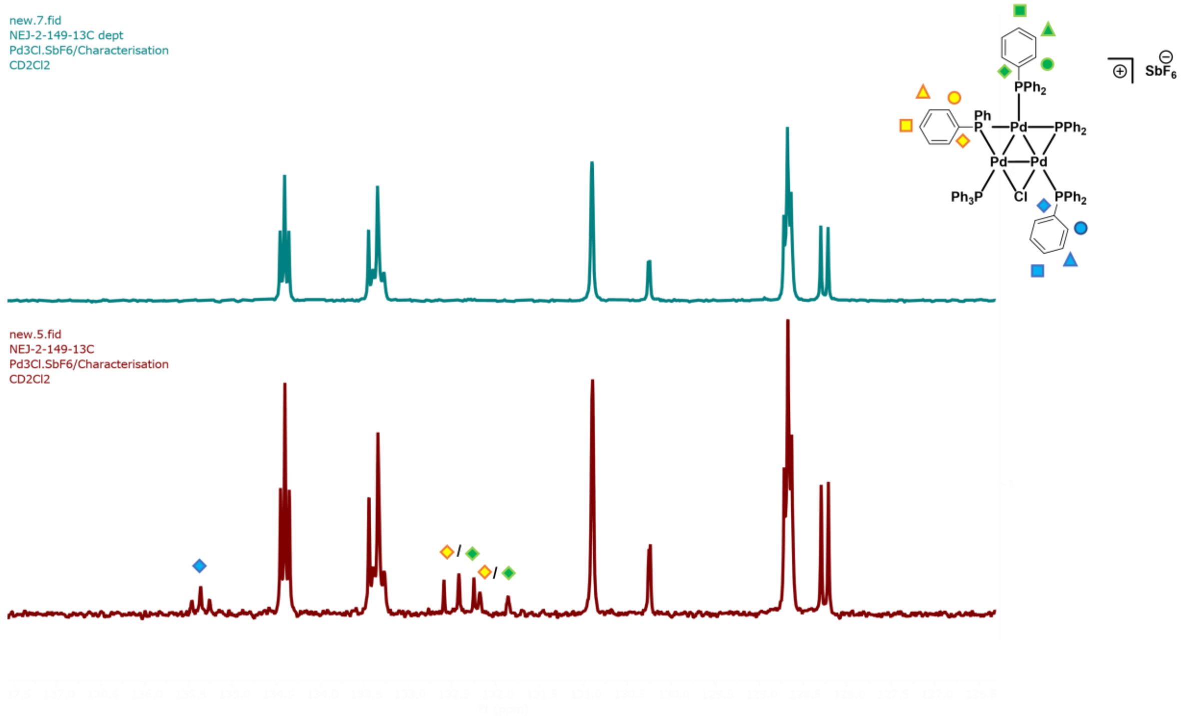

Figure S52 Stack spectra of $^{13}C\{^{1}H\}$ NMR of Pd_3Cl_2 cluster $[Pd_3(\mu\text{-}Cl)(\mu\text{-}PPh_2)_2(PPh_3)_2][SbF_6]$ (below) and ^{13}C dept-135 (top) in CD_2Cl_2

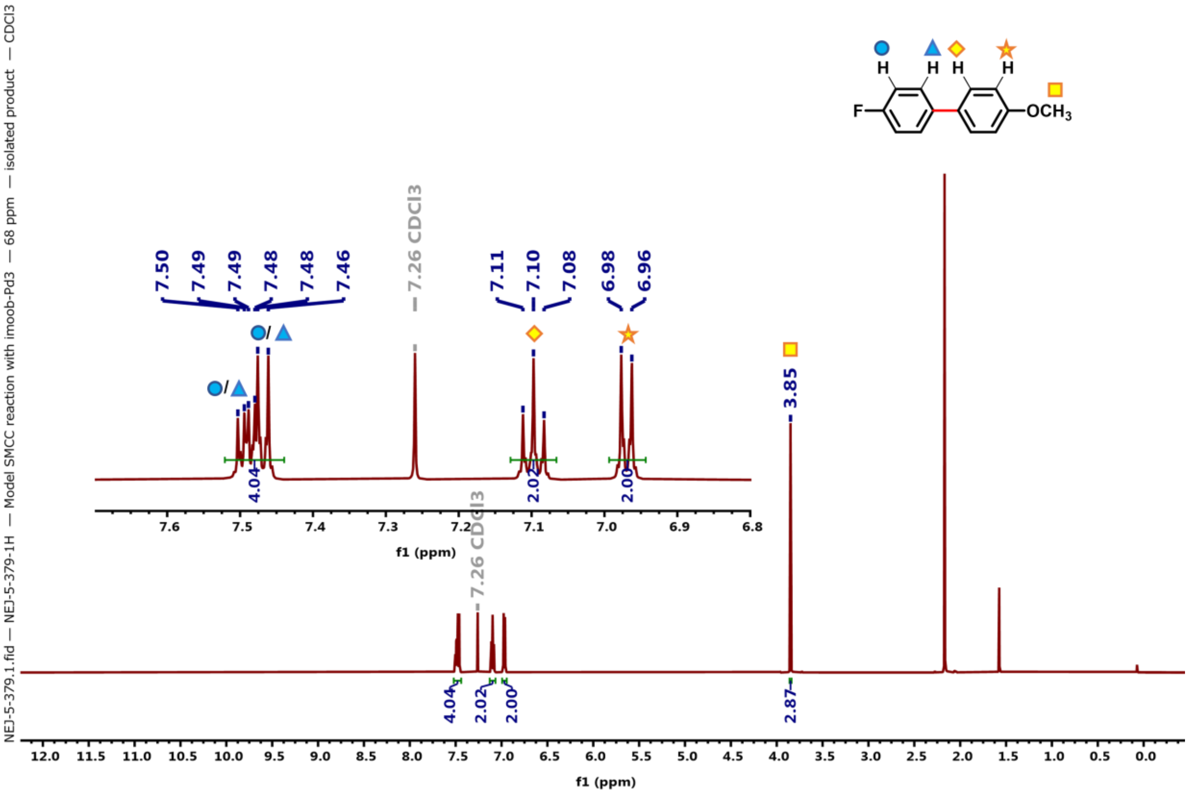

Figure S53 ^{1}H NMR spectrum of $C_{13}H_{11}FO$ in $CDCl_3$

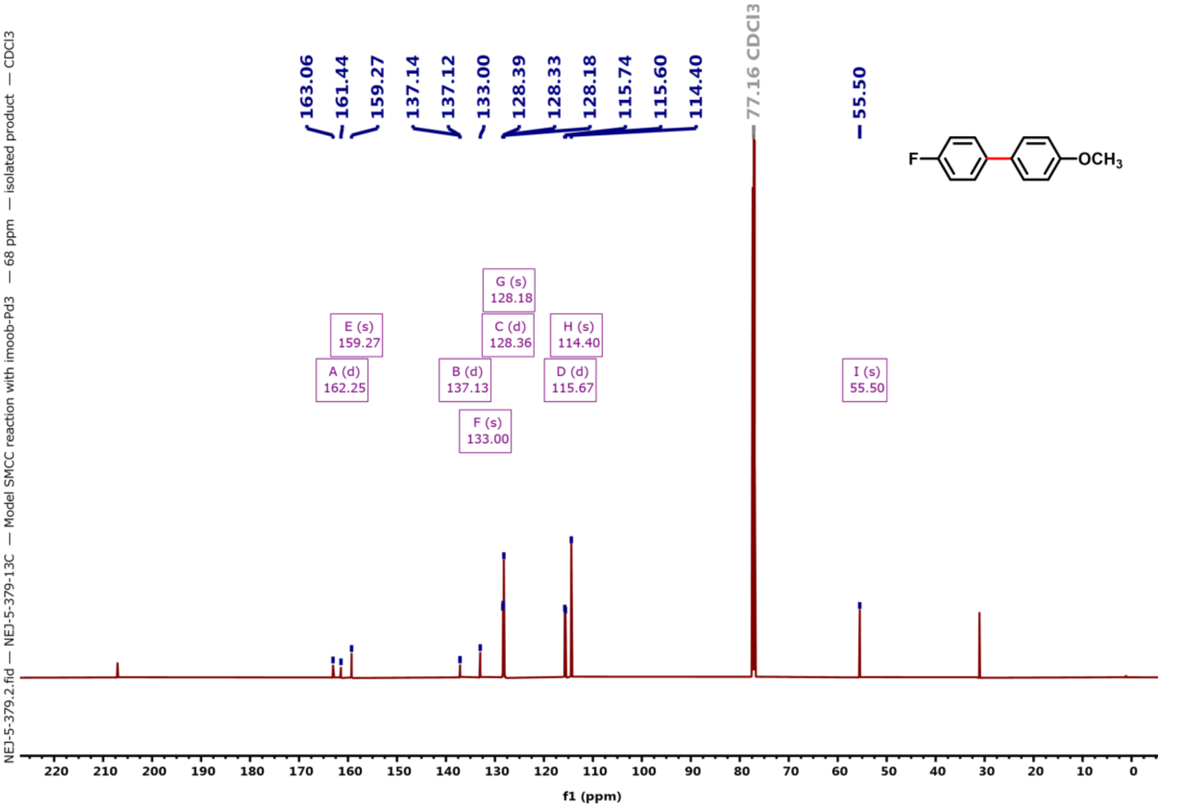

Figure S54 ^{13}C{^{1}H} NMR spectrum of $C_{13}H_{11}FO$ in $CDCl_3$, signals at δ 207 and 31 are for acetone impurity in the sample.

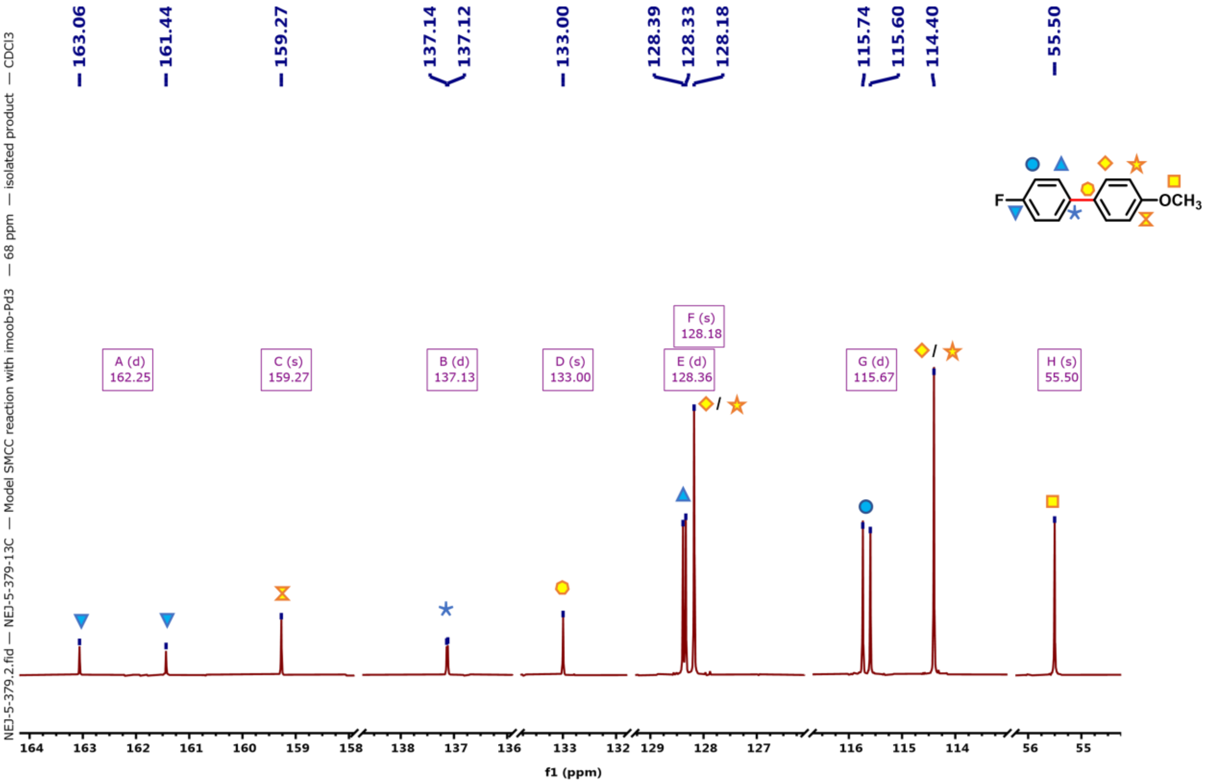

Figure S55 $^{13}C\{^{1}H\}$ NMR spectrum of $C_{13}H_{11}FO$ in $CDCl_3$ with assigned carbon peaks.

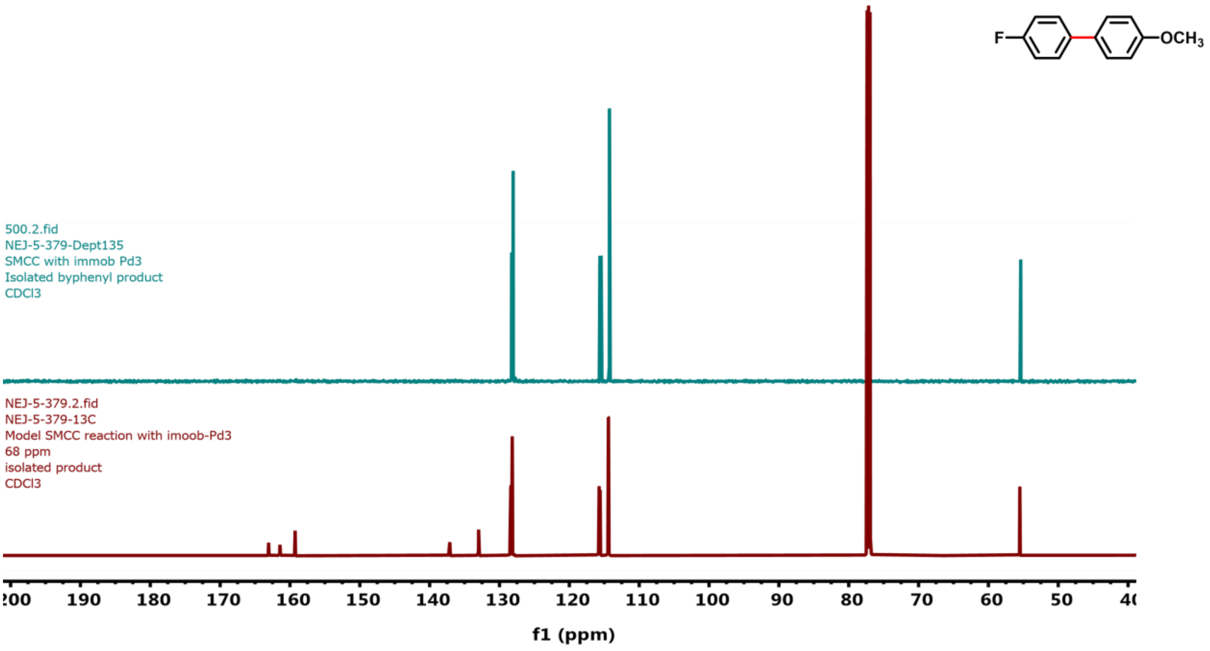

Figure S56 Stack spectra of $^{13}C\{^{1}H\}$ NMR of $C_{13}H_{11}FO$ (below) and ^{13}C dept-135 (top) in $CDCl_3$.

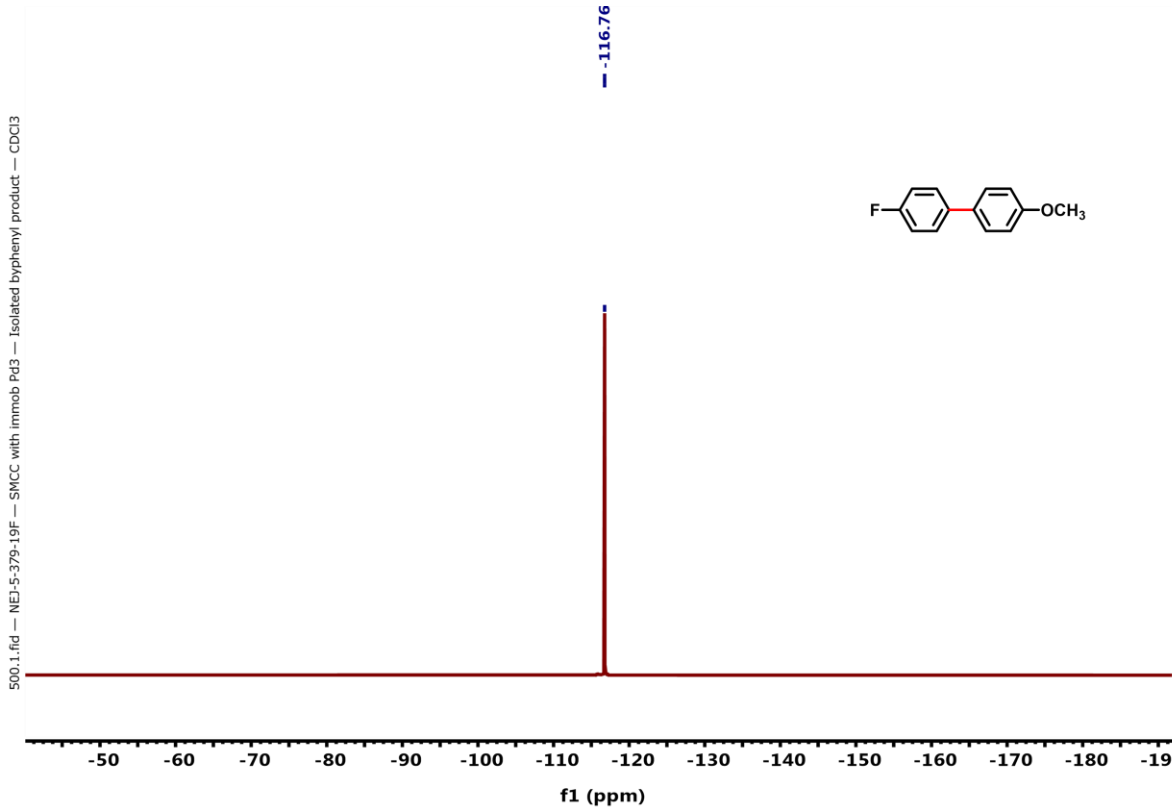

Figure S57 $^{19}F\{^1H\}$ NMR spectrum of $C_{13}H_{11}FO$ in $CDCl_3$.

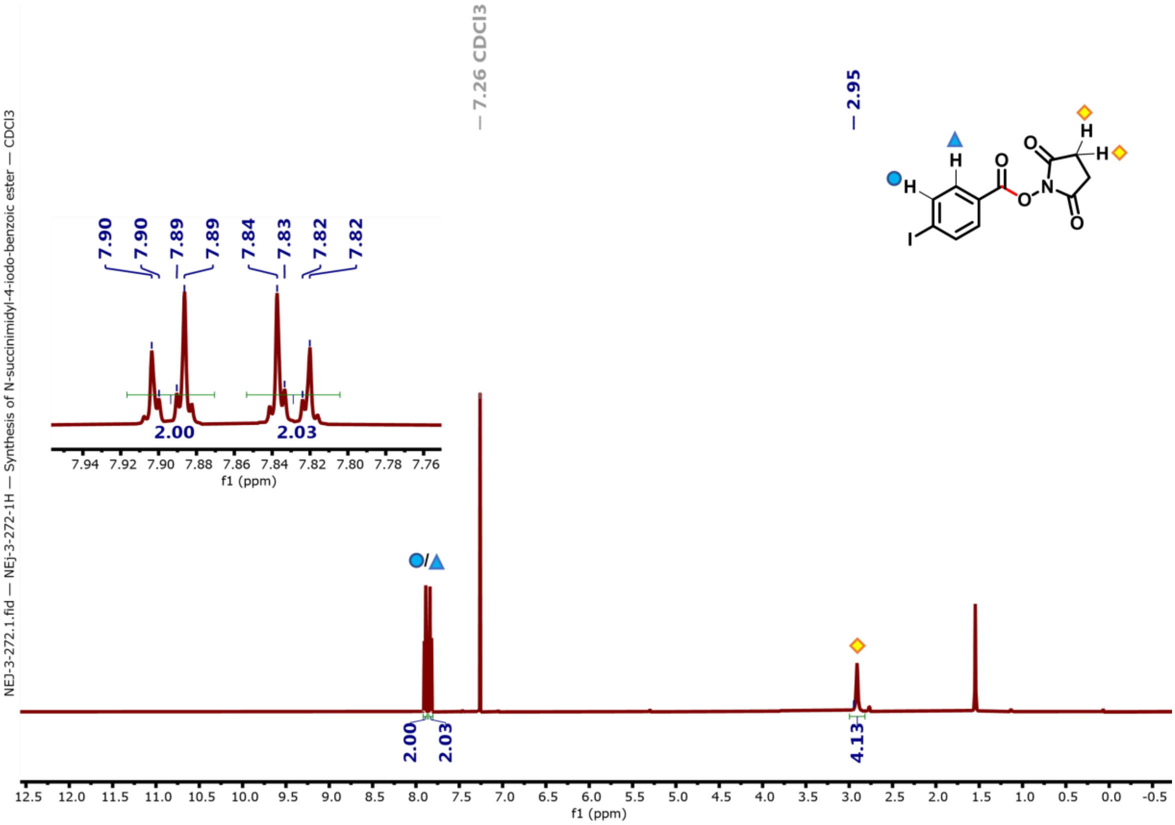

Figure S58 ^1H NMR spectrum of $C_{11}H_8INO_4$ in $CDCl_3$

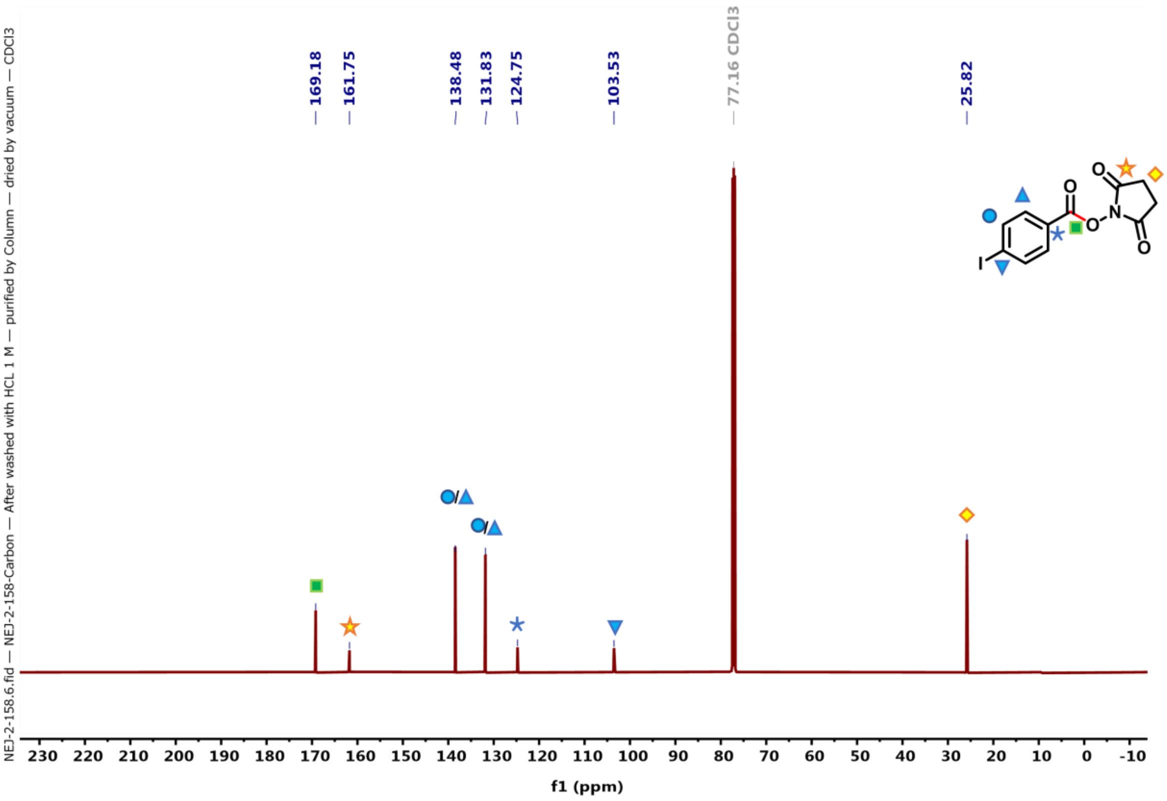

Figure S59 $^{13}C\{^1H\}$ NMR spectrum of $C_{11}H_8INO_4$ in $CDCl_3$ with assigned carbon peaks.

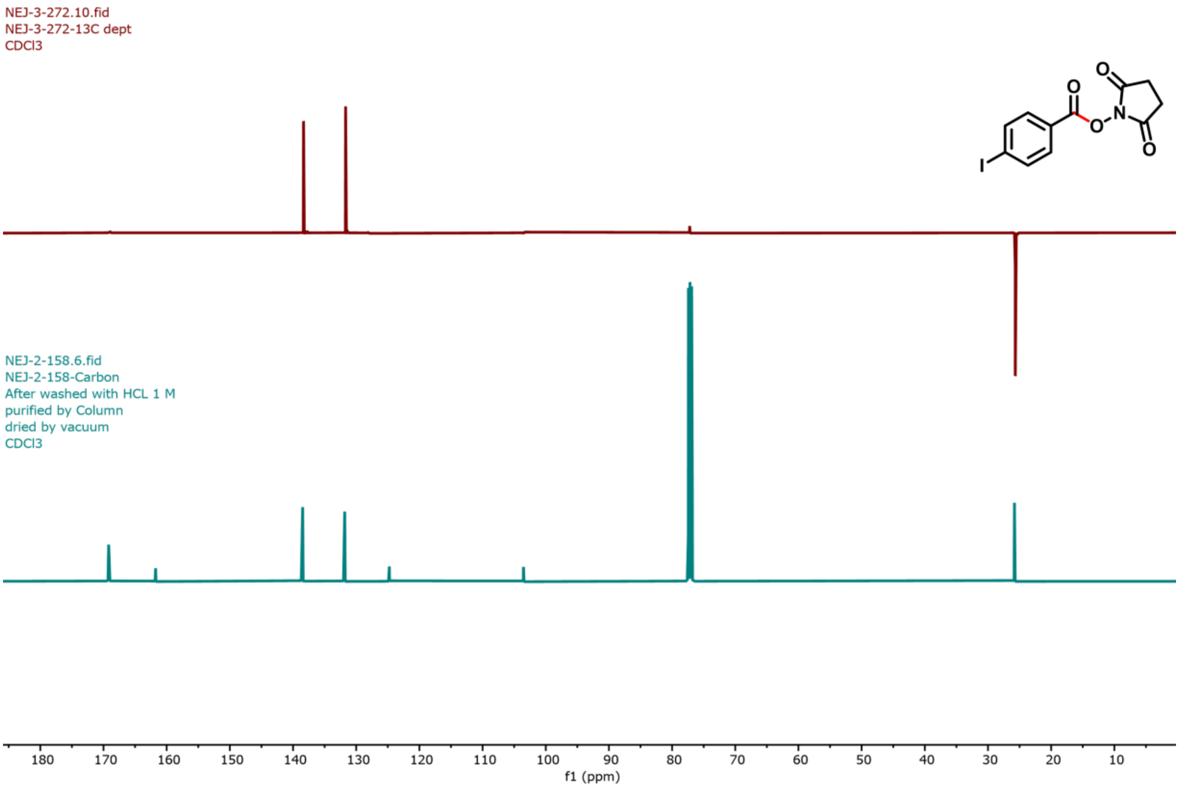

Figure S60 Stack spectra of $^{13}C\{^1H\}$ NMR of $C_{11}H_8INO_4$ (below) and ^{13}C dept-135 (top) in $CDCl_3$.

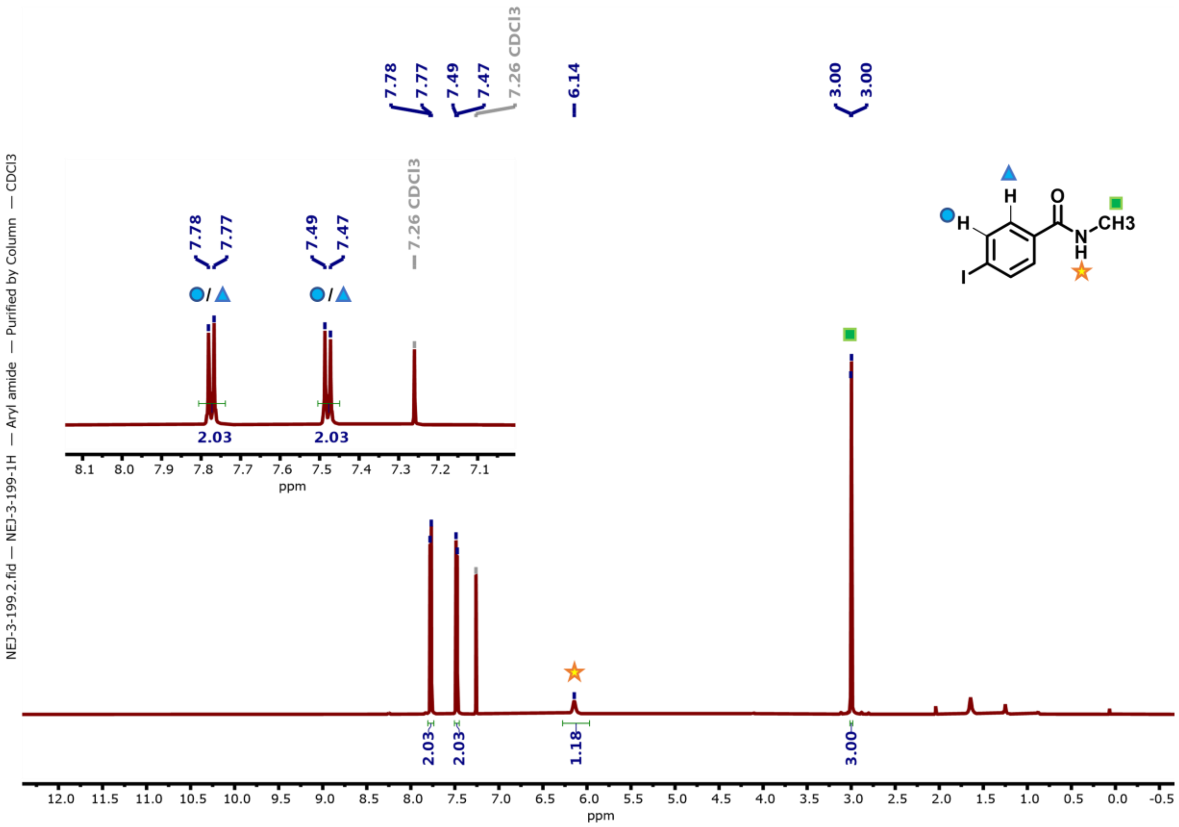

Figure S61 ^{1}H NMR spectrum of C_8H_8INO in $CDCl_3$

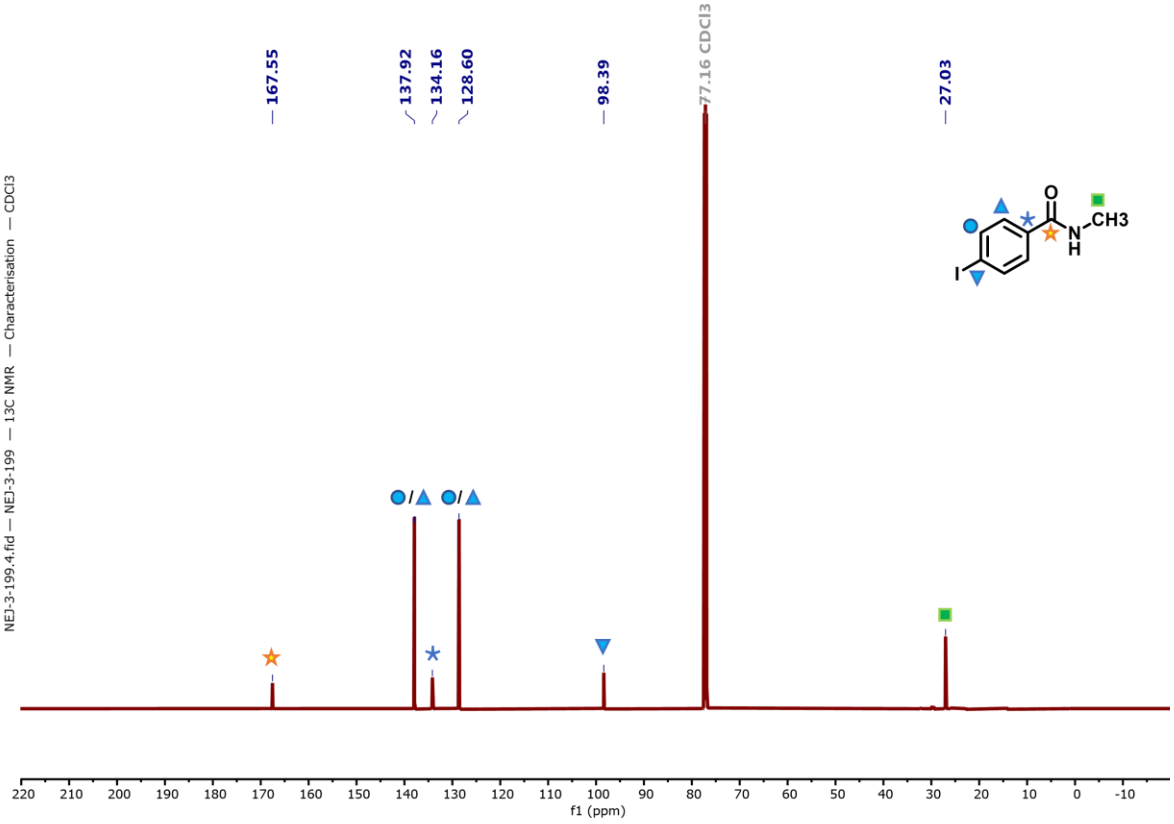

Figure S62 $^{13}C\{^1H\}$ NMR spectrum of C_8H_8INO in $CDCl_3$ with assigned carbon peaks.

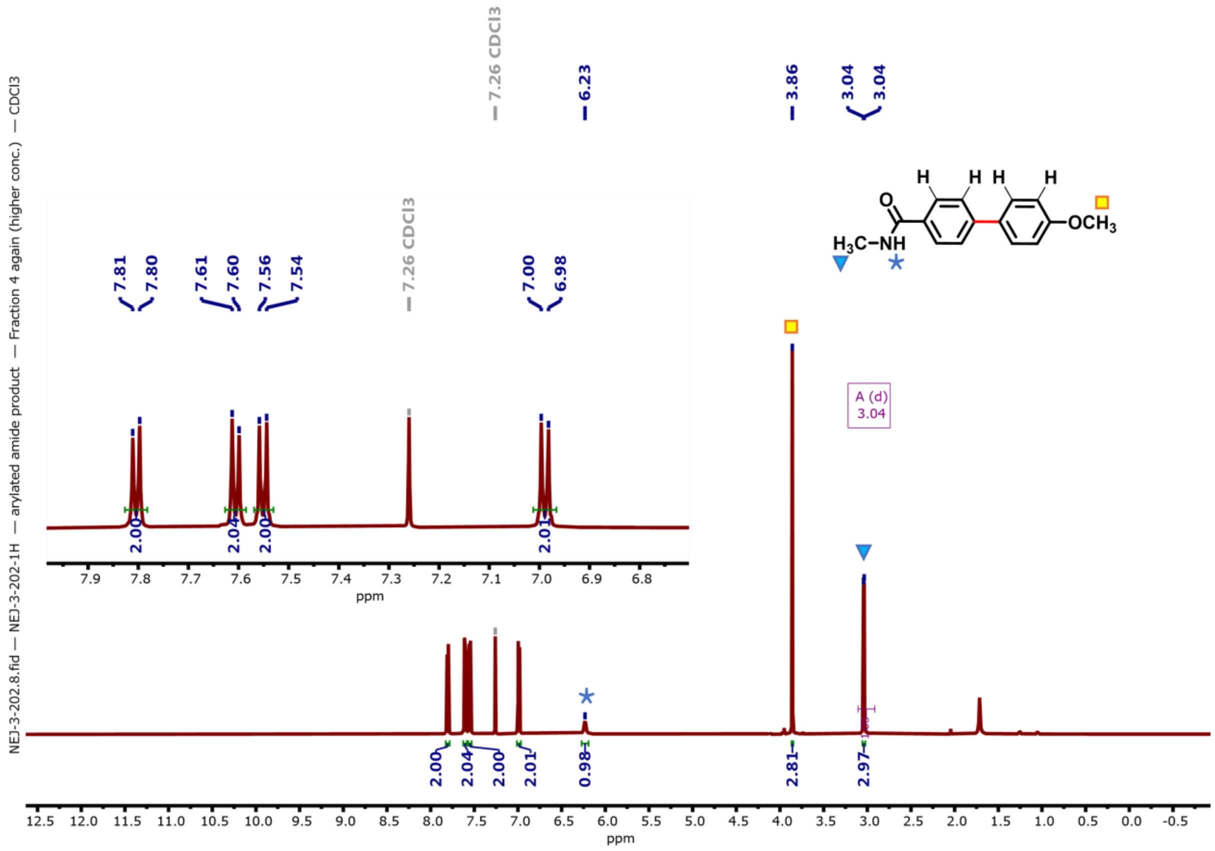

Figure S63 ^{1}H NMR spectrum of $C_{15}H_{15}NO_2$ in $CDCl_3$

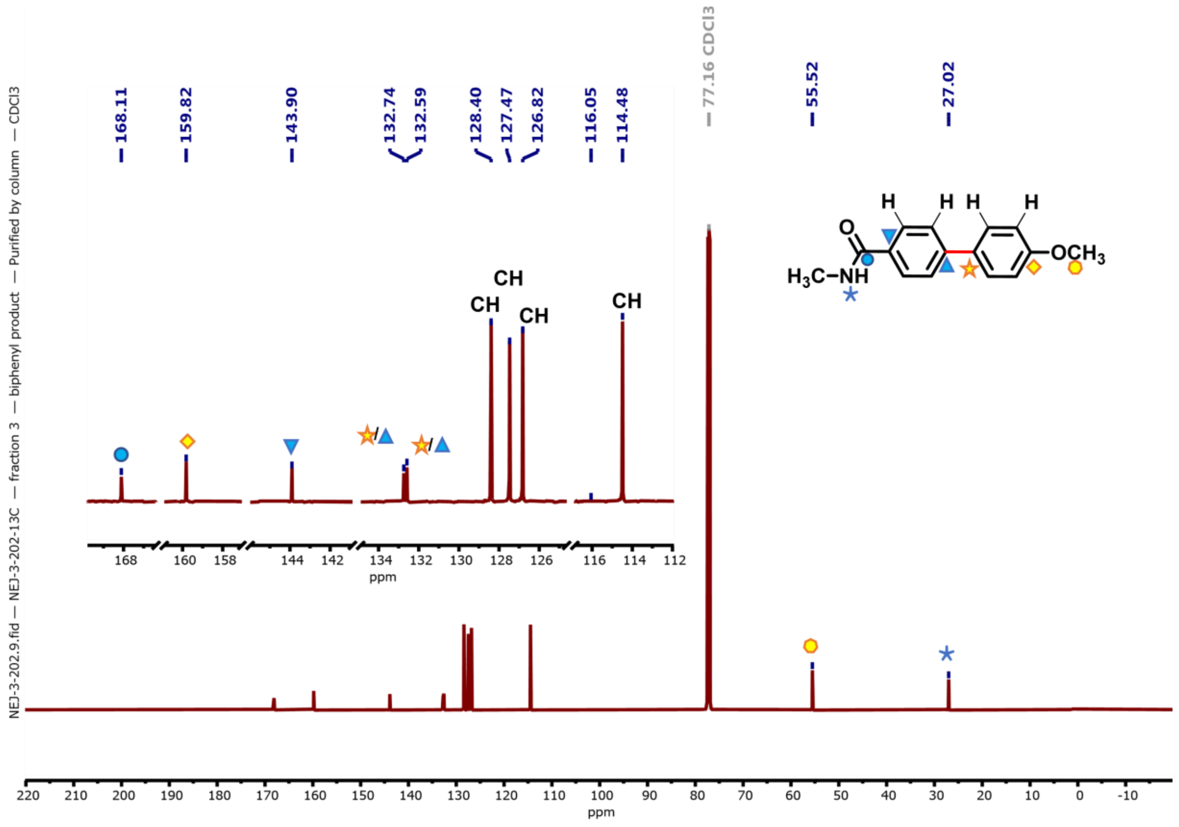

Figure S64 $^{13}C\{^{1}H\}$ NMR spectrum of $C_{15}H_{15}NO_2$ in $CDCl_3$ with assigned carbon peaks.

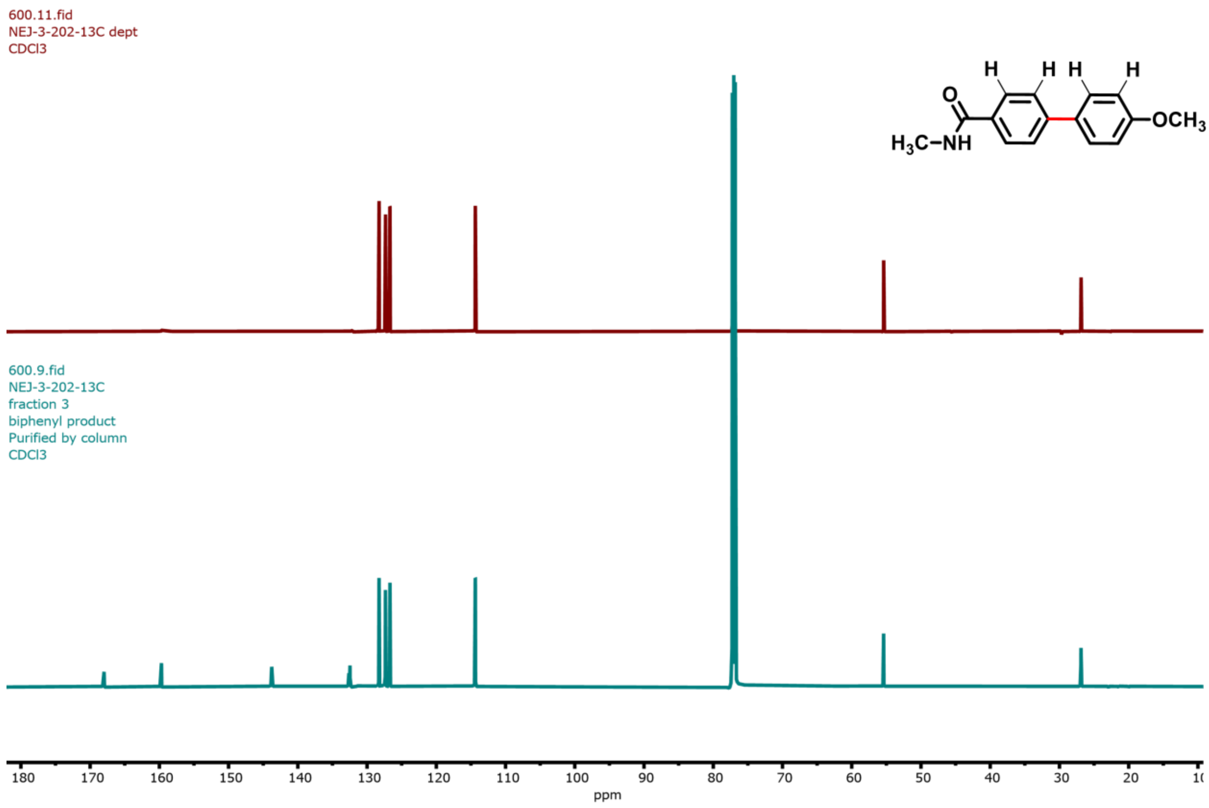

Figure S65 Stack spectra of $^{13}C\{^{1}H\}$ NMR of $C_{15}H_{15}NO_2$ (below) and ^{13}C dept-135 (top) in $CDCl_3$.

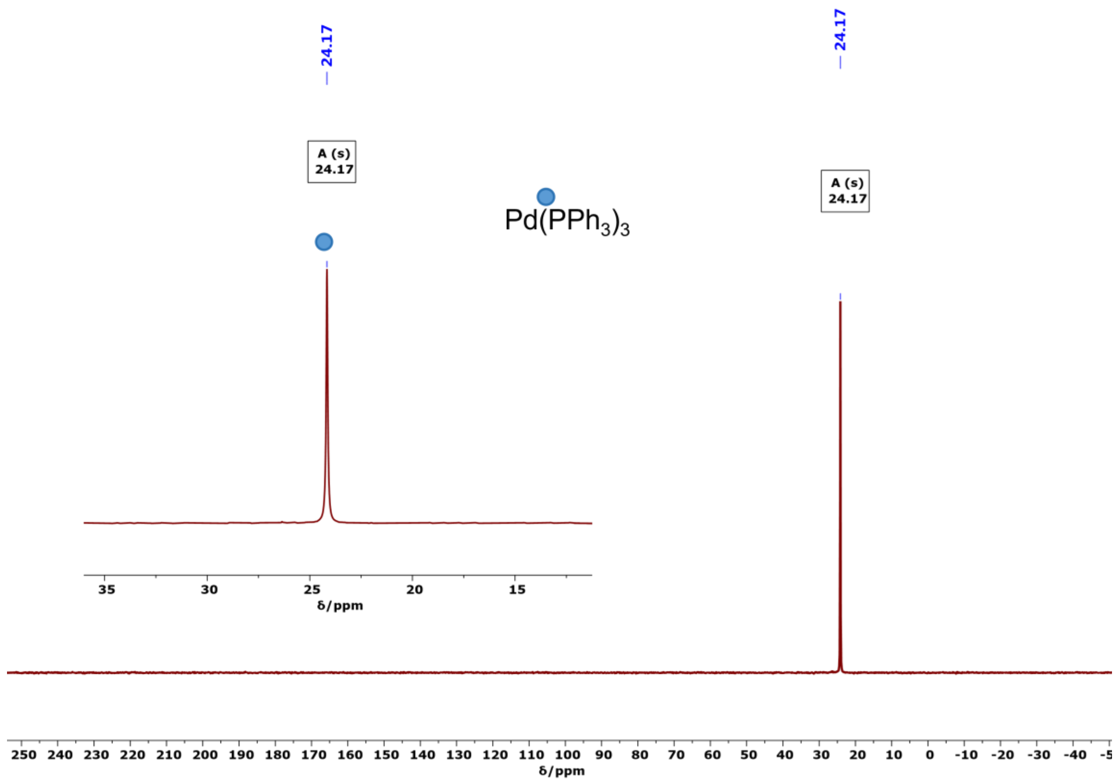

Figure S66 $^{31}P\{^{1}H\}$ NMR spectrum of $Pd(PPh_3)_3$ in benzene-d_6

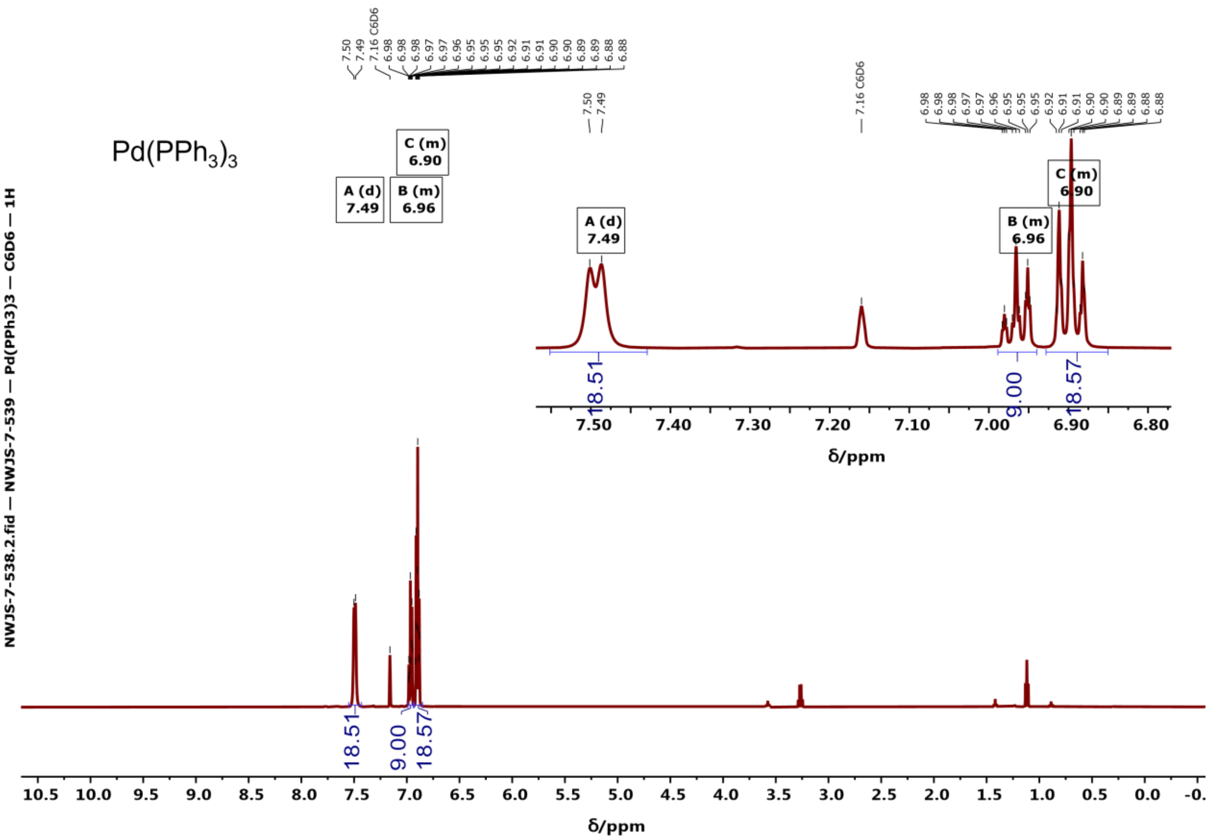

Figure S67 ^{1}H NMR spectrum of $Pd(PPh_3)_3$ in benzene-d_6

4 X-ray Crystallography

Crystal structure determination of [ijsf1923]

Experimental: Single crystals of $C_{78}H_{65}ClF_6P_5Pd_3Sb$ [ijsf1923] were grown from DCM and Hexane. A suitable crystal was selected and mounted on a SuperNova, Dual, Cu at home/near, Eos diffractometer (Oil on 100um micromount). The crystal was kept at 110.00(10) K during data collection. Using Olex2,[16] the structure was solved with the ShelXT[17] structure solution program using Intrinsic Phasing and refined with the ShelXL[18] refinement package using Least Squares minimization.

For synthesis procedure and characterization data, see section 2.1.

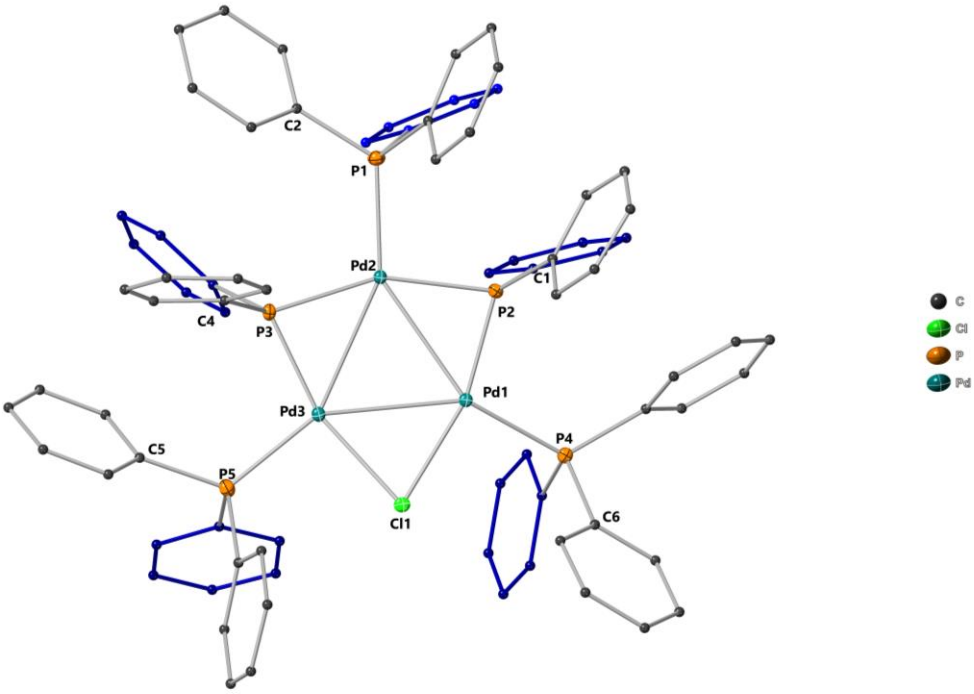

Figure S68 X-ray diffraction of single crystal structure of $[Pd_3(\mu\text{-}Cl)(\mu\text{-}PPh_2)_2(PPh_3)_3][SbF_6]$, thermal ellipsoids at 40% probability. Hydrogen atoms and counterion $[SbF_6]^-$ were removed for clarity.

Table S9 Principal bond length and bond angles for $[Pd_3(\mu\text{-}Cl)(\mu\text{-}PPh_2)_2(PPh_3)_3][SbF_6]$ (ijsf1923).

Selected bond	***Selected bond length (Å)***
Pd(1)–P(2)	2.2002(12)
Pd(1)–P(4)	2.2809(13)
Pd(1)–Pd(2)	2.9085(10)
Pd(1)–Pd(3)	2.8712(8)
Pd(1)–Cl(1)	2.3897(13)
Pd(3)–Cl(1)	2.3884(12)
Pd(2)–P(2)	2.2840(12)
Pd(2)–P(1)	2.3039(11)
Pd(2)–Pd(3)	2.9122(13)
P(1)–C(2)	1.8307(31)
P(2)–C(1)	1.8066(32)
Selected bond	***Selected bond angle (º)***
Pd(1)-Cl(1)-Pd(3)	73.870(2)
Cl(1)-Pd(1)-Pd(3)	53.045(18)
Cl(1)-Pd(3)-Pd(1)	53.085(18)
Pd(1)-P(2)-Pd(2)	80.850(3)
P(1)-Pd(2)-P(2)	98.162(3)
P(2)-Pd(1)-P(4)	101.721(3)
Pd(1)-Pd(2)-Pd(3)	59.111(7)

Table S10 Crystal data and structural refinement for $[Pd_3(\mu\text{-}Cl)(\mu\text{-}PPh_2)_2(PPh_3)_3][SbF_6]$ (ijsf1923).

Identification code	***ijsf1923* (CCDC2281111)**
Empirical formula	$C_{78}H_{65}ClF_6P_5Pd_3Sb$
Formula weight	1747.55
Temperature/K	110.00(10)
Crystal system	monoclinic
Space group	$P2_1/c$
a/Å	15.28922(15)
b/Å	16.70934(13)
c/Å	27.2586(2)
α/°	90
β/°	93.5087(8)
γ/°	90
Volume/Å^3	6950.79(11)
Z	4
ρ_{calc}g/cm^3	1.670
μ/mm^{-1}	11.152
F(000)	3472.0
Crystal size/mm^3	0.159 × 0.127 × 0.036
Radiation	CuKα (λ = 1.54184)
2Θ range for data collection/°	7.846 to 142.428
Index ranges	-18 ≤ h ≤ 17, -20 ≤ k ≤ 10, -33 ≤ l ≤ 32
Reflections collected	28086
Independent reflections	13206 [R_{int} = 0.0286, R_{sigma} = 0.0361]
Data/restraints/parameters	13206/0/847
Goodness-of-fit on F^2	1.033
Final R indexes [I>=2σ (I)]	R_1 = 0.0302, wR_2 = 0.0744
Final R indexes [all data]	R_1 = 0.0365, wR_2 = 0.0789
Largest diff. peak/hole / e Å^{-3}	1.16/-0.85

5 Literature Comparisons

Table S11 Comparison of palladium normalised rates to recent literature examples of low concentration and ligandless Pd (which may be expected to form nanoparticles Pd in solution). It should be noted the aryl halide and boronic acids do differ in some examples, but the purpose of the table is to give an indication of the ball park rates reported.

Reference	Pd type	ppm Pd (by moles)	Temperature / °C	Rate of aryl bromide conversion / mmol ArBr.(mmol Pd)$^{-1}$. min^{-1}
This work	Immob-Pd_3Cl_2	24	40	3
This work	Free Pd_3Cl_2	68	23	38
Koide and co-workers[19]	$Pd(OAc)_2$ ligandless	425	24	7
Hii *et al.* [20]	$Pd(OAc)_2$ ligandless	61	25	8
Novak and co-workers[21]	$Pd(PPh_2)_2Cl_2$	10	110	350

For context, ICP-OES of the filtered liquid phase for the standard (68 ppm, Immob-Pd_3Cl_2 catalyzed) SMCC reaction after > 50% conversion shows 0.39 ppm Pd (by moles) leached into solution (an upper limit as filtration may not remove completely the polymer resin). For this leached material to be the dominant contributor to catalysis would require it to be ~60 times more active than the room temperature example of dilute Pd reported in these reactions and even compared to the example at 110 °C the rate would need to be within a factor of 5, which is highly unlikely for the current work operating at 40 °C.

References

1 Scott, N. W.; Ford, M. J.; Schotes, C.; Parker, R. R.; Whitwood, A. C.; Fairlamb, I. J. S. The ubiquitous cross-coupling catalyst system 'Pd(OAc)$_2$'/2PPh$_3$ forms a unique dinuclear Pd I complex: an important entry point into catalytically competent cyclic Pd$_3$ clusters. *Chem. Sci.* **2019**, *10*, 7898-7906.

2 Berenblyum, A. S.; Aeeva, A. P.; Lakhman, L. I.; Moiseev, I. I. Mechanism of the formation of palladium complexes serving as catalysts in hydrogenation reactions: II. Reactivity of palladium phosphine halides towards molecular hydrogen. *J. Organomet. Chem.* **1982**, *234*, 237-248

3 Scott, N. W. J.; Ford, M. J.; Jeddi, N.; Eyles, A.; Simon, L.; Whitwood, A. C.; Tanner, T.; Willans, C. E.; Fairlamb, I. J. S. A Dichotomy in Cross-Coupling Site Selectivity in a Dihalogenated Heteroarene: Influence of Mononuclear Pd, Pd Clusters, and Pd Nanoparticles—the Case for Exploiting Pd Catalyst Speciation. *J. Am. Chem. Soc.* **2021**, *143*, 9682-9693.

4 Weddle, K. S.; Aiken, J. D.; Finke, R. G. Rh(0) Nanoclusters in Benzene Hydrogenation Catalysis: Kinetic and Mechanistic Evidence that a Putative [(C$_8$H$_{17}$)$_3$NCH$_3$]$^+$[RhCl$_4$]$^-$ Ion-Pair Catalyst Is Actually a Distribution of Cl$^-$ and [(C$_8$H$_{17}$)$_3$NCH$_3$]$^+$ Stabilized Rh(0) Nanoclusters. *J. Am. Chem. Soc.* **1998**, *120*, 5653–5666.

5 Chernyshev, V. M.; Astakhov, A. V.; Chikunov, I. E.; Tyurin, R. V.; Eremin, D. B.; Ranny, G. S.; Khrustalev, V. N.; Ananikov, V. P. Pd and Pt Catalyst Poisoning in the Study of Reaction Mechanisms: What Does the Hg Test Mean for Catalysis? *ACS Catal.* **2019**, *9*, 2984-2995.

6 Shell, T. A.; Mohler, D. L. Selective targeting of DNA for cleavage within DNA–histone assemblies by a spermine–[CpW(CO)$_3$Ph]$_2$ conjugate. *Org. Biomol. Chem.* **2005**, *3*, 3091-3093

7 Leszczyński, P.; Hofman, T.; Kaczorowska, E.; Adamczyk-Woźniak, A.; Sporzyński, A. Equilibria in the 3-(trifluoromethyl)phenylboronic acid - boroxine system. Solubility of the acid and its cyclic esters in organic solvents. *J. Organomet. Chem.* **2021**, *949*, 121947.

8 Tang, Q.; Xia, D.; Jin, X.; Zhang, Q.; Sun, X.-Q.; Wang, C. Re/Mg Bimetallic Tandem Catalysis for [4+2] Annulation of Benzamides and Alkynes via C-H/N-H Functionalization. *J. Am. Chem. Soc.* **2013**, *135*, 4628-4631

9 Du, Y.; Hyster, T. K.; Rovis, T. Rhodium(iii)-catalyzed oxidative carbonylation of benzamides with carbon monoxide. *Chem. Commun.* **2011**, *47*, 12074-12076.

10 Fu, F.; Xiang, J.; Cheng, H.; Cheng, L.; Chong, H.; Wang, S.; Li, P.; Wei, S.; Zhu, M.; Li, Y. A Robust and Efficient Pd3 Cluster Catalyst for the Suzuki Reaction and Its Odd Mechanism. *ACS Catal.* **2017**, *7*, 1860-1867.

11 Tromp, M.; van Bokhoven, J. A.; van Strijdonck, G. P. F.; van Leeuwen, P. W. N. M.; Koningsberger, D. C.; Ramaker, D. E. Probing the Molecular Orbitals and Charge Redistribution in Organometallic (PP)Pd(XX) Complexes. A Pd K-Edge XANES Study. *J. Am. Chem. Soc.* **2005**, *127*, 777-789.

12 Newville, M. IFEFFIT : interactive XAFS analysis and FEFF fitting. *J. Synchrotron. Radiat.* **2001**, *8*, 322-324.

13 Ravel, B.; Newville, M. ATHENA, ARTEMIS, HEPHAESTUS: data analysis for X-ray absorption spectroscopy using IFEFFIT. *J. Synchrotron. Radiat.* **2005**, *12*, 537-541.

14 Bouquillon, S.; du Moulinet d'Hardemare, A.; Averbuch-Pouchot, M.-T.; Hénin, F.; Muzart, J. Synthesis and characterization of monomeric and dimeric palladium(II)–ammonium complexes: their use for the catalytic oxidation of alcohols. *Polyhedron* **1999**, *18*, 3511-3516.

15 Appleby, K. M.; Dzotsi, E.; Scott, N. W.; Dexin, G.; Jeddi, N.; Whitwood, A. C.; Pridmore, N. E.; Hart, S.; Duckett, S. B.; Fairlamb, I. J. Bridging the Gap from Mononuclear Pd^{II} Precatalysts to Pd Nanoparticles: Identification of Intermediate Linear [Pd$_3$(XPh$_3$)$_4$]$^{2+}$ Clusters as Catalytic Species for Suzuki–Miyaura Couplings (X= P, As). *Organometallics* **2021**, *40*, 3560-3570.

16 Dolomanov, O. V.; Bourhis, L. J.; Gildea, R. J.; Howard, J. A. K.; Puschmann, H. OLEX2: a complete structure solution, refinement and analysis program. *J. Appl. Crystallogr.* **2009**, *42*, 339-341

17 Sheldrick, G. M. SHELXT - integrated space-group and crystal-structure determination. *Acta Crystallogr A Found Adv* **2015**, *71*, 3-8

18 Sheldrick, G. M. Crystal structure refinement with SHELXL. *Acta Crystallogr. C Struct. Chem.*

2015, *71*, 3-8

19 Vinod, J.; Wanner, A.; James, E.; Koide, K. Fluorometric study on the amine-catalysed Suzuki-Miyaura coupling, *Nat. Catal.* **2021**, *4*, 999-1001.

20 Adrio, L. A.; Nguyen, B. N.; Guilera, G.; Livingston, A. G.; Hii, K. K. Speciation of $Pd(OAc)_2$ in ligandless Suzuki–Miyaura reactions. *Cat. Sci. Technol.* **2012**, *2*, 316-323.

21 Novák, Z.; Adamik, R.; Csenki, J. T.; Béke, F.; Gavaldik, R.; Varga, B.; Nagy, B.; May, Z.; Daru, J.; Gonda, Z.; Tolnai, G. L. Revisiting the amine-catalysed cross-coupling. *Nat. Catal.* **2021**, *4*, 991–993.
